# Lewis Acid–Base Adducts of α-Amino Isobutyric Acid-Derived Silaheterocycles and Amines

**DOI:** 10.3390/molecules30173501

**Published:** 2025-08-26

**Authors:** Anne Seidel, Erica Brendler, Ana Torvisco, Roland Fischer, Jörg Wagler

**Affiliations:** 1 Institut für Anorganische Chemie, TU Bergakademie Freiberg, 09596 Freiberg, Germany; anne.seidel@chemie.tu-freiberg.de; 2 Institut für Analytische Chemie, TU Bergakademie Freiberg, 09596 Freiberg, Germany; erica.brendler@chemie.tu-freiberg.de; 3 Institut für Anorganische Chemie, TU Graz, 8010 Graz, Austria; ana.torviscogomez@tugraz.at (A.T.); roland.fischer@tugraz.at (R.F.)

**Keywords:** aminosilanes, bidentate ligands, hypercoordination, Lewis acid–base adducts, solid-state NMR, X-ray diffraction

## Abstract

The 1:1 stoichiometric reactions of α-amino isobutyric acid (H_2_Aib) and diaminosilanes of the type Si*RR*′(N*R*^1^*R*^2^)_2_ (SiMe_2_(imidazol-1-yl)_2_, SiMe_2_(NH*n*Pr)_2_, and Si*RR*′(pyrrolidin-1-yl)_2_ with *R*,*R*′ = Me,Me, Me,H, Me,Vi, and Et,Et) afforded the pentacoordinate silicon complexes (Aib)Si*RR*′(HN*R*^1^*R*^2^) with the release of one equivalent of HN*R*^1^*R*^2^. Single-crystal X-ray diffraction analyses confirmed the coordination of the N-donor Lewis base (i.e., imidazole, *n*-propylamine, and pyrrolidine, respectively) in an axial position of the distorted trigonal-bipyramidal Si-coordination sphere, *trans* to the carboxylate O atom of the Si-chelating Aib-dianion. The N–H moieties of the adduct-forming Lewis bases are involved in N–H⋯O hydrogen bonds with carboxylate groups of adjacent complex molecules, thus supporting the supramolecular structures of these adducts. The equatorially bound NH group of the Aib-dianion is involved in N–H⋯O hydrogen bonds in most cases, and it gives rise to residual dipolar coupling of the ^14^N nucleus with its directly bound atoms C and Si, thus causing characteristic shapes of both the ^29^Si and ^13^C NMR signals of these two atoms in the solid-state spectra. In contrast to the adduct-formation reactions, the analogous conversion of H_2_Aib and SiMe_2_(NH*t*Bu)_2_ did not afford an amine adduct. Instead, a second equivalent of H_2_Aib entered the reaction, and the ionic silicon complex [*t*BuNH_3_]^+^[(Aib)_2_SiMe]^−^ was obtained and characterized by crystallography and solution NMR spectroscopy.

## 1. Introduction

**Figure 1:**
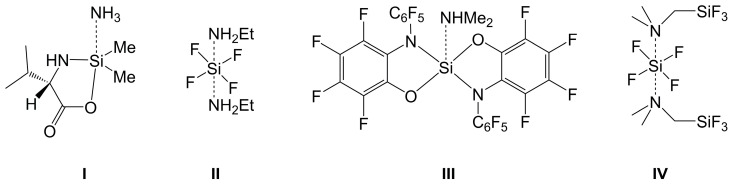
Molecular drawings of crystallographically characterized hypercoordinate Si complexes, which are adducts of ammonia (**I** [8]), a primary amine (**II** [9]), a secondary amine (**III** [10]), and a tertiary amine (**IV** [11]).

**Scheme 1:**
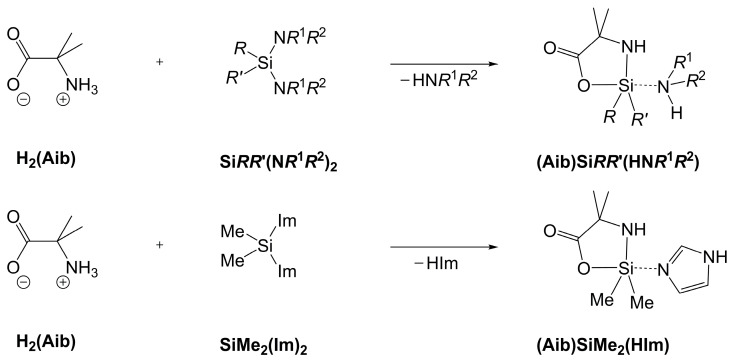
Generic scheme of the syntheses of amine adducts applied in the current work (**top**) and the related reaction applied to the imidazolyl silane SiMe_2_(Im)_2_ (**bottom**).

Silicon compounds with Si coordination numbers greater than four (so-called hypercoordinate silicon compounds) have attracted researchers’ interest for many decades [1,2,3,4,5,6]. Even though the ammonia adduct [SiF_4_(NH_3_)_2_], first reported in 1812 [7], is among the pioneering compounds in this broad field, only a few molecular structures of those penta- or hexacoordinate Si(IV) compounds have been reported to date, which represent ammonia- and monodentate amine adducts, i.e., complexes **I** [8], **II** [9], **III** [10], and **IV** [11] (Figure 1). They serve as the scarce portfolio of other Si adducts with ammonia, a primary, a secondary, and a tertiary amine, respectively. In this context, the adduct (L-Val)SiMe_2_(NH_3_) **I** [8] attracted our particular interest for the following two reasons: (1) This ammonia adduct forms under mild conditions (room temperature, in chloroform solution) in spite of the Si-bound electron releasing methyl substituents, which can be expected to dampen the Si atom’s Lewis acidity. (2) It represents a silicon complex of an α-amino acid anion, a scarcely explored class of hypercoordinate silicon complexes [12]. In a previous study, we showed that the (*αA*)SiMe_2_ motif (*αA* = di-anion of an α-amino acid) may support the formation of related adducts, with *N*-methylimidazole employed as the additional Lewis base (*LB*) [13]. In that initial study, we used the aminosilane SiMe_2_(NH*t*Bu)_2_ as a starting material, aiming for a sterically demanding amine leaving group that does not compete with other Lewis bases in adduct formation. In the current work, we employ a variety of aminosilanes of the type Si*RR*′(N*R*^1^*R*^2^)_2_ (*R*,*R*’ = a selection of hydrocarbyl and H; *R*^1^,*R*^2^ = alkyl, H) as the exclusive source of Lewis bases for adduct formation, utilizing 2-aminoisobutyric acid (H_2_Aib) as the constant α-amino-acid-derived ligand (Scheme 1). This particular amino acid was chosen because of its low tendency for dipeptide formation, as peptide formation has been reported as a side reaction when working with silylated proteinogenic α-amino acids [12,14].

## 2. Results and Discussion

### 2.1. Syntheses and Molecular Structures of Lewis–Base Adducts of the Type (Aib)SiRR′(LB)

The adducts of the type (Aib)Si*RR*′(HN*R*^1^*R*^2^), as well as the related imidazole adduct (Aib)SiMe_2_(HIm), were prepared from α-isobutyric acid and the respective silane in chloroform, according to Scheme 1. In contrast to adducts of tertiary amines and N-donor heterocycles devoid of an NH group (such as *N*-methylimidazole), the route of Scheme 1 can be applied to primary and secondary amines as well as to NH-functionalized heterocycles such as imidazole as additional Lewis basic donor molecules. (Note: in the latter case, the location of the NH moiety of the adduct-forming Lewis base is not at the Si-bound N atom, as demonstrated in Scheme 1, bottom.) The crystalline products (as well as the imidazolyl silane starting materials Me_3_Si(Im) and Me_2_Si(Im)_2_) were characterized by single-crystal X-ray diffraction analyses (cf. Appendix A, Table A1, Table A2, Table A3, Table A4 and Table A5). More details of the molecular structures of Me_3_Si(Im) and Me_2_Si(Im)_2_ are listed in Appendix B.

**Figure 2:**
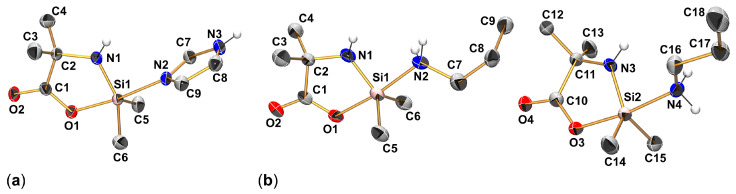
Molecular structures of (Aib)SiMe_2_(*LB*) adducts (from their respective crystal structures) shown with thermal ellipsoids at the 50% level, with non-hydrogen atoms labeled and C-bound H atoms omitted for clarity. (**a**) Molecular structure of (Aib)SiMe_2_(HIm) from the crystal structure of its chloroform solvate (Aib)SiMe_2_(HIm)·CHCl_3_. (**b**) Molecular structures of the two crystallographically independent molecules of (Aib)SiMe_2_(H_2_N*n*Pr). One *n*-propyl group is disordered (C8–C9); only the part of predominant site occupancy (with s.o.f. 0.865(7)) is shown.

**Figure 3:**
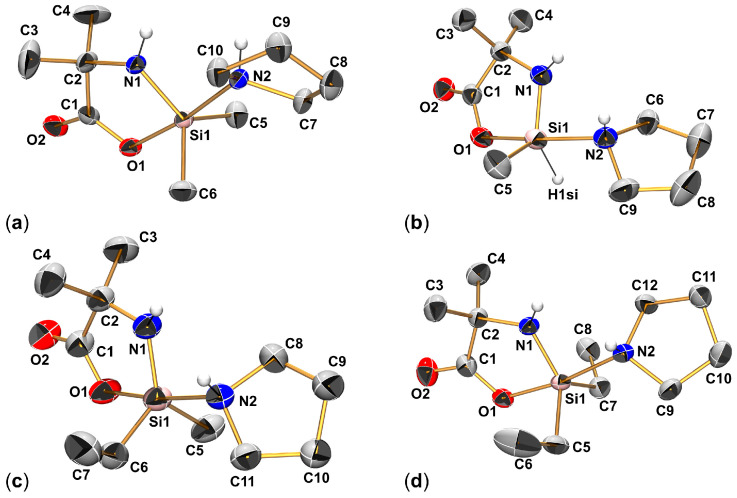
Molecular structures of pyrrolidine adducts (Aib)Si*RR*′(HPyr) from their respective crystal structures shown with thermal ellipsoids at the 50% level, with non-hydrogen atoms labeled and C-bound H atoms omitted for clarity. (**a**) Molecular structure of (Aib)SiMe_2_(HPyr). (**b**) Molecular structure of (Aib)SiMeH(HPyr). (**c**) Molecular structure of one of the two crystallographically independent molecules in the structure of (Aib)SiMeVi(HPyr). (**d**) Molecular structure of (Aib)SiEt_2_(HPyr).

The amino acid starting material, i.e., α-amino isobutyric acid H_2_Aib, is essentially insoluble in chloroform, whereas the silanes used are either liquids or well-soluble solids. Thus, the dissolution of H_2_Aib was an indicator of the progress of the conversion of starting materials. The choice of starting materials Me_2_Si(Im)_2_, Me_2_Si(NH*n*Pr)_2_, and Me_2_Si(Pyr)_2_ (Pyr = pyrrolidin-1-yl) gave access to adducts of an N-donor heterocycle, a primary amine, and a secondary amine, i.e., (Aib)SiMe_2_(HIm) (as a chloroform solvate (Aib)SiMe_2_(HIm)·CHCl_3_), (Aib)SiMe_2_(H_2_N*n*Pr), and (Aib)SiMe_2_(HPyr), respectively (Figure 2 and Figure 3). The latter amine, pyrrolidine, was chosen to investigate the effect of Si-bound substituents *R*,*R*′ further, and we succeeded in crystallizing the series of adducts (Aib)SiMeH(HPyr), (Aib)SiMeVi(HPyr), and (Aib)SiEt_2_(HPyr) (Figure 3). In addition, upon cooling of the filtrates obtained upon harvesting of (Aib)SiMe_2_(HPyr) and (Aib)SiMeH(HPyr), chloroform solvates of those compounds were obtained, and their structures were determined as well.

Table 1 contains selected bond lengths and angles of these adducts, complemented by the corresponding parameters of (Aib)SiMe_2_(NMI) [13]. In principle, the Si coordination spheres are very similar to one another within this class of compounds. The geometry parameter τ_5_ [15], which may range between 1 (for perfect trigonal-bipyramidal) and 0 (for square-based pyramidal coordination geometry), is essentially 0.8 for the set of compounds. This indicates Si-coordination spheres close to trigonal-bipyramidal shapes. The donor atom of the additional Lewis base (N2) occupies an axial position *trans* to the carboxylate O atom O1, spanning an axial angle in the narrow range 170–174 deg. Variations in the Lewis base hardly alter the bond lengths on this axis. Lowering or increasing the steric demand of equatorial positions (i.e., replacing SiMe_2_ by SiMeH or SiEt_2_, respectively) leads to some shortening or lengthening, respectively, of the Si1–N2 bond. As previously shown for related compounds such as (Val-Val)SiMe_2_, which feature the Lewis base (NH_2_ group) as part of a di-anionic dipeptide ligand [12], this structural change has a greater impact on the axial angle (greater deviation from linearity), whereas the axial bond lengths remain very similar.

With respect to the two Si–N bonds in each molecule, the axial bond (to the additional Lewis base) is significantly longer than the equatorial Si–N bond. Whereas the N atom of the former is tetracoordinate and acts as a lone pair donor toward Si in a formally dative bond, the N atom of the latter is tricoordinate and features a formally covalent Si–N bond as one out of its three bonds. Cota et al. reported a crystal structure of a pentacoordinate Si complex that features both an Si–NH*R* and an Si⋯NH_2_*R* (*R* = the backbone of the α-amino acid (S)-*tert*-leucine) [14], and even in the related positions within the Si coordination sphere (both equatorial), the formally dative Si⋯N bond is significantly longer than the formally covalent Si–N bond (1.884(1) vs. 1.704(1) Å). Axial positioning of a donor atom adds to bond lengthening. A silicon complex of the type [(O,N,N,O)SiMe]^+^Cl^−^, which features a tetradentate (O,N,N,O) salen-type ligand and a pentacoordinate Si atom with an axial and an equatorial Si⋯N bond with bond lengths of 1.937(2) and 1.846(2) Å, respectively, serves as an example [16]. This effect can be attributed to the 3c4e-bonding in axial positions, which has, for example, been analyzed for Si⋯N bonds in silatranes [17].

**Figure 4:**
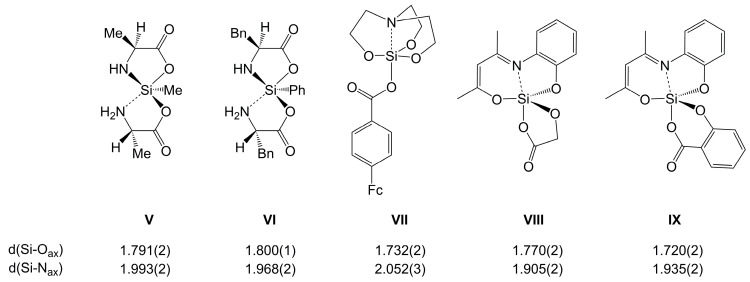
Examples of crystallographically characterized pentacoordinate Si complexes with axial arrangement of an N-donor Lewis base and a carboxylate. (In compound **VII**: Fc = ferrocenyl.) The axial bond lengths (in Å) are shown below the respective molecule.

Figure 4 shows examples of other classes of pentacoordinate Si complexes, which feature an axial arrangement of an amine or imine N atom *trans* to an Si-bound carboxylate. Compounds **V** and **VI** [14], which also feature a di-anion of an amino acid as a constituting part of the molecule, reveal shorter axial Si–O and Si–N bonds. We interpret this as an effect of the enhanced Lewis acidity of the Si atoms (in contrast to the Si atoms of the compounds listed in Table 1, they bear only one hydrocarbyl group). The Si–N bond length encountered with the carboxylate substituted silatrane **VII** [18] is similar to those of the Lewis-base adducts of our work, even though the Si atom carries rather electronegative atoms only. The silatrane cage itself may inhibit noticeable bond shortening. Even in the dimethyl ether stabilized cationic silatrane derivative [(N(CH_2_CH_2_O)_3_Si(OMe_2_)]^+^[BF_4_]^−^, the Si–N bond is as long as 1.965(5) Å in spite of the longer *trans*-disposed Si–O bond of 1.830(4) Å [19]. In **VII**, the bond shortening effect of the Si-atom’s Lewis acidity is reflected by the short axial Si–O bond only. Compounds **VIII** and **IX** [20] reveal that this different N-donor system is capable of forming shorter axial Si–N bonds at a pentacoordinate SiO_4_ center. Comparison of the related molecules **VIII** and **IX**, which feature the carboxylate in a five- and a six-membered silacycle, respectively, indicates that carboxylates in five-membered silacycles may be prone to Si–O bond lengthening, thus supporting *trans*-Si–N bond shortening.

**Figure 5:**
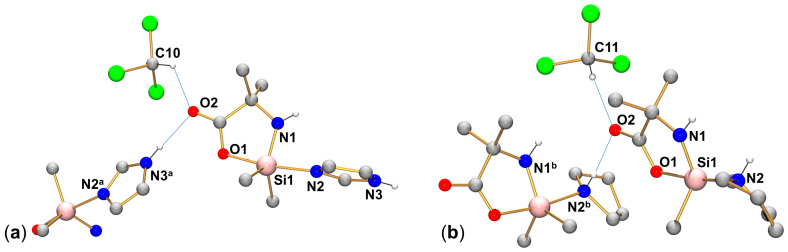
Ball-and-stick representations of the intramolecular C–H⋯O and N–H⋯O contacts at the carbonyl groups encountered in the crystal structures of (**a**) (Aib)SiMe_2_(HIm)·(CHCl_3_) and (**b**) 2[(Aib)SiMe_2_(HPyr)]·3(CHCl_3_). The H⋯O contacts are represented by blue lines. Symmetry equivalent atoms of adjacent molecules adhere to the symmetry operations ^a^ = x, 1.5 − y, −0.5 + z; ^b^ = 0.5 − x, y, 0.5 + z.

Besides the very similar intramolecular features within the class of α-amino acid (*αA*) derived Lewis-base (*LB*) adducts (*αA*)Si*RR*′(*LB*), their intermolecular interactions in the crystal structures provide some further insights into differences between the individual compounds and into effects that contribute to the stabilization of these adducts in the solid state. In contrast to the NMI adduct (Aib)SiMe_2_(NMI) [13], which was obtained as a chloroform solvate that melts below room temperature, the amine adducts obtained in the current study exhibit markedly higher melting points. Intermolecular bonding patterns, i.e., intermolecular N–H⋯O hydrogen bonds, provide an explanation. Whereas NMI is devoid of an N–H moiety and the single (Aib)SiMe_2_-bound N–H moiety of (Aib)SiMe_2_(NMI) is not involved in N–H⋯O hydrogen bonds, the combinations of the (Aib)SiMe_2_-bound N–H moiety and other N–H groups (i.e., from the adduct-forming amine) give rise to interactions with the carboxyl groups of adjacent molecules. Imidazole adduct (Aib)SiMe_2_(HIm)·(CHCl_3_) (Figure 5a) still features chloroform in the crystal structure in a C–H⋯O contact to the carbonyl O atom O2, but the imidazole NH group of an adjacent molecule is involved in a hydrogen bond with O2 as well, thus interconnecting the adduct molecules (Aib)SiMe_2_(HIm). In the chloroform solvate 2[(Aib)SiMe_2_(HPyr)]·3(CHCl_3_) (Figure 5b), a similar structural feature is encountered, i.e., the carbonyl O atom O2 being involved in a chloroform C–H⋯O contact and in an N–H⋯O hydrogen bond with the amine moiety of an adjacent adduct molecule. In both cases, the NH groups of the amino acid dianion (N1–H) are not involved in hydrogen bonding.

**Figure 6:**
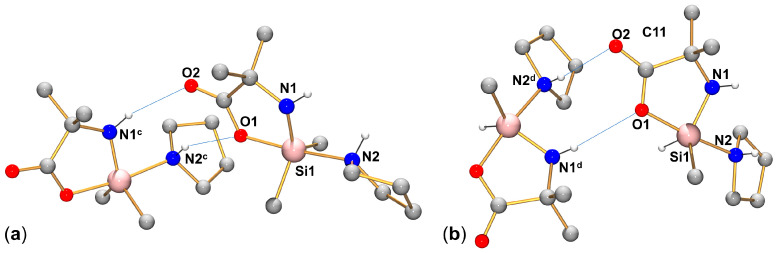
Ball-and-stick representations of the intramolecular N–H⋯O contacts at the carbonyl groups encountered in the crystal structures of (**a**) (Aib)SiMe_2_(HPyr) and (**b**) (Aib)SiMeH(HPyr). The H⋯O contacts are represented by blue lines. Symmetry equivalent atoms of adjacent molecules adhere to the symmetry operations ^c^ = −0.5 + x, 1.5 − y, −0.5 + z; ^d^ = x, 1 − y, 0.5 + z.

**Figure 7:**
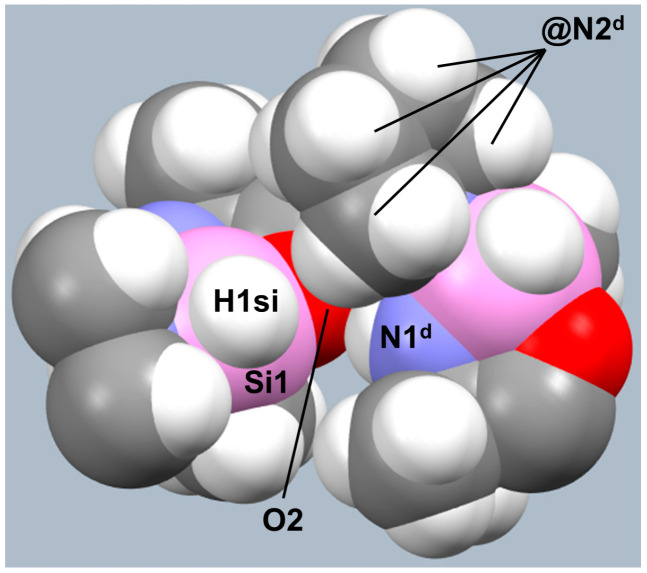
Space-fill representation of a section around Si1 in (Aib)SiMeH(HPyr). The labels correspond to those in Figure 6b.

In contrast to the situation in chloroform solvate 2[(Aib)SiMe_2_(HPyr)]·3(CHCl_3_), the crystal structures of the other pyrrolidine adducts of this study feature H-bonding of the COO-groups in which both the amine NH and the amino acid’s NH are involved (Figure 6). The pattern shown in Figure 6a, an $R_{2}^{2}(8)$ motif [21], shown for (Aib)SiMe_2_(HPyr) as a representative example, is also a feature of the crystal structures of (Aib)SiMeVi(HPyr) and (Aib)SiEt_2_(HPyr). In these cases, the amine NH is involved in a hydrogen bond with the Si-bound O atom of the carboxylate group, whereas the amino acid’s NH binds to the carbonyl O atom. The two chemically different hydrogen bond donors swap roles in (Aib)SiMeH(HPyr) (Figure 6b). Essentially, the same feature is also encountered in the crystal structure of the chloroform solvate 2[(Aib)SiMeH(HPyr)]·3(CHCl_3_). In the latter, which features two crystallographically independent molecules of (Aib)SiMeH(HPyr) in the asymmetric unit, the N–H⋯O contacts are complemented by a chloroform C–H⋯O contact to the carbonyl O atom in both cases. A space-fill representation of the closer proximity of this H-bond motif in (Aib)SiMeH(HPyr) (Figure 7) provides an explanation as to why the H-bond donors may swap roles in this particular case. In this arrangement, the pyrrolidine ring (at N2^d^ in the case shown in Figure 6b) is found near one of the Si-bound substituents, and the Si-bound H atom of (Aib)SiMeH(HPyr) (denoted as H1si in Figure 7) can tolerate this close approach, whereas the steric demand of larger substituents, e.g., Si-bound alkyl groups, would pose a hindrance.

**Figure 8:**
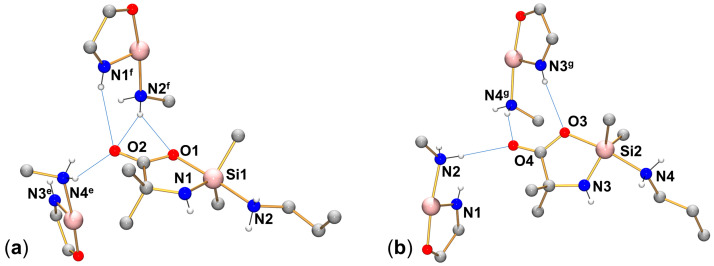
Ball-and-stick representations of the intramolecular N–H⋯O contacts at the carbonyl groups encountered in the crystal structure of (Aib)SiMe_2_(H_2_N*n*Pr), sub-figures (**a**,**b**) represent the situations for the two crystallographically independent molecules. The H⋯O contacts are represented by blue lines. Symmetry equivalent atoms of adjacent molecules adhere to the symmetry operations ^e^ = x, 1 + y, z; ^f^ = −0.5 + x, 2 − y, −0.5 + z; ^g^ = 0.5 + x, 1 − y, 0.5 + z.

Last but not least, compound (Aib)SiMe_2_(H_2_N*n*Pr) engages the additional NH moiety of its primary amine unit in intermolecular N–H⋯O bonding as well. Figure 8 shows the N–H⋯O H-bond situations for the two crystallographically independent molecules in the crystal structure of (Aib)SiMe_2_(H_2_N*n*Pr). In principle, the two O atoms of the carboxyl group are H-bound to the two chemically different N–H donor moieties (amine- and amino acid-based) of an adjacent molecule. Moreover, the second N–H group of the primary amine moiety, which is not engaged in this 2+2-H-bonding, is involved in another H-contact with a carbonyl O atom of another adjacent molecule. Thus, in each of the two crystallographically independent molecules, the carboxyl group is involved in H-bonds with two NH moieties of one adjacent molecule and in an additional N–H⋯O bond with another adjacent molecule. In spite of this general pattern, there are noticeable differences between the H-bond systems of the two independent molecules. The orientation of the two-fold N–H-donor differs, i.e., in molecule 1, the carbonyl O atom (O2) binds to the amino acid-based N1^f^–H, whereas the Si-bound carboxylate O1 binds to an amine N2^f^–H, the latter in a bifurcated manner. In molecule 2, the carbonyl O atom (O4) binds to an amine N4^g^–H, and the amino acid-based N3^g^–H binds to the Si-bound carboxylate O3 in a rather linear manner, not bifurcated. The different electronic situations of these two independent molecules in the crystal structure have an influence on the NMR spectroscopic properties, i.e., two well-resolved signals of the same intensity are observed in the ^29^Si solid-state NMR spectrum of (Aib)SiMe_2_(H_2_N*n*Pr) (cf. Section 2.2). (C⋯O and N⋯O distances of the herein-mentioned intermolecular contacts are listed in Table 2).

### 2.2. Spectroscopic Characterization (^29^Si and ^13^C Solid-State NMR Spectroscopy) of Lewis-Base Adducts of the Type (Aib)SiRR′(LB)

Characterization of the compounds presented in Section 2.1 in solution is hampered by effects of dynamic equilibria of formation and dissociation of the Lewis-base adducts (e.g., signal broadening and highly concentration-dependent signal positions). We have previously shown this effect for NMI adducts, e.g., (Aib)SiMe_2_(NMI) [13]. Therefore, we characterized the adducts in the solid state in order to correlate the spectroscopic features with their molecular structures. As a means of fingerprint characterization, IR spectra of those adducts that are stable at room temperature (i.e., (Aib)SiMe_2_(HIm)·(CHCl_3_), (Aib)SiMe_2_(H_2_N*n*Pr), (Aib)SiMe_2_(HPyr), (Aib)SiMeH(HPyr), (Aib)SiMeVi(HPyr), and (Aib)SiEt_2_(HPyr)) were recorded and can be found in the Supplementary Materials (cf. Figures S19–S24). From these data, we only emphasize that a characteristic band at 2044 cm^−1^ in the spectrum of (Aib)SiMeH(HPyr) points to the presence of the Si–H moiety in this particular compound. ^29^Si and ^13^C solid-state NMR spectroscopy were our methods of choice, which allow for unambiguous signal assignment and, to some extent, give an indication of the purity of the compounds obtained (cf. Figures S1–S15 for the individual spectra).

**Figure 9:**
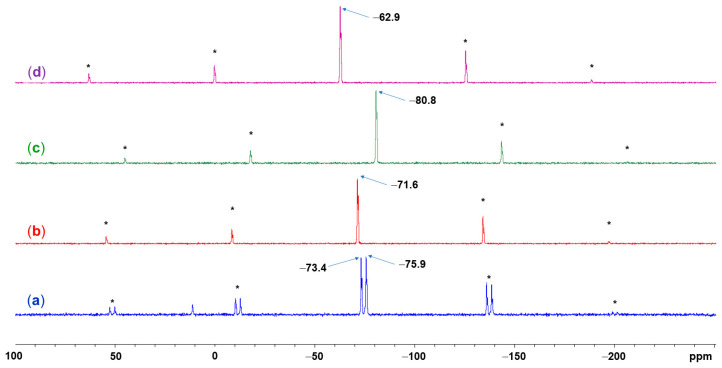
^29^Si CP/MAS NMR spectra of (**a**) (Aib)SiMe_2_(H_2_N*n*Pr), (**b**) (Aib)SiMe_2_(HPyr), (**c**) (Aib)SiMeH(HPyr), and (**d**) (Aib)SiEt_2_(HPyr), recorded at a magic angle spinning frequency of 5 kHz. The asterisks (*) denote the positions of spinning side bands. The set of two signals of (**a**) (Aib)SiMe_2_(H_2_N*n*Pr) is in accordance with the presence of two crystallographically independent molecules. (The small signal at ca. 11 ppm in the spectrum of (**a**) (Aib)SiMe_2_(H_2_N*n*Pr) arises from small amounts of a contaminant that has not been identified, yet.) The chemical shift values shown are the positions of the maxima of the isotropic signal (*δ*_iso(max)_). Because of the asymmetric signal shape, which arises from ^14^N residual dipolar coupling, *δ*_iso(max)_ is slightly off the true isotropic chemical shift *δ*_iso_.

The ^29^Si NMR spectra (CP/MAS cross-polarization/magic angle spinning) of the solids exhibit the expected signal patterns, i.e., isotropic chemical shifts *δ*_iso_ in a range characteristic for pentacoordinate silicon compounds and, indicated by the spinning side bands, pronounced chemical shift anisotropy. Figure 9a–d shows the spectra of (Aib)SiMe_2_(H_2_N*n*Pr), (Aib)SiMe_2_(HPyr), (Aib)SiMeH(HPyr), and (Aib)SiEt_2_(HPyr), respectively, as representative examples. The corresponding spectra of the other compounds ((Aib)SiMe_2_(HIm)·(CHCl_3_), *δ*_iso(max)_ −71.5; (Aib)SiMeVi(HPyr), *δ*_iso(max)_ −81.2) are contained in the Supplementary Materials. (Note: because of the asymmetric signal shape, which arises from ^14^N residual dipolar coupling, the position of the maximum of the isotropic signal, *δ*_iso(max)_, is slightly off the true isotropic chemical shift *δ*_iso_).

For this set of Aib-derived compounds, the isotropic chemical shifts mainly depend on the Si-bound hydrocarbyl or H substituents. Whereas the signals of the SiMe_2_ derivatives emerge in a rather narrow range (−71.5, −71.6, −73.4, and −75.9 for the complexes with the Lewis bases HIm, HPyr, H_2_N*n*Pr, respectively), changes in the Si–C or Si–H substitution pattern cause greater changes in the ^29^Si chemical shift. Replacing one Me group of (Aib)SiMe_2_(HPyr) by H or vinyl causes an upfield shift in the signal by 9.2 or 9.6 ppm, respectively. Similar effects (to an even greater extent) are known from other pentacoordinate Si-compounds such as silatranes, i.e., isotropic ^29^Si NMR shifts for solid methyl-, H-, and vinylsilatrane were reported as −71.0, −86.0, and −84.0 [22], respectively. The different electronic situations of these substituents can be regarded as the major cause. Replacing the methyl by ethyl groups causes a noticeable downfield shift (by 8.7 ppm). Whereas both substituents are simple alkyl groups and in case of silicon compounds such as methyl- and ethylsilatrane the ^29^Si NMR shifts in the solids are similar (−71.0 and −70.0, respectively [22]), the molecular structures of these two compounds indicate that the SiEt_2_-derivative exhibits a longer bond to the pyrrolidine N atom (Si–N2 2.08 Å in (Aib)SiEt_2_(HPyr), 1.99–2.04 Å in the other compounds, cf. Table 1). Thus, we consider this lengthening of the Si–N2 bond as a major cause of the downfield shifted ^29^Si NMR signal of this particular compound. From spinning side band spectra recorded at lower spinning frequency (3 kHz or 2 kHz), the chemical shift anisotropy (CSA) tensors were analyzed (for details see the Supplementary Materials, Table S2 and Figures S28–S33). Each of the six compounds analyzed by ^29^Si CP/MAS NMR spectroscopy exhibits a rather wide span (Ω) of the CSA tensor. They range from 166 ppm ((Aib)SiMeH(HPyr)) to 216 ppm ((Aib)SiMe_2_(H_2_N*n*Pr)) and have negative skew in all cases (*κ* in the range from −0.70 to −0.96). These data are typical for pentacoordinate Si complexes with rather trigonal-bipyramidal Si-coordination spheres and two particularly electronegative donor atoms in axial positions. Analyses of CSA tensors of other pentacoordinate Si complexes have revealed that the negative skew results from poor shielding of the ^29^Si nucleus in the idealized direction of the trigonal-bipyramidal axis (principal component *δ*_11_), whereas the principal components *δ*_22_ and *δ*_33_ are associated with directions in or close to the equatorial plane and pronounced shielding [23,24,25,26].

**Figure 10:**
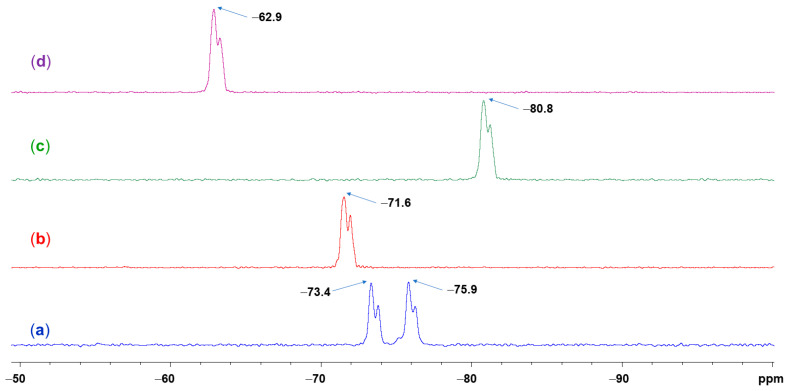
Magnified section of the ^29^Si CP/MAS NMR spectra of (**a**) (Aib)SiMe_2_(H_2_N*n*Pr), (**b**) (Aib)SiMe_2_(HPyr), (**c**) (Aib)SiMeH(HPyr), and (**d**) (Aib)SiEt_2_(HPyr); only the isotropic shift signals are shown. The chemical shift values shown are the positions of the maxima of the isotropic signal (*δ*_iso(max)_). Because of the asymmetric signal shape, which arises from ^14^N residual dipolar coupling, *δ*_iso(max)_ is slightly off the true isotropic chemical shift *δ*_iso_.

The signals (both the isotropic signals and spinning side bands) of the spectra in Figure 9 exhibit characteristic asymmetric coupling patterns, which are shown in the magnification for the isotropic signals in Figure 10. These patterns can be attributed to residual dipolar coupling with Si-bound nitrogen ^14^N. Such a coupling pattern was also observed with the ammonia adduct (Val)SiMe_2_(NH_3_) [8]. As adducts with different Lewis bases (such as NH_3_, NH_2_*n*Pr, pyrrolidine, and imidazole) exhibit essentially the same pattern, even in case of (Aib)SiEt_2_(HPyr), a compound with a rather long Si–N(pyrrolidine) bond, we attribute this effect to the equatorial NH group of the amino acid dianionic ligand. This is in accordance with the ratio of bond lengths Si–N(amino acid):Si–N(LB), which is ca. 1.2 (cf. Table 1). As the residual dipolar coupling and the distance *r* of the coupling nuclei scale by *r*^−3^ [27], the effect of the ^14^N of the amino acid should be more pronounced by a factor of 1.7. This effect of the amino acid’s ^14^N atom is finally confirmed in the ^13^C NMR spectra (vide infra), where the amino acid-derived ligand’s α-C atoms exhibit similar coupling patterns. (Note: the skew of the patterns is inverted because of the opposite sign of *γ*(^13^C) vs. *γ*(^29^Si), resulting in the opposite sign of the residual dipolar coupling constant [27].)

**Figure 11:**
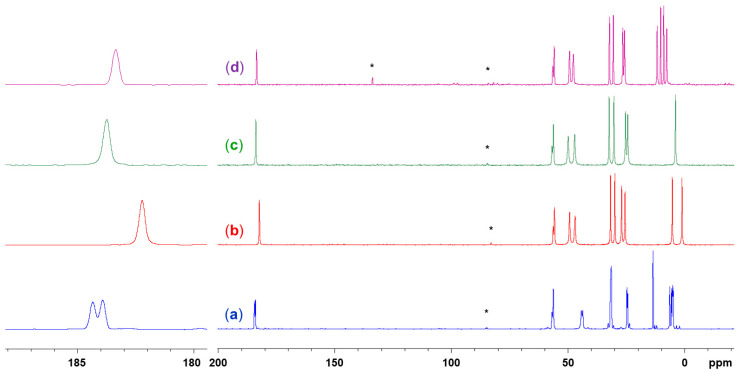
^13^C CP/MAS NMR spectra of (**a**) (Aib)SiMe_2_(H_2_N*n*Pr), (**b**) (Aib)SiMe_2_(HPyr), (**c**) (Aib)SiMeH(HPyr), and (**d**) (Aib)SiEt_2_(HPyr), recorded at a magic angle spinning frequency of 10, 10, 10, and 5 kHz, respectively. The asterisks (*) denote positions of spinning side bands of the ^13^C signals of the COO group. The two-fold set of signals of (**a**) (Aib)SiMe_2_(H_2_N*n*Pr) (for example, it can be seen in the magnification of the COO signal range 180–188 ppm, which is added to the left) is in accordance with the presence of two crystallographically independent molecules.

**Figure 12:**
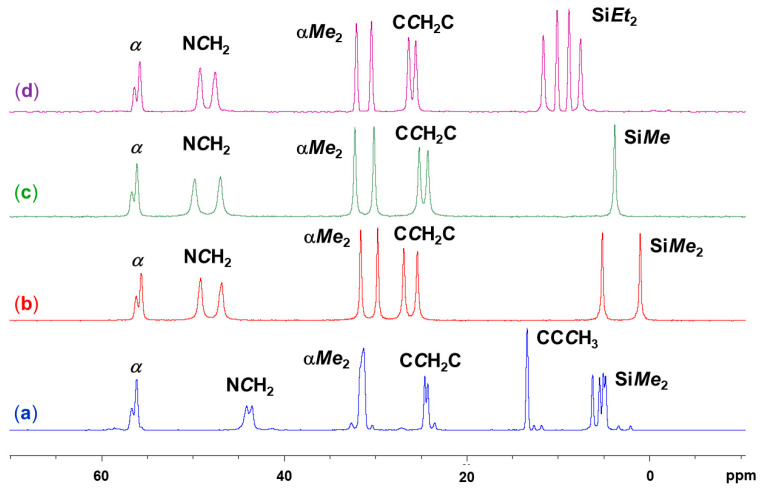
Magnified section of the ^13^C CP/MAS NMR spectra from Figure 11 of (**a**) (Aib)SiMe_2_(H_2_N*n*Pr), (**b**) (Aib)SiMe_2_(HPyr), (**c**) (Aib)SiMeH(HPyr), and (**d**) (Aib)SiEt_2_(HPyr) with assignment of groups of signals (the relevant atom or C-bearing group is italicized). Atom assignment α corresponds to the α-C atom of the Aib ligand.

The ^13^C NMR spectra of the solid adducts correspond to the crystal structures (Figure 11 and Figure 12a–d show the examples for (Aib)SiMe_2_(H_2_N*n*Pr), (Aib)SiMe_2_(HPyr), (Aib)SiMeH(HPyr), and (Aib)SiEt_2_(HPyr), respectively). The amino acid’s α-C atom gives rise to a signal around 56 ppm, which is markedly patterned by residual dipolar coupling with the neighboring ^14^N nucleus. In the cases of the pyrrolidine adducts (Figure 11b–d and Figure 12b–d), the number of signals of the individual groups corresponds to the number of crystallographically independent C atom sites. This is particularly pronounced for the two independent Si-Me groups of (Aib)SiMe_2_(HPyr), which are separated by Δ*δ*_iso_ 4.1 ppm. In the case of (Aib)SiMe_2_(H_2_N*n*Pr) (Figure 11a and Figure 12a), which features two independent molecules in the asymmetric unit, some of the individual signal positions (the two α-C atoms, the four α-C-bound methyl groups, and the two independent methyl groups of the *n*-propylamine ligands) are not resolved.

In the spectrum of (Aib)SiMeVi(HPyr) (cf. Figures S10–S12 in the Supplementary Materials), more signals arise for the individual groups. This observation can be explained by the disorder of the molecules in this structure. Even though the asymmetric unit features two independent molecules, their SiMe/SiVi positional disorder basically creates four different molecules in the crystal environment. The ratio of these molecules, however, may vary and does not need to adhere to the ratio of site occupancies found in the crystal used for X-ray diffraction analysis. In the latter, site occupancy ratios of 0.792(5):0.208(5) and 0.558(4):0.442(4) were refined for the two independent molecules. The corresponding spectrum of (Aib)SiMe_2_(HIm)·(CHCl_3_) (cf. Figure S1) features the expected signal of the solvent of crystallization (CHCl_3_ at *δ*_iso_ 80.0 ppm) in similar intensity as the imidazole C signals (at *δ*_iso_ 118.5, 125.5, 135.2 ppm), thus underlining the retention of the solvent of crystallization upon drying.

### 2.3. Reaction of H_2_Aib and SiMe_2_(NHtBu)_2_ Without an Additional Lewis Base

**Scheme 2:**
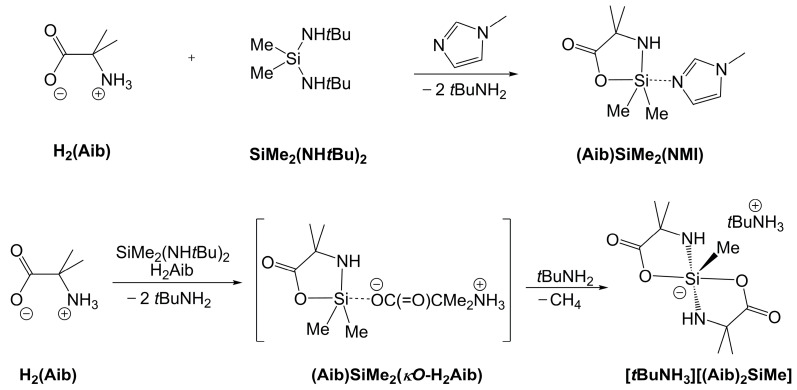
Reaction of H_2_Aib and SiMe_2_(NH*t*Bu)_2_ in the presence of NMI as an adduct-forming Lewis base [13] (**top**) and the related reaction in the absence of NMI (**bottom**).

**Figure 13:**
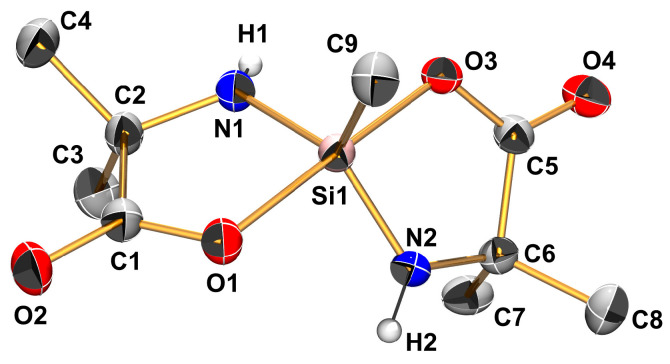
Molecular structure of the anion [(Aib)_2_SiMe]^−^ (from the crystal structure of [*t*BuNH_3_][(Aib)_2_SiMe]·(CHCl_3_)·(*t*BuNH_2_)) shown with thermal ellipsoids at the 50% level, with non-hydrogen atoms labeled and C-bound H atoms omitted for clarity. Selected bond lengths (Å) and angles (deg.): Si1–O1 1.8355(11), Si1–O3 1.8531(10), Si1–N1 1.7277(13), Si1–N2 1.7191(13), Si1–C9 1.8765(16), O1–Si1–O3 173.75(5), N1–Si1–N2 126.02(7), N1–Si1–C9 115.88(7), N2–Si1–C9 118.08(7).

In our previous study, we had employed the silane SiMe_2_(NH*t*Bu)_2_ as a starting material with a sterically demanding leaving group for the preparation of NMI adducts such as (Aib)SiMe_2_(NMI) (Scheme 2, top) [13]. In the context of the syntheses presented in Section 2.1, the question arises as to what product forms upon reaction of H_2_Aib and SiMe_2_(NH*t*Bu)_2_ in the absence of an additional Lewis base. The sterically demanding *t*Bu group of *t*BuNH_2_ should inhibit adduct formation. (In the Supplementary Materials, Figure S27, the space-fill view of a molecule of (Aib)SiMe_2_(H_2_N*n*Pr) indicates that replacement of the two remaining α-C-bound H atoms of the primary amine by two CH_3_ groups should be sterically unfavorable in such an adduct.) In the absence of NMI, H_2_Aib still dissolved in chloroform upon addition of SiMe_2_(NH*t*Bu)_2_ and stirring. In the ^29^Si NMR spectrum of the crude product solution, a variety of signals emerged in the typical range of tetracoordinate Si-compounds, but one prominent signal emerged at ca. −87 ppm, speaking for the formation of a pentacoordinate silicon compound. From this crude mixture, some crystals formed, and the X-ray diffraction analysis of such a crystal revealed the identity of this product as the ionic complex [*t*BuNH_3_]^+^[(Aib)_2_SiMe]^−^, which crystallized as a solvate [*t*BuNH_3_][(Aib)_2_SiMe]·(CHCl_3_)·(*t*BuNH_2_) (Figure 13, Table A5). As the Si atom carries two di-anionic (Aib) ligands and only one methyl group, this complex must have formed in a reaction as shown in Scheme 2, bottom. The carboxylate moiety of H_2_Aib (alternatively, of its mono-anion upon deprotonation by tBuNH_2_) represents the only alternative Lewis base in the system. (Its amino group, also bound to a tertiary C atom, should be unfavorable for adduct formation similar to *t*BuNH_2_.) Thus, we propose the formation of an adduct such as [(Aib)SiMe_2_(*κO*-H_2_Aib)] as the key intermediate, which eventually affords the ionic compound [*t*BuNH_3_][(Aib)_2_SiMe]. Using a synthesis under modified conditions (step-wise addition of H_2_Aib rather than mixing the two starting materials at once, cf. experimental section), we succeeded in isolating [*t*BuNH_3_][(Aib)_2_SiMe] in 24% yield. (The isolated compound, as a solution in DMSO-d6, gives rise to a ^29^Si NMR signal at −90 ppm.) Even though the cleavage of Si–C(alkyl) bonds by mild acids is rather unusual, Si–C cleavage with release of the corresponding hydrocarbon has been reported before with the syntheses of pentacoordinate Si complexes by di-anionic chelating ligands with formation of five-membered silacycles [28,29].

The Si atom of the anion [(Aib)_2_SiMe]^−^ is situated in a distorted trigonal-bipyramidal coordination sphere (geometry parameter τ_5_ = 0.80 [15]). With respect to this parameter τ_5_, it deviates from perfectly trigonal-bipyramidal to a similar extent as those of the amine adducts listed in Table 1. In terms of the widest equatorial angle, we point out that in both the amine adducts in Table 1 and in the anion [(Aib)_2_SiMe]^−^, this angle does not adhere to VSEPR. According to the latter, one would expect particular widening of the C–Si–C angle in the amine adducts and particular widening of the N–Si–C angles in the case of [(Aib)_2_SiMe]^−^. The structure of [(Aib)_2_SiMe]^−^ is closely related to the anionic spirosilicate moiety of the amino acid-derived zwitterionic spirosilicates reported by Tacke et al. [30,31]. The ^29^Si NMR shift observed with [*t*BuNH_3_][(Aib)_2_SiMe] (vide supra) is also similar to those of amino acid-derived zwitterionic spirosilicates, e.g., [(Gly)_2_Si-CH_2_-(NH-2,2,6,6-Me_4_C_5_H_6_] −91.9 ppm (in CD_2_Cl_2_) [30]. In contrast to [(Aib)_2_SiMe]^−^, their Si-bound methyl group is substituted by an ammonium cationic moiety (an *N*-protonated 2,2,6,6-tetramethylpiperidin-1-yl group). Nonetheless, their Si-coordination spheres exhibit similar features of axial O–Si–O angles in the range 173–180 deg. and particularly widened equatorial N–Si–N angles in the range of 124–131 deg. The Si–O and Si–N bond lengths of [(Aib)_2_SiMe]^−^ and the related zwitterionic spirosilicates [30,31] do not exhibit any greater differences. For the latter, they fall in the ranges 1.81–1.85 Å and 1.71–1.74 Å, respectively. The Si–C bonds, however, are markedly longer in the zwitterionic complexes (1.91–1.93 Å), whereas in [(Aib)_2_SiMe]^−^ (ca. 1.877 Å), it is closer to the lengths of Si–C(alkyl) bonds in other pentacoordinate Si complexes such as [(Aib)SiMe_2_(HPyr)] (1.874(2) and 1.875(2) Å), [(Aib)SiEt_2_(HPyr)] (1.880(2) and 1.882(2) Å), [(Ala)(HAla)SiMe] (1.840(2) Å) [14], [SiMe_2_H(NMI)_2_]^+^Cl^−^ (1.844(4) and 1.852(4) Å) [32], or [(PhCONNMe_2_)_2_SiMe]^+^(F_3_CSO_3_)^−^ (1.835(2) Å) [33].

## 3. Materials and Methods

### 3.1. General Considerations

Starting material SiMe_2_(NH*n*Pr)_2_ was prepared along the protocol described in [34], SiMe_2_(*t*BuNH)_2_ and SiMe_2_(Pyr)_2_ were prepared according to a published method from [35], and silanes SiMeH(Pyr)_2_, SiMeVi(Pyr)_2_, and SiEt_2_(Pyr)_2_ (prepared in a related manner) were available from a previous study [36]. The syntheses of SiMe_3_(Im) and SiMe_2_(Im)_2_ are briefly outlined in the Supplementary Materials. α-Aminoisobutyric acid (Roth, Karlsruhe, Germany, ≥97%), SiMe_2_Cl_2_ (Honeywell, Seelze, Germany, <98%), SiMe_3_Cl (Sigma-Aldrich, Steinheim, Germany, 99%), and imidazole (Roth, Karlsruhe, Germany, ≥99%) were used without further purification. *n*-propylamine (TCI, Eschborn, Germany, 98%), pyrrolidine (abcr, Karlsruhe, Germany 99%), and *tert*-butylamine (Thermo Fisher Scientific, Darmstadt, Germany, 99%) were distilled and stored under an Ar atmosphere prior to use. *n*-Hexane (VWR, Darmstadt, Germany, ≥99%), *n*-pentane (Honeywell, Seelze, Germany, ≥99.8%), and chloroform, stabilized with amylenes (Honeywell, Seelze, Germany, ≥99.5%), CDCl_3_ (Deutero, Kastellaun, Germany, 99.8%), and DMSO-d6 (ARMAR, Leipzig, Germany, 99.8%) were stored over activated molecular sieves (3 Å) for at least 7 days and used without further purification. All reactions were carried out under an atmosphere of dry argon utilizing standard Schlenk techniques. For syringe filtration, PTFE syringe filters of 15 mm diameter and 0.2 μm pore size (Roth, Karlsruhe, Germany) were used. Solution NMR spectra (^1^H, ^13^C, ^29^Si) were recorded on a Bruker Nanobay 400 MHz spectrometer. ^1^H, ^13^C, and ^29^Si chemical shifts are reported relative to Me_4_Si (0 ppm) as internal reference. Solid-state NMR experiments were performed on a Bruker Avance HD 400 MHz WB spectrometer using a 4 mm DVT CP/MAS probe with ZrO_2_ rotors. The chemical shift is reported relative to Me_4_Si (0 ppm) and was referenced externally for ^29^Si to octakistrimethylsiloxyoctasilsesquioxane Q_8_M_8_ (most upfield signal of Q^4^ groups at *δ*_iso_ = −109 ppm) and for ^13^C to adamantane (downfield signal, at *δ*_iso_ = 38.5 ppm). Cross polarization NMR experiments were carried out with 5 ms contact time for ^29^Si and 2 ms for ^13^C, and an 80% ramp was used for ^29^Si and ^13^C. Evaluation of the tensors of ^29^Si chemical shift anisotropy (CSA) from spinning side band spectra (for data, see the Supplementary Materials, Table S2 and Figures S28–S33) was performed with HBA [37] and DMFIT [38]. The order of the principal components *δ*_11,_ *δ*_22,_ *δ*_33_, as well as span Ω and skew *κ*, are reported according to the Herzfeld–Berger notation [39,40]. IR spectra (ATR) were recorded on a Spectrum 3 instrument (PerkinElmer, Waltham, MA, USA). For single-crystal X-ray diffraction analysis of SiMe_3_(Im), the compound was transferred into a glass capillary, which was then sealed, mounted on the goniometer. (The crystallization procedure was similar to our approach of crystallizing chlorosilanes for X-ray diffraction analyses [41].) For most of the other compounds reported in this work, a single crystal was selected under an inert oil and mounted on a glass capillary, and diffraction data were collected on a Stoe IPDS-2/2T diffractometer (STOE, Darmstadt, Germany) using Mo Kα-radiation. Data integration and absorption correction were performed with the STOE software XArea (version 2.3) and XShape (version 2.25), respectively. The data set of [*t*BuNH_3_][(Aib)_2_SiMe]·(CHCl_3_)·(*t*BuNH_2_) was collected on a Rigaku XtaLAB Synergy Dualflex HyPix-Arc 100 diffractometer using Cu Kα-radiation. Data integration and absorption correction were performed with CrysAlisPro. The structures were solved using SHELXT-2018/2 [42] and refined with the full-matrix least-squares methods of *F*^2^ against all reflections with SHELXL-2019/3 [43,44]. All non-hydrogen atoms were anisotropically refined, C-bound hydrogen atoms were isotropically refined in idealized positions (riding model), and N-bound hydrogen atoms were isotropically refined (bond length constraints were applied only when necessary). For details of data collection and refinement, see Appendix A, Table A1, Table A2, Table A3, Table A4 and Table A5. As a disorder of the pyrrolidine ring (C–C bond in 3,4-positions of this heterocycle disordered in a cross-wise manner) was encountered frequently with the herein reported pyrrolidine adducts, this disorder is briefly explained in the Supplementary Materials (cf. Figure S26 and Table S1) using the example of compound (Aib)SiMe_2_(HPyr). Details of the use of SQUEEZE for the treatment of solvent disorder in the case of the structure of 2[(Aib)SiMeH(HPyr)]·3(CHCl_3_) are listed with the data of structure refinement of this particular structure (cf. Table A5). Graphics of molecular structures were generated with ORTEP-3 [45,46], POV-Ray 3.7 [47], and Mercury 2021.3.0 [48].

### 3.2. Synthesis and Characterization

Compound (Aib)SiMe_2_(H_2_N*n*Pr): In a small Schlenk tube, equipped with a magnetic stirring bar, α-amino isobutyric acid (H_2_Aib, 500 mg, 4.85 mmol) was evacuated and then set under an argon atmosphere. Upon addition of chloroform (2 mL), the aminosilane (SiMe_2_(NH*n*Pr)_2_, 851 mg, 4.88 mmol) was added with a syringe, and stirring at room temperature was continued for 2 d. During that time, the fine crystals of the amino acid had dissolved for the most part, and a thick white suspension was obtained. Upon addition of further chloroform (3 mL), the suspension was slightly heated in a water bath, whereupon most of the solid dissolved to give a slightly cloudy solution. The filtrate obtained by pressing this solution through a syringe filter was placed in the freezer. A layer of crystals of the product emerged as a semi-transparent solid in the upper part of the clear solution. After 2 d, the solution was removed with a syringe, and the remaining solid was washed with *n*-hexane (ca. 2 × 1 mL). The product was then dried in a vacuum. Yield: 293 mg, 1.34 mmol, 28%. mp: 91–94 °C. ^13^C CP/MAS NMR (100.7 MHz, υ_rot_ = 10 kHz): δ_iso_ (ppm) = 4.8, 5.0, 5.4, 6.2 (Si*Me*_2_); 13.4 (H_2_N–CH_2_CH_2_*C*H_3_); 24.3, 24.6 (H_2_N–CH_2_*C*H_2_CH_3_); 31.3 (C*Me*_2_); 43.5, 44.1 (H_2_N–*C*H_2_CH_2_CH_3_); 56.2 (δ_iso(max)_ reported, asymmetric coupling pattern, *C*Me_2_); 183.9, 184.3 (*C*OO). ^29^Si CP/MAS NMR (79.5 MHz, υ_rot_ = 5 kHz): δ_iso(max)_ (ppm) = −73.4, −75.9. Anal. Calcd for C_9_H_22_N_2_O_2_Si (218.37 g/mol): C, 49.50; H, 10.18; N, 12.83. Found: C, 49.56; H, 10.17; N, 12.67%.

Compound (Aib)SiMe_2_(HIm)·(CHCl_3_): A small Schlenk tube, equipped with a magnetic stirring bar, was evacuated and then set under an argon atmosphere. Upon addition of the aminosilane (SiMe_2_(Im)_2_, 531 mg, 2.76 mmol) and α-amino isobutyric acid (H_2_Aib, 285 mg, 2.76 mmol), chloroform (2.5 mL) was added, and the mixture was stirred at room temperature for 5 h. During that time, the amino acid had dissolved for the most part, and a slightly cloudy solution remained (unreacted H_2_Aib). The solution was pressed through a syringe filter, and the clear filtrate was placed in the freezer. The product emerged as a semi-transparent solid in the upper part of the clear solution. After 2 d, the solution was removed with a syringe, and the remaining solid was washed with *n*-pentane (ca. 2 × 1 mL). The product was then dried in a vacuum. Yield: 273 mg, 0.79 mmol, 27%. ^13^C CP/MAS NMR (100.7 MHz, *υ*_rot_ = 10 kHz): *δ*_iso_ (ppm) = 5.2, 6.4 (Si*Me*_2_); 31.4 (C*Me*_2_); 56.6 (*δ*_iso(max)_ reported, asymmetric coupling pattern, *C*Me_2_); 80.0 (*C*HCl_3_); 118.5, 125.5, 135.2 (imidazole *C*H); 185.7 (*C*OO). ^29^Si CP/MAS NMR (79.5 MHz, *υ*_rot_ = 5 kHz): *δ*_iso(max)_ (ppm) = –71.5. Anal. Calcd for C_9_H_17_N_3_O_2_Si·CHCl_3_ (346.71 g/mol): C, 34.64; H, 5.24; N, 12.12. Found: C, 36.61; H, 6.06; N, 13.69%. We attribute the higher contents in C, H, N found to the loss of some solvent of crystallization (CHCl_3_) after sample preparation prior to combustion.

Compound (Aib)SiMe_2_(HPyr): In a small Schlenk tube, equipped with a magnetic stirring bar, α-amino isobutyric acid (H_2_Aib, 300 mg, 2.91 mmol) was evacuated and then set under an argon atmosphere. Upon addition of chloroform (2 mL), the mixture was cooled to 10 °C. To the cold mixture, the aminosilane (SiMe_2_(Pyr)_2_, 640 mg, 3.23 mmol) was added with a syringe, and stirring at 10 °C was continued for 5 h. During that time, the amino acid had dissolved for the most part, and a slightly cloudy solution remained (unreacted H_2_Aib). The solution was pressed through a syringe filter, and the clear filtrate was placed in a fridge (7 °C) overnight. The product emerged as a semi-transparent solid in the upper part of the clear solution. From the cold mixture, the solution was removed with a syringe, and the remaining solid was washed with *n*-hexane (ca. 2 × 1 mL). The product was then dried in a vacuum. Yield: 358 mg, 1.55 mmol, 53%. mp: 86–89 °C. ^13^C CP/MAS NMR (100.7 MHz, *υ*_rot_ = 10 kHz): *δ*_iso_ (ppm) = 1.0, 5.1 (Si*Me*_2_); 25.4, 26.9 (pyrrolidine β-*C*H_2_); 29.7, 31.6 (C*Me*_2_); 46.8, 49.1 (pyrrolidine α-*C*H_2_); 55.6 (*δ*_iso(max)_ reported, asymmetric coupling pattern, *C*Me_2_); 182.2 (*C*OO). ^29^Si CP/MAS NMR (79.5 MHz, *υ*_rot_ = 5 kHz): *δ*_iso(max)_ (ppm) = −71.6. Anal. Calcd for C_10_H_22_N_2_O_2_Si (230.38 g/mol): C, 52.13; H, 9.64; N, 12.16. Found: C, 52.09; H, 9.91; N, 12.20%. Storage of the filtrate in a freezer afforded some colorless crystalline needles of the solvate 2[(Aib)SiMe_2_(HPyr)]·3(CHCl_3_).

Compound (Aib)SiMeH(HPyr): In a small Schlenk tube, equipped with a magnetic stirring bar, α-amino isobutyric acid (H_2_Aib, 400 mg, 3.88 mmol) was evacuated and then set under an argon atmosphere. Upon addition of chloroform (2.5 mL), the mixture was cooled in an ice bath. To the cold mixture, the aminosilane (SiMeH(Pyr)_2_, 730 mg, 3.96 mmol) was added with a syringe, and stirring at 0 °C was continued for 5 h. During that time, a white dispersion was obtained, which was allowed to warm in a water bath to afford an almost clear solution. Upon filtration through a syringe filter, the filtrate was placed in the freezer. The product emerged as a semi-transparent solid in the upper part of the clear solution. After 1 d, the solution was removed with a syringe, and the remaining solid was washed with *n*-pentane (ca. 2 × 1 mL). The product was then dried in a vacuum. Yield: 421 mg, 1.95 mmol, 50%. mp: 92–95 °C. ^13^C CP/MAS NMR (100.7 MHz, *υ*_rot_ = 10 kHz): *δ*_iso_ (ppm) = 3.8 (Si*Me*); 24.3, 25.2 (pyrrolidine β-*C*H_2_); 30.1, 32.2 (C*Me*_2_); 47.0, 49.8 (pyrrolidine α-*C*H_2_); 56.1 (*δ*_iso(max)_ reported, asymmetric coupling pattern, *C*Me_2_); 183.7 (*C*OO). ^29^Si CP/MAS NMR (79.5 MHz, *υ*_rot_ = 5 kHz): *δ*_iso(max)_ (ppm) = −80.8. Anal. Calcd for C_9_H_20_N_2_O_2_Si (216.36 g/mol): C, 49.96; H, 9.34; N, 12.95. Found: C, 49.94; H, 9.54; N, 12.95%. Storage of a more dilute product solution in a freezer afforded crystals of the chloroform solvate 2[(Aib)SiMeH(HPyr)]·3(CHCl_3_).

Compound (Aib)SiMeVi(HPyr): In a small Schlenk tube, equipped with a magnetic stirring bar, α-amino isobutyric acid (H_2_Aib, 300 mg, 2.91 mmol) was evacuated and then set under an argon atmosphere. Upon addition of chloroform (2 mL), the mixture was cooled in an ice bath. To the cold mixture, the aminosilane (SiMeVi(Pyr)_2_, 690 mg, 3.28 mmol) was added with a syringe, and stirring at 0 °C was continued for 5 h. During that time, the amino acid had dissolved for the most part, and a slightly cloudy solution remained (unreacted H_2_Aib). The solution was pressed through a syringe filter, and the clear filtrate was placed in the freezer. The product emerged as a semi-transparent solid in the upper part of the clear solution. After 4 weeks, the solution was removed with a syringe, and the remaining solid was washed with *n*-hexane (ca. 2 × 1 mL). The product was then dried in a vacuum. Yield: 302 mg, 1.25 mmol, 43%. mp: 85–88 °C. ^13^C CP/MAS NMR (100.7 MHz, *υ*_rot_ = 10 kHz): *δ*_iso_ (ppm) = −0.8, −0.4, 3.8 (Si*Me*); 26.8, 27.7 (pyrrolidine β-*C*H_2_); 29.7, 30.3, 31.4, 32.2 (C*Me*_2_); 47.8 (pyrrolidine α-*C*H_2_); 56.0 (*δ*_iso(max)_ reported, asymmetric coupling pattern, *C*Me_2_); 128.8, 133.1, 138.4, 140.7, 142.6, 145.2 (*C*H=*C*H_2_); 181.6, 183.2, 183.7 (*C*OO). ^29^Si CP/MAS NMR (79.5 MHz, *υ*_rot_ = 5 kHz): *δ*_iso(max)_ (ppm) = −81.2. Anal. Calcd for C_11_H_22_N_2_O_2_Si (242.39 g/mol): C, 54.50; H, 9.17; N, 11.56. Found: C, 54.62; H, 9.12; N, 11.07%.

Compound (Aib)SiEt_2_(HPyr): In a small Schlenk tube, equipped with a magnetic stirring bar, α-amino isobutyric acid (H_2_Aib, 302 mg, 2.93 mmol) was evacuated and then set under an argon atmosphere. Upon addition of chloroform (2 mL), the mixture was cooled in an ice bath. To the cold mixture, the aminosilane (SiEt_2_(Pyr)_2_, 720 mg, 3.18 mmol) was added with a syringe, and stirring at 0 °C was continued for 5 h. During that time, the amino acid had dissolved for the most part, and a slightly cloudy solution remained (unreacted H_2_Aib). The solution was pressed through a syringe filter, and the clear filtrate was placed in the freezer. The product emerged as a semi-transparent solid in the upper part of the clear solution. After 4 weeks, the solution was removed with a syringe, and the remaining solid was washed with *n*-hexane (ca. 2 × 1 mL). The product was then dried in a vacuum. Yield: 283 mg, 1.10 mmol, 37%. mp: 73–76 °C. ^13^C CP/MAS NMR (100.7 MHz, *υ*_rot_ = 5 kHz): *δ*_iso_ (ppm) = 7.5, 8.8, 10.1, 11.6 (Si*(C*H_2_*C*H_3_)_2_); 25.6, 26.3 (pyrrolidine β-*C*H_2_); 30.4, 32.1 (C*Me*_2_); 47.5, 49.2 (pyrrolidine α-*C*H_2_); 55.8 (*δ*_iso(max)_ reported, asymmetric coupling pattern, *C*Me_2_); 183.4 (*C*OO). ^29^Si CP/MAS NMR (79.5 MHz, *υ*_rot_ = 5 kHz): *δ*_iso(max)_ (ppm) = –62.9. Anal. Calcd for C_12_H_26_N_2_O_2_Si (258.44 g/mol): C, 55.77; H, 10.16; N, 10.84. Found: C, 55.28; H, 10.30; N, 10.99%.

Compound [*t*BuNH_3_]^+^[(Aib)_2_SiMe]^−^: In a small Schlenk tube, equipped with a magnetic stirring bar, α-amino isobutyric acid (H_2_Aib, 300 mg, 2.91 mmol) was evacuated and then set under an argon atmosphere. Upon addition of chloroform (2 mL), the aminosilane SiMe_2_(NH*t*Bu)_2_ (2.48 g, 12.3 mmol) was added with a syringe, and stirring at room temperature was continued for 24 h. During that time, the amino acid had dissolved, and a clear solution was obtained. Thereafter, more α-amino isobutyric acid (H_2_Aib, 900 mg, 8.73 mmol) was added, and the mixture was stirred further at room temperature. The subsequently added amino acid had completely dissolved after 24 h. After 5 more days, a thick white suspension had formed. The solid was filtered off and washed with a small amount of chloroform (ca. 2 × 1 mL, 1 × 0.5 mL) and briefly dried in a vacuum. Yield: 445 mg, 1.39 mmol, 24% (with respect to the composition [*t*BuNH_3_]^+^[(Aib)_2_SiMe]^−^ with M = 319.48 g/mol according to NMR spectroscopy; *t*BuNH_2_ and CHCl_3_ of the crystal structure are removed while drying). mp: decomposition at 224 °C. ^1^H NMR (400.1 MHz, DMSO-d6, TMS): *δ* (ppm) = −0.09 (s, 3H, SiMe); 1.10 (s, 6H, AibMe_2_); 1.12 (s, 6H, AibMe_2_); 1.23 (s, 9H, *t*Bu); 2–8 (broad, 5H, NH and NH_3_). ^13^C NMR (100.6 MHz, DMSO-d6, TMS): *δ* (ppm) = 4.9 (Si*Me*); 27.8 (C*Me*_3_); 30.2, 30.5 (Aib*Me*_2_); 50.4 (*C*Me_3_); 54.7 (Aib *αC*); 180.1 (*C*OO). ^29^Si NMR (79.5 MHz, DMSO-d6, TMS): *δ* (ppm) = −89.9. Anal. Calcd for C_13_H_29_N_3_O_4_Si (319.48 g/mol): C, 48.87; H, 9.15; N, 13.15. Found: C, 48.88; H, 9.16; N, 13.15%.

## 4. Conclusions

Our investigation revealed the capability of amino acid-derived diorganosilicon compounds to coordinate primary and secondary amines with the formation of hypercoordinate Si complexes. For other primary and secondary amines, this has previously been achieved with the aid of highly Lewis acidic Si centers (cf. compounds **II** [9] and **III** [10]). When amine adduct formation is sterically hampered (in case of *t*BuNH_2_), Si–CH_3_ bond cleavage by a second amino acid molecule may occur. Both observations indicate interesting Lewis acidity features of α-amino acid-derived diorganosilicon compounds and motivate further investigations of, e.g., adduct formation with other Lewis bases and their utilization in syntheses. An initial check of the fluoride ion affinity (FIA, which is a kind of measure for hard Lewis acidity) of the [(Aib)SiMe_2_] moiety [49,50] predicted FIA values of 300 and 121 kJ·mol^−1^ for gas phase and in solution, respectively, which is noticeably pronounced FIA with respect to the 2-aminoethanol-derived silacycle [(O–CH_2_CH_2_–NH)SiMe_2_] (corresponding FIA values: 210 and 58 kJ·mol^−1^) or the fluorosilane SiMe_2_F_2_ (corresponding FIA values: 221 and 86 kJ·mol^−1^).

## Figures and Tables

**Table 1:** Selected bond lengths (Å) and angles (deg.) as well as the geometry index τ_5_ [15] of the Si-coordination spheres of the previously published compound (Aib)SiMe_2_(NMI) [13] and the Lewis–base adducts obtained in the current work. (As the Si coordination spheres may, in addition to N1 and C5, feature one equatorial atom with an inconsistent label, this third equatorial atom is denoted as *A*3.) For entries 3 ((Aib)SiMe_2_(H_2_N*n*Pr)), 7 (chloroform solvate of (Aib)SiMeH(HPyr)), and 8 ((Aib)SiMeVi(HPyr)), which feature two crystallographically independent adduct molecules in the asymmetric unit, the second line lists the corresponding bond lengths and angles of the second molecule irrespective of the atom labels.

Compound	Si1–O1	Si1–N1	Si1–N2	O1–Si1–N2	N1–Si1–C5	N1–Si1–*A*3	C5–Si1–*A*3	τ_5_
(Aib)SiMe_2_(NMI) ^1^	1.852(2)	1.713(2)	2.036(2)	172.3(2)	123.4(2)	122.7(2)	113.9(2)	0.82
(Aib)SiMe_2_(HIm) ^2^	1.867(2)	1.720(2)	2.034(2)	172.2(1)	121.9(1)	124.1(1)	114.0(1)	0.80
(Aib)SiMe_2_(H_2_N*n*Pr)	1.877(3)	1.717(4)	2.031(4)	172.5(2)	120.6(2)	123.9(2)	115.4(2)	0.81
	1.882(3)	1.711(4)	2.011(4)	172.2(2)	121.5(2)	125.3(2)	113.2(2)	0.78
(Aib)SiMe_2_(HPyr)	1.869(1)	1.718(2)	2.031(2)	172.5(1)	120.9(1)	123.3(1)	115.8(1)	0.82
(Aib)SiMe_2_(HPyr) ^3^	1.877(3)	1.715(4)	2.034(4)	172.1(2)	121.5(2)	122.5(2)	116.0(2)	0.83
(Aib)SiMeH(HPyr)	1.840(2)	1.708(2)	1.986(2)	170.1(1)	120.8(2)	124.6(8)	114.6(8)	0.76
(Aib)SiMeH(HPyr) ^4^	1.845(2)	1.697(2)	1.996(2)	170.0(1)	119.8(1)	123.2(8)	116.9(8)	0.78
	1.861(2)	1.700(2)	1.989(2)	170.9(1)	120.9(2)	124.1(8)	115.0(8)	0.78
(Aib)SiMeVi(HPyr) ^5^	1.858(2)	1.712(2)	2.014(2)	173.1(1)	124.6(2)	121.3(2)	114.2(2)	0.81
	1.852(2)	1.702(2)	2.029(2)	173.8(1)	125.8(4)	119.1(4)	115.1(3)	0.80
(Aib)SiEt_2_(HPyr)	1.871(2)	1.713(2)	2.081(2)	171.3(1)	123.2(1)	122.4(1)	114.4(1)	0.80

^1^ Data from the crystal structure of the chloroform solvate (Aib)SiMe_2_(NMI)·(CHCl_3_) [13]. ^2^ Data from the crystal structure of the chloroform solvate (Aib)SiMe_2_(HIm)·(CHCl_3_). ^3^ Data from the crystal structure of the chloroform solvate 2[(Aib)SiMe_2_(HPyr)]·3(CHCl_3_). ^4^ Data from the crystal structure of the chloroform solvate 2[(Aib)SiMeH(HPyr)]·3(CHCl_3_). ^5^ The SiMeVi moieties of the two independent molecules are disordered by means of site exchange. The data reported in this table correspond to the parts with predominant site occupancies, which are 0.792(5) for molecule 1 and 0.558(4) for molecule 2.

**Table 2:** *D*⋯O distances *d*(*D*⋯O) (Å) and *D*–H⋯O angles (deg.) of the H-bond donor atom (*D*) and H-bond acceptor O atom in hydrogen bonds of the carboxylate groups of the Lewis-base adducts investigated in the current work. Note: these *D*–H⋯O angles should be interpreted with care. As the H sites in X-ray diffraction analyses represent the location of the H-associated electron density peak (not the site of the H atom), the *D*–H⋯O angles are reported without decimal places and without s.u.s and just serve as a rough estimate of that angle.

Compound	C⋯O_carbonyl_	*d*(*D*⋯O) *D*–H⋯O	N⋯O_carbonyl_	*d*(*D*⋯O) *D*–H⋯O	N⋯O_carboxylato_	*d*(*D*⋯O) *D*–H⋯O	Symmetry Operations
(Aib)SiMe_2_(HIm) ^1^	C10⋯O2	2.940(2) 128	N3^a^⋯O2	2.755(2) 171	-	-	^a^ x, 1.5 − y, −0.5 + z
(Aib)SiMe_2_(H_2_N*n*Pr) molecule 1	-	-	N4^e^⋯O2 N1^f^⋯O2 N2^f^⋯O2	2.980(6) 146 3.247(5) 158 3.177(6) 153	N2^f^⋯O1	3.279(5) 147	^e^ x, 1 + y, z; ^f^ −0.5 + x, 2 − y, −0.5 + z
(Aib)SiMe_2_(H_2_N*n*Pr) molecule 2	-	-	N2⋯O4 N4^g^⋯O4	3.081(6) 177 2.973(5) 157	N3^g^⋯O3	3.272(5) 174	^g^ 0.5 + x, 1 − y, 0.5 + z
(Aib)SiMe_2_(HPyr)	-	-	N1^c^⋯O2	3.058(2) 163	N2^c^⋯O1	3.085(2) 154	^c^ −0.5 + x, 1.5 − y, −0.5 + z
(Aib)SiMe_2_(HPyr) ^2^	C11⋯O2 ^4^	3.052(11) 172	N2^b^⋯O2	2.918(4) 175	-	-	^b^ 0.5 − x, y, 0.5 + z
(Aib)SiMeH(HPyr)	-	-	N2^d^⋯O2	2.932(2) 169	N1^d^⋯O1	3.193(2) 172	^d^ x, 1 − y, 0.5 + z
(Aib)SiMeH(HPyr) ^3^ molecule 1	C20⋯O2 ^5^	3.069(8) 164	N4^h^⋯O2	2.887(2) 164	N3^h^⋯O1	3.214(2) 172	^h^ x, −1 + y, z
(Aib)SiMeH(HPyr) ^3^ molecule 2	C19^i^⋯O4 ^6^	3.114(6) 157	N2⋯O4	2.918(2) 167	N1⋯O3	3.223(2) 169	^i^ −1 + x, y, z
(Aib)SiMeVi(HPyr) molecule 1	-	-	N3^j^⋯O2	3.076(2) 169	N4^j^⋯O1	3.003(2) 165	^j^ x, 1 + y, z
(Aib)SiMeVi(HPyr) molecule 2	-	-	N1⋯O4	3.173(2) 154	N2⋯O3	3.307(2) 144	-
(Aib)SiEt_2_(HPyr)	-	-	N1^k^⋯O2	3.013(2) 170	N2^k^⋯O1	3.178(2) 166	^k^ 0.5 + x, 0.5 − y, 0.5 + z

^1^ Data from the crystal structure of the chloroform solvate (Aib)SiMe_2_(HIm)·(CHCl_3_). ^2^ Data from the crystal structure of the chloroform solvate 2[(Aib)SiMe_2_(HPyr)]·3(CHCl_3_). ^3^ Data from the crystal structure of the chloroform solvate 2[(Aib)SiMeH(HPyr)]·3(CHCl_3_). ^4^ The chloroform molecule was refined disordered over 3 positions, the C atom coordinates of the site with predominant occupancy, s.o.f. 0.475(3), were employed. ^5^ The chloroform molecule was refined disordered over 3 positions; the C atom coordinates of the site with predominant occupancy, s.o.f. 0.405(3), were employed. ^6^ The chloroform molecule was refined disordered over 3 positions; the C atom coordinates of the site with predominant occupancy, s.o.f. 0.496(3), were employed.

**Figure A1:**
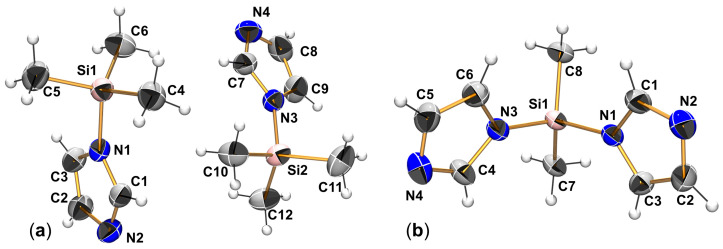
Crystallographically determined molecular structures of silanes SiMe_3_(Im) (**a**) and SiMe_2_(Im)_2_ (**b**) with thermal ellipsoids at the 50% level and labels of non-hydrogen atoms. In (**a**), the two crystallographically independent molecules of SiMe_3_(Im) are shown in their relative position to one another in the asymmetric unit.

**Figure A2:**
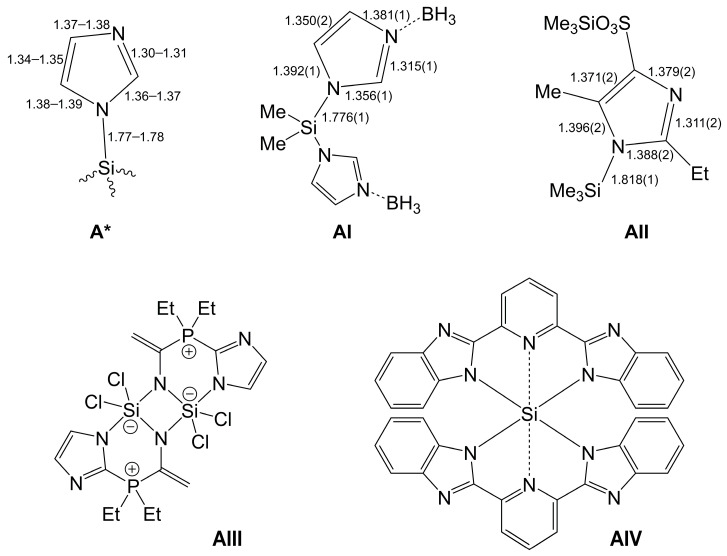
Further examples of imidazolyl silane derivatives (**AI** and **AII**) with bond lengths (Å) of their imidazolyl groups complemented by the corresponding bond lengths pattern (A*) encountered with the imidazolyl silanes SiMe_3_(Im) and SiMe_2_(Im)_2_ (cf. Table A6), as well as hypercoordinate Si complexes with imidazolyl and benzimidazolyl groups in their ligand backbones (**AIII** and **AIV**, respectively).

## Data Availability

CCDC 2474608 ([*t*BuNH_3_][(Aib)_2_SiMe]·(CHCl_3_)·(*t*BuNH_2_)), 2474609 ((Aib)SiMe_2_(HPyr)), 2474610 ((Aib)SiMeH(HPyr)), 2474611 (SiMe_2_(Im)_2_), 2474612 ((Aib)SiMe_2_(H_2_N*n*Pr)), 2474613 (2[(Aib)SiMe_2_(HPyr)]·3(CHCl_3_)), 2474614 ((Aib)SiMeVi(HPyr)), 2474615 (2[(Aib)SiMeH(HPyr)]·3(CHCl_3_)), 2474616 ((Aib)SiMe_2_(HIm)·(CHCl_3_)), 2474617 (SiMe_3_(Im)), and 2474618 ((Aib)SiEt_2_(HPyr)) contain the supplementary crystal data for this article. These data can be obtained free of charge from the Cambridge Crystallographic Data Centre via https://www.ccdc.cam.ac.uk/structures/ (accessed on 21 July 2025).

## Supplementary Materials

The following supporting information can be downloaded at: https://www.mdpi.com/article/10.3390/molecules30173501/s1, ^13^C{^1^H} and ^29^Si{^1^H} CP/MAS NMR spectra (Figure S1: ^13^C{^1^H} CP/MAS NMR spectrum of (Aib)SiMe_2_(HIm)·CHCl_3_; Figure S2: ^29^Si{^1^H} CP/MAS NMR spectrum of (Aib)SiMe_2_(HIm)·CHCl_3_; Figure S3: ^13^C{^1^H} CP/MAS NMR spectrum of (Aib)SiMe_2_(NH_2_*n*Pr); Figure S4: magnification of the upfield section of the ^13^C{^1^H} CP/MAS NMR spectrum of (Aib)SiMe_2_(NH_2_*n*Pr); Figure S5: ^29^Si{^1^H} CP/MAS NMR spectrum of (Aib)SiMe_2_(NH_2_*n*Pr); Figure S6: ^13^C{^1^H} CP/MAS NMR spectrum of (Aib)SiMe_2_(HPyr); Figure S7: ^29^Si{^1^H} CP/MAS NMR spectrum of (Aib)SiMe_2_(HPyr); Figure S8: ^13^C{^1^H} CP/MAS NMR spectrum of (Aib)SiMeH(HPyr); Figure S9: ^29^Si{^1^H} CP/MAS NMR spectrum of (Aib)SiMeH(HPyr); Figure S10: ^13^C{^1^H} CP/MAS NMR spectrum of (Aib)SiMeVi(HPyr); Figure S11: magnification of the upfield section of the ^13^C{^1^H} CP/MAS NMR spectrum of (Aib)SiMeVi(HPyr); Figure S12: magnification of the downfield section of the ^13^C{^1^H} CP/MAS NMR spectrum of (Aib)SiMeVi(HPyr); Figure S13: ^29^Si{^1^H} CP/MAS NMR spectrum of (Aib)SiMeVi(HPyr); Figure S14: ^13^C{^1^H} CP/MAS NMR spectrum of (Aib)SiEt_2_(HPyr); Figure S15: ^29^Si{^1^H} CP/MAS NMR spectrum of (Aib)SiEt_2_(HPyr).) ^1^H, ^13^C{^1^H} and ^29^Si{^1^H} solution NMR spectra; Figure S16: ^1^H NMR spectrum (DMSO-d6) of [*t*BuNH_3_][(Aib)_2_SiMe]; Figure S17: ^13^C{^1^H} NMR spectrum (DMSO-d6) of [*t*BuNH_3_][(Aib)_2_SiMe]; Figure S18: ^29^Si{^1^H} NMR spectrum (DMSO-d6) of [*t*BuNH_3_][(Aib)_2_SiMe]) IR (ATR) spectra; Figure S19: IR (ATR) spectrum of (Aib)SiMe_2_(HIm)·(CHCl_3_); Figure S20: IR (ATR) spectrum of (Aib)SiMe_2_(NH_2_*n*Pr); Figure S21: IR (ATR) spectrum of (Aib)SiMe_2_(HPyr); Figure S22: IR (ATR) spectrum of (Aib)SiMeH(HPyr); Figure S23: IR (ATR) spectrum of (Aib)SiMeVi(HPyr); Figure S24: IR (ATR) spectrum of (Aib)SiEt_2_(HPyr); Figure S25: IR (ATR) spectrum of [*t*BuNH_3_][(Aib)_2_SiMe]. Details of the synthesis of SiMe_3_(Im) and synthesis of SiMe_2_(Im)_2_; Figure S26: Thermal ellipsoid plot of part of the molecule of (Aib)SiMe_2_(HPyr), i.e., the pyrrolidine ligand at the Si atom, before refinement of the pyrrolidine disorder and showing the location of the highest residual electron density peak (left) and upon refinement of the disorder (right). C-bound H atoms were omitted for clarity; Figure S27: space-fill representation of one of the two crystallographically independent molecules of the crystal structure of (Aib)SiMe_2_(H_2_N*n*Pr) in two different perspectives, related to one another by a 180° rotation about the in-plane horizontal axis. Left: view of the NH_2_ group attached to the Si atom. Right: view of the α-H atoms of the propylamine moiety; Figure S28: ^29^Si CP/MAS NMR spectrum of (Aib)SiMe_2_(HPyr) (*ν*_rot_ = 3 kHz). Bottom, blue: experimental spectrum upon smoothing; top, red: modeled spectrum using the CSA tensor principal values obtained by DMFIT analysis; Figure S29: ^29^Si CP/MAS NMR spectrum of (Aib)SiMeH(HPyr) (*ν*_rot_ = 3 kHz). Bottom, blue: experimental spectrum upon smoothing; top, red: modeled spectrum using the CSA tensor principal values obtained by DMFIT analysis; Figure S30: ^29^Si CP/MAS NMR spectrum of (Aib)SiMe_2_(H_2_N*n*Pr) (*ν*_rot_ = 3 kHz). Bottom, blue: experimental spectrum upon smoothing; top, red: modeled spectrum using the CSA tensor principal values obtained by DMFIT analysis; Figure S31: ^29^Si CP/MAS NMR spectrum of (Aib)SiEt_2_(HPyr) (*ν*_rot_ = 3 kHz). Bottom, blue: experimental spectrum upon smoothing; top, red: modeled spectrum using the CSA tensor principal values obtained by DMFIT analysis; Figure S32: ^29^Si CP/MAS NMR spectrum of (Aib)SiMeVi(HPyr) (*ν*_rot_ = 2 kHz). Bottom, blue: experimental spectrum upon smoothing; top, red: modeled spectrum using the CSA tensor principal values obtained by DMFIT analysis; Figure S33: ^29^Si CP/MAS NMR spectrum of (Aib)SiMe_2_(HIm)·(CHCl_3_) (*ν*_rot_ = 3 kHz). Bottom, blue: experimental spectrum upon smoothing; top, red: modeled spectrum using the CSA tensor principal values obtained by DMFIT analysis; Table S1: comparison of selected parameters before and after refinement of the disorder in the pyrrolidine ring of (Aib)SiMe_2_(HPyr); Table S2: results of ^29^Si CSA tensor analyses of compounds (Aib)SiMe_2_(HPyr), (Aib)SiMeH(HPyr), (Aib)SiMe_2_(H_2_N*n*Pr), (Aib)SiEt_2_(HPyr), (Aib)SiMeVi(HPyr), and (Aib)SiMe_2_(HIm)·(CHCl_3_).

## Appendix A

**Table A1:** Crystallographic data from data collection and refinement for SiMe_3_(Im) and SiMe_2_(Im)_2_.

Parameter	SiMe_3_(Im) ^1^	SiMe_2_(Im)_2_
Formula	C_6_H_12_N_2_Si	C_8_H_12_N_4_Si
*M*_r_	140.27	192.31
*T*(K)	200(2)	180(2)
*λ*(Å)	0.71073	0.71073
Crystal system	monoclinic	orthorhombic
Space group	*P*2_1_/*c*	*Pccn*
*a*(Å)	12.7551(4)	21.6042(8)
*b*(Å)	10.6258(2)	7.8214(2)
*c*(Å)	13.0791(3)	12.0118(3)
*β*(°)	106.961(2)	90
*V*(Å^3^)	1695.55(8)	2029.70(10)
*Z*	8	8
*ρ*_calc_(g·cm^−1^)	1.10	1.26
*μ*_MoKα_ (mm^−1^)	0.2	0.2
*F*(000)	608	816
*θ*_max_(°), *R*_int_	26.0, 0.1085 ^1^	26.0, 0.0854
Completeness	99.9%	99.9%
Reflns collected	22,510	21,792
Reflns unique	3323	1999
Restraints	0	0
Parameters	170	120
GoF	1.040	0.995
*R*1, *wR*2 [*I* > 2*σ*(*I*)]	0.0401, 0.1098	0.0424, 0.1142
*R*1, *wR*2 (all data)	0.0426, 0.1117	0.0562, 0.1215
Largest peak/hole (e·Å^−3^)	0.22, −0.32	0.40, −0.30

^1^ This compound was crystallized in a glass capillary (1 mm diameter) on the goniometer under the nitrogen cold stream. The crystalline part used for data collection contained multiple domains (five individual domains could be indexed in the diffraction patterns), but they did not adhere to systematic twin laws. The data set of the strongest domain could be integrated as a single-crystal data set, as it did not suffer from greater overlap with reflections of other domains. In spite of the satisfactory structure refinement as a “single-crystal”, we attribute the large *R*_int_ to effects of some non-systematic overlap with data of other domains.

**Table A2:** Crystallographic data from data collection and refinement for (Aib)SiMe_2_(HIm)·(CHCl_3_) and 2[(Aib)SiMe_2_(HPyr)]·3(CHCl_3_).

Parameter	(Aib)SiMe_2_(HIm)·(CHCl_3_)	2[(Aib)SiMe_2_(HPyr)]·3(CHCl_3_) ^1^
Formula	C_10_H_18_Cl_3_N_3_O_2_Si	C_23_H_47_Cl_9_N_4_O_4_Si_2_
*M*_r_	346.71	818.87
*T*(K)	180(2)	180(2)
*λ*(Å)	0.71073	0.71073
Crystal system	orthorhombic	orthorhombic
Space group	*Pbca*	*Pccn*
*a*(Å)	10.9384(4)	15.6692(14)
*b*(Å)	16.7355(6)	21.4914(18)
*c*(Å)	17.7731(9)	11.6660(9)
*V*(Å^3^)	3253.5(2)	3928.6(6)
*Z*	8	4
*ρ*_calc_(g·cm^−1^)	1.42	1.38
*μ*_MoKα_ (mm^−1^)	0.6	0.7
*F*(000)	1440	1704
*θ*_max_(°), *R*_int_	27.0, 0.0533	23.5, 0.1998 ^1^
Completeness	100%	99.9%
Reflns collected	33,809	25,213
Reflns unique	3541	2905
Restraints	0	94 ^2^
Parameters	184	276
GoF	1.049	1.019
*R*1, *wR*2 [*I* > 2*σ*(*I*)]	0.0293, 0.0677	0.0493, 0.0804
*R*1, *wR*2 (all data)	0.0459, 0.0729	0.1359, 0.1077 ^1^
Largest peak/hole (e·Å^−3^)	0.27, −0.23	0.22, −0.26

^1^ These crystals had very poor diffraction power. Within the range of data collection, only 50.4% of the data were observed [*I* > 2*σ*(*I*)]. In the upper *θ* increment between 22.0°–23.5°, only 16.0% of the data were observed with *I* > 2*σ*(*I*). Beyond the upper theta limit used in the refinement, essentially no useful data were available, i.e., in the *θ* increment between 23.5°–25.0°, only 7.8% of the data were observed with *I* > 2*σ*(*I*). The poor signal/noise of the data set is the major reason for the large values of *R*_int_ and *R*1(all data). ^2^ Restraints were used in the refinement of disordered groups in this crystal structure, i.e., one chloroform molecule as well as the pyrrolidine ligand suffer disorder. The former was refined in three positions (with site occupancies 0.330(3), 0.195(3), 0.475(3)). In the latter, the C–C bond of the pyrrolidine 3,4-positions was disordered in a cross-wise manner and was refined over two sites, with site occupancies 0.773(14), 0.227(14).

**Table A3:** Crystallographic data from data collection and refinement for (Aib)SiMe_2_(HPyr) and (Aib)SiMe_2_(H_2_N*n*Pr).

Parameter	(Aib)SiMe_2_(HPyr)	(Aib)SiMe_2_(H_2_N*n*Pr)
Formula	C_10_H_22_N_2_O_2_Si	C_9_H_22_N_2_O_2_Si
*M*_r_	230.38	218.37
*T*(K)	140(2)	140(2)
*λ*(Å)	0.71073	0.71073
Crystal system	monoclinic	monoclinic
Space group	*P*2_1_/*n*	*Pn*
*a*(Å)	10.1535(3)	9.9795(4)
*b*(Å)	13.9171(3)	13.4515(8)
*c*(Å)	10.3250(3)	10.2116(4)
*β*(°)	110.254(2)	111.317(3)
*V*(Å^3^)	1368.78(7)	1277.01(11)
*Z*	4	4
*ρ*_calc_(g·cm^−1^)	1.12	1.14
*μ*_MoKα_ (mm^−1^)	0.2	0.2
*F*(000)	504	480
*θ*_max_(°), *R*_int_	28.0, 0.0515	26.0, 0.1060
Completeness	100%	100%
Reflns collected	24,955	22,161
Reflns unique	3304	4861
Restraints	7 ^1^	4 ^2^
Parameters	156	289
GoF	1.049	0.993
*R*1, *wR*2 [*I* > 2*σ*(*I*)]	0.0344, 0.0877	0.0474, 0.0920
*R*1, *wR*2 (all data)	0.0467, 0.0924	0.0855, 0.1030
*χ*_Flack_	n/a	0.33(15)
Largest peak/hole (e·Å^−3^)	0.21, −0.21	0.18, −0.24

^1^ Restraints were used in the refinement of a disorder in the pyrrolidine ligand. Its C–C bond of the pyrrolidine 3,4-positions was disordered in a cross-wise manner and was refined over two sites, with site occupancies 0.88(1), 0.12(1). ^2^ Restraints were used in the refinement of a disorder in the propyl group of one of the two crystallographically independent molecules. Its terminal C–C bond was disordered in a cross-wise manner and was refined over two sites, with site occupancies 0.865(7), 0.135(7).

**Table A4:** Crystallographic data from data collection and refinement for (Aib)SiEt_2_(HPyr), (Aib)SiMeVi(HPyr), and (Aib)SiMeH(HPyr).

Parameter	(Aib)SiEt_2_(HPyr)	(Aib)SiMeVi(HPyr)	(Aib)SiMeH(HPyr)
Formula	C_12_H_26_N_2_O_2_Si	C_11_H_22_N_2_O_2_Si	C_9_H_20_N_2_O_2_Si
*M*_r_	258.44	242.39	216.36
*T*(K)	180(2)	180(2)	160(2)
*λ*(Å)	0.71073	0.71073	0.71073
Crystal system	monoclinic	monoclinic	monoclinic
Space group	*P*2_1_/*n*	*P*2_1_/*c*	*C*2/*c*
*a*(Å)	10.1751(4)	12.9780(4)	12.9491(8)
*b*(Å)	14.8904(6)	12.1486(3)	17.6134(8)
*c*(Å)	10.5661(3)	17.9866(6)	11.0312(5)
*β*(°)	109.834(2)	102.055(3)	97.538(4)
*V*(Å^3^)	1505.92(10)	2773.31(15)	2494.2(2)
*Z*	4	8	8
*ρ*_calc_(g·cm^−1^)	1.14	1.16	1.15
*μ*_MoKα_ (mm^−1^)	0.2	0.2	0.2
*F*(000)	568	1056	944
*θ*_max_(°), *R*_int_	28.0, 0.0524	26.0, 0.0443	25.0, 0.1084
Completeness	100%	100%	99.9%
Reflns collected	13,638	26,472	12,357
Reflns unique	3627	5428	2186
Restraints	21 ^1^	31 ^2^	0
Parameters	182	393	139
GoF	1.021	1.041	0.971
*R*1, *wR*2 [*I* > 2*σ*(*I*)]	0.0405, 0.0972	0.0412, 0.0945	0.0446, 0.1023
*R*1, *wR*2 (all data)	0.0617, 0.1061	0.0620, 0.1031	0.0893, 0.1163
Largest peak/hole (e·Å^−3^)	0.32, −0.19	0.25, −0.21	0.19, −0.23

^1^ Restraints were used in the refinement of a disorder in the pyrrolidine ligand. Its C–C bond of the pyrrolidine 3,4-positions was disordered in a cross-wise manner and was refined over two sites, with site occupancies 0.858(6), 0.142(6). ^2^ Restraints were used in the refinement of two sets of coupled disorders in this structure, which consist of alternative Me/Vi site occupancy of one molecule and a disorder in the pyrrolidine ligand (C–C bond of the pyrrolidine 3,4-positions was disordered in a cross-wise manner) of an adjacent molecule, which corresponds to the second molecule in the asymmetric unit. The set of SiMe/Vi(@Si1) and HPyr(@Si2) disorders was refined with site occupancies 0.792(5), 0.208(5), and the set of SiMe/Vi(@Si2) and HPyr(@Si1) disorders was refined with site occupancies 0.558(4), 0.442(4).

**Table A5:** Crystallographic data from data collection and refinement for 2[(Aib)SiMeH(HPyr)]·3(CHCl_3_) and [*t*BuNH_3_][(Aib)_2_SiMe]·(CHCl_3_)·(*t*BuNH_2_).

Parameter	2[(Aib)SiMeH(HPyr)] ·3(CHCl_3_) ^1^	[*t*BuNH_3_][(Aib)_2_SiMe] ·(CHCl_3_)·(*t*BuNH_2_)
Formula	C_21_H_43_Cl_9_N_4_O_4_Si_2_	C_18_H_41_Cl_3_N_4_O_4_Si
*M*_r_	790.82	511.99
*T*(K)	230(2) ^2^	100(2)
*λ*(Å)	0.71073	1.54184
Crystal system	triclinic	triclinic
Space group	$P\bar{1}$	$P\bar{1}$
*a*(Å)	10.3734(3)	10.4171(1)
*b*(Å)	11.2006(4)	11.9432(1)
*c*(Å)	17.7795(6)	12.8295(1)
*α*(°)	105.412(3)	96.129(1)
*β*(°)	93.183(3)	106.033(1)
*γ*(°)	98.289(3)	105.231(1)
*V*(Å^3^)	1961.12(12)	1452.25(2)
*Z*	2	2
*ρ*_calc_(g·cm^−1^)	1.34	1.17
*μ*_MoKα_ (mm^−1^)	0.7	3.5
*F*(000)	820	548
*θ*_max_(°), *R*_int_	26.0, 0.0648	74.5, 0.0480
Completeness	100%	99.7%
Reflns collected	33,664	113,590
Reflns unique	7701	5891
Restraints	173 ^3^	0
Parameters	482	310
GoF	1.016	1.040
*R*1, *wR*2 [*I* > 2*σ*(*I*)]	0.0446, 0.1360	0.0379, 0.0993
*R*1, *wR*2 (all data)	0.0656, 0.1443	0.0390, 0.1002
Largest peak/hole (e·Å^−3^)	0.19, −0.25	0.70, −0.49

^1^ The asymmetric unit of this crystal structure consists of two molecules of (Aib)SiMeH(HPyr) and three molecules of solvent of crystallization (i.e., chloroform) in a severely disordered manner. Only two of them were refined; the third one was omitted from refinement, and its contribution to the diffraction data was accounted for by using SQUEEZE as implemented in PLATON [51,52,53]. This procedure identified, per unit cell, the solvent-accessible volume of 323 Å^3^ and contributions of 117 electrons therein. This number of electrons corresponds well to the 116 electrons of two CHCl_3_ molecules per unit cell omitted from the refinement. ^2^ Upon cooling to 200 K, the crystals collapsed. Therefore, the data set was collected at a higher temperature. ^3^ Restraints were used in the refinement of disordered groups in this crystal structure, i.e., two chloroform molecules as well as the pyrrolidine ligand of one of the two crystallographically independent molecules of (Aib)SiMeH(HPyr). The former were refined in three positions each (with site occupancies 0.496(3), 0.434(3), 0.071(3) and 0.289(3), 0.405(3), 0.306(3)). In the latter, the C–C bond of the pyrrolidine 3,4-positions was disordered in a cross-wise manner and was refined over two sites, with site occupancies 0.78(1), 0.22(1).

## Appendix B

In the course of preparing silane SiMe_2_(Im)_2_ and its precursor SiMe_3_(Im), we obtained the former as a crystalline solid, the raw product containing crystals suitable for single-crystal X-ray diffraction analysis. The latter, a liquid at room temperature, could be crystallized in a capillary in the nitrogen cold stream on the diffractometer (by following the protocol we used for crystallizing liquid chlorosilanes [41]). For selected parameters of data collection, unit cell, and structure refinement, see Appendix A, Table A1. The molecular structures of SiMe_3_(Im) and SiMe_2_(Im)_2_ are shown in Figure A1, and selected bond lengths and angles are listed in Table A6.

**Table A6:** Selected bond lengths (Å) and angles (deg.) in compounds SiMe_3_(Im) and SiMe_2_(Im)_2_ in their crystal structures.

	SiMe_3_(Im)		SiMe_2_(Im)_2_
Si1–N1, Si2–N3	1.778(2), 1.780(2)	Si1–N1	1.770(2)
Si1–C4, Si2–C10	1.846(2), 1.846(2)	Si1–N3	1.753(2)
Si1–C5, Si2–C11	1.849(2), 1.845(2)	Si1–C7	1.833(2)
Si1–C6, Si2–C12	1.843(2), 1.854(2)	Si1–C8	1.826(2)
N1–C1, N3–C7	1.359(2), 1.361(2)	N1–C1, N3–C4	1.372(2), 1.368(2)
N2–C1, N4–C7	1.310(2), 1.305(2)	N2–C1, N4–C4	1.308(3), 1.307(2)
N1–C3, N3–C9	1.382(2), 1.383(2)	N1–C3, N3–C6	1.380(2), 1.388(2)
N2–C2, N4–C8	1.378(2), 1.370(2)	N2–C2, N4–C5	1.375(3), 1.376(3)
C2–C3, C8–C9	1.352(2), 1.346(3)	C2–C3, C5–C6	1.351(3), 1.338(3)
N1–Si1–C4, N3–Si2–C10	106.1(1), 106.2(1)	N1–Si1–N3	106.9(1)
N1–Si1–C5, N3–Si2–C11	107.1(1), 107.7(1)	N1–Si1–C7	108.4(1)
N1–Si1–C6, N3–Si2–C12	106.0(1), 106.3(1)	N1–Si1–C8	107.6(1)
C4–Si1–C5, C10–Si2–C11	111.5(1), 112.5(1)	N3–Si1–C7	109.1(1)
C4–Si1–C6, C10–Si2–C12	113.5(1), 112.2(1)	N3–Si1–C8	109.3(1)
C5–Si1–C6, C11–Si2–C12	112.1(1), 111.6(1)	C7–Si1–C8	115.3(1)

The Si atoms’ coordination spheres in silanes SiMe_3_(Im) and SiMe_2_(Im)_2_ are distorted tetrahedral, whereby the distortion can be rationalized by VSEPR effects., i.e., in a systematic manner, the N–Si–C angles in both compounds are similar (and close to the tetrahedral angle), whereas the C–Si–C angles are wider, and in SiMe_2_(Im)_2_, the N–Si–N angle is the smallest in this compound’s Si coordination sphere. In spite of the additional terminal imidazole N atoms available (N2 and N4), both structures are devoid of additional remote lone pair donation toward Si. Instead, these atoms are involved in C–H⋯N contacts. Therefore, in addition to the close to tetrahedral Si-coordination spheres, the typical single/double bond pattern of the imidazole heterocycles is retained in these compounds, e.g., N1–C1=N2–C2=C3–N1. To our knowledge, these are the first crystallographically characterized silanes with unsubstituted imidazolyl substituents. (For the heavier congener, Germanium, the structure of the imidazolyl compound (2,4,6-*t*Bu_3_C_6_H_2_PH)(CH(SiMe_3_)_2_)_2_Ge(Im) has been determined crystallographically [54].) They now complement the structures of derivatives such as the BH_3_-adduct SiMe_2_(Im-BH_3_)_2_ (**AI**, Figure A2) [55], the TiCl_4_-adduct [SiMe_3_(Im)-TiCl_4_]_2_ [56], and 1,3-disilylimidazolium salts [57], none of which possesses a vacant N-donor site in the 3-position. A crystallographically characterized *N*^3^-unsubstituted *N*^1^-SiMe_3_ derivative of an imidazole with substituents in other positions (**AII**) has been published by Purdy et al. [58]. Comparison of the bond length patterns in the imidazole rings of SiMe_3_(Im) and SiMe_2_(Im)_2_ (Table A6) with those of compounds **AI** and **AII** reveals that the formation of the BH_3_ adduct (**AI**) has only a marginal influence on the bonds in the imidazole ring. The substituents at the ring in **AII** have a greater impact, and they cause significant lengthening of Si–N^1^, N^1^–C^1^, and C^2^=C^3^ bonds. Hence, the structure of **AII** cannot serve as an example for bond lengths in “simple imidazolyl silanes”.

With respect to the subject matter of the parent paper, imidazolyl and benzimidazolyl moieties have already been engaged as anchoring groups in chelating ligands used in syntheses of hypercoordinate Si complexes, e.g., in compounds **AIII** [59] and **AIV** [60]. In these compounds, the imidazolyl moiety also features a vacant N-donor site in the 3-position. The Si-coordination mode of the imidazolyl groups in these compounds complements the lone pair donation of imidazole ligands’ N^3^ toward Si as encountered in adducts such as (Aib)SiMe_2_(NMI) [13] and, now, in the parent paper, (Aib)SiMe_2_(HIm).
