# Supplementary material for: Lewis Acid–Base Adducts of α-Amino Isobutyric Acid-Derived Silaheterocycles and Amines
Source: Molecules. 2025 Aug 26;30(17):3501. doi: 10.3390/molecules30173501 (PMC12430707; doi:10.3390/molecules30173501)

checkCIF/PLATON report

Structure factors have been supplied for datablock(s) ASP027dF_01, ASP027d_05, ASP097_02, ASP098_01, asp096_01_sq_sq_sq_sq_sq_sq_sq_sq, asp096e_01, asp167_01, asp173b_01, asp182-st1_01, asp182_01, exp_54_at

THIS REPORT IS FOR GUIDANCE ONLY. IF USED AS PART OF A REVIEW PROCEDURE FOR PUBLICATION, IT SHOULD NOT REPLACE THE EXPERTISE OF AN EXPERIENCED CRYSTALLOGRAPHIC REFEREE.

No syntax errors found.   CIF dictionary   Interpreting this report

Datablock: ASP027d_05

```
Bond precision:   C-C = 0.0023 A          Wavelength=0.71073

Cell:            a=10.1535(3)     b=13.9171(3)        c=10.3250(3)
                 alpha=90         beta=110.254(2)     gamma=90
Temperature:     140 K

                Calculated                    Reported
Volume          1368.78(7)                    1368.78(7)
Space group     P 21/n                        P 21/n
Hall group      -P 2yn                        -P 2yn
Moiety formula  C10 H22 N2 O2 Si              C10 H22 N2 O2 Si
Sum formula     C10 H22 N2 O2 Si              C10 H22 N2 O2 Si
Mr              230.39                        230.38
Dx,g cm-3       1.118                         1.118
Z               4                             4
Mu (mm-1)       0.159                         0.159
F000            504.0                         504.0
F000'           504.50
h,k,lmax        13,18,13                      13,18,13
Nref            3304                          3304
Tmin,Tmax       0.953,0.976
Tmin'           0.938

Correction method= Not given

Data completeness= 1.000           Theta(max)= 27.999

                                                     wR2(reflections)=
R(reflections)= 0.0344( 2679)                        0.0924( 3304)

S = 1.045                 Npar= 156
```

The following ALERTS were generated. Each ALERT has the format
test-name_ALERT_alert-type_alert-level.
Click on the hyperlinks for more details of the test.

Alert level C

```
PLAT230_ALERT_2_C Hirshfeld Test Diff for    Si1      --C5       .        5.9 s.u.
```

Alert level G

```
PLAT002_ALERT_2_G Number of Distance or Angle Restraints on AtSite          8 Note
PLAT168_ALERT_4_G The CIF-Embedded .res File Contains EXYZ Records          4 Report
PLAT171_ALERT_4_G The CIF-Embedded .res File Contains EADP Records          6 Report
PLAT176_ALERT_4_G The CIF-Embedded .res File Contains SADI Records          2 Report
PLAT191_ALERT_3_G A Non-default SADI Restraint Value has been used     0.0010 Report
PLAT191_ALERT_3_G A Non-default SADI Restraint Value has been used     0.0010 Report
PLAT301_ALERT_3_G Main Residue  Disorder ..............(Resd    1)        33% Note
PLAT720_ALERT_4_G Number of Unusual/Non-Standard Labels ..........          1 Note
              H2NA
PLAT811_ALERT_5_G No ADDSYM Analysis: Too Many Excluded Atoms ....          ! Info
PLAT860_ALERT_3_G Number of Least-Squares Restraints ............           7 Note
PLAT967_ALERT_5_G Note: Two-Theta Cutoff Value in Embedded .res ..       56.0 Degree
PLAT969_ALERT_5_G The 'Henn et al.' R-Factor-gap value ...........      4.338 Note
              Predicted wR2: Based on SigI**2  2.13 or SHELX Weight  8.85
PLAT978_ALERT_2_G Number C-C Bonds with Positive Residual Density.          9 Info
```

0 **ALERT level A** = Most likely a serious problem - resolve or explain
0 **ALERT level B** = A potentially serious problem, consider carefully
1 **ALERT level C** = Check. Ensure it is not caused by an omission or oversight
13 **ALERT level G** = General information/check it is not something unexpected

0 ALERT type 1 CIF construction/syntax error, inconsistent or missing data
3 ALERT type 2 Indicator that the structure model may be wrong or deficient
4 ALERT type 3 Indicator that the structure quality may be low
4 ALERT type 4 Improvement, methodology, query or suggestion
3 ALERT type 5 Informative message, check

Datablock: ASP027dF_01

Bond precision:	C-C = 0.0060 A	Wavelength=0.71073	
Cell:	a=15.6692(14)	b=21.4914(18)	c=11.6660(9)
	alpha=90	beta=90	gamma=90
Temperature:	180 K		

	Calculated	Reported
Volume	3928.6(6)	3928.6(6)
Space group	P c c n	P c c n
Hall group	-P 2ab 2ac	-P 2ab 2ac
Moiety formula	2(C10 H22 N2 O2 Si), 3(C H Cl3)	2(C10 H22 N2 O2 Si), 3(C H Cl3)
Sum formula	C23 H47 Cl9 N4 O4 Si2	C23 H47 Cl9 N4 O4 Si2
Mr	818.88	818.87
Dx,g cm-3	1.385	1.384
Z	4	4
Mu (mm-1)	0.736	0.736
F000	1704.0	1704.0
F000'	1710.40	
h,k,lmax	17,24,13	17,24,13
Nref	2907	2905
Tmin,Tmax	0.957,0.971	
Tmin'	0.555	

Correction method= Not given

Data completeness= 0.999 Theta(max)= 23.500

R(reflections)= 0.0493(1463) wR2(reflections)= 0.1077(2905)

S = 1.019 Npar= 276

The following ALERTS were generated. Each ALERT has the format
test-name_ALERT_alert-type_alert-level.
Click on the hyperlinks for more details of the test.

Alert level B

RINTA01_ALERT_3_B The value of Rint is greater than 0.18
Rint given 0.200

Author Response: For this and the other two level B alerts (THETM01 and PLAT023): These crystals had very poor diffraction power. Within the range of data collection, only 50.4 percent of the data were observed. In the upper theta increment between 22.0 deg and 23.5 deg only 16.0 percent of the data were observed. Beyond the upper theta limit used in the refinement, essentially no useful data were available, i.e., in the theta increment between 23.5 deg and 25.0 deg, only 7.8 percent of the data were observed. Therefore, only the data up to theta 23.5 deg were employed in the refinement. The poor signal/noise of the data set is the major reason for the large values of Rint and R1(all data).

```
THETM01_ALERT_3_B  The value of sine(theta_max)/wavelength is less than 0.575
          Calculated sin(theta_max)/wavelength =    0.5610
PLAT023_ALERT_3_B Resolution (too) Low [sin(theta)/Lambda < 0.6]..       0.56 Ang-1
```

Alert level C

```
PLAT260_ALERT_2_C Large Average Ueq of Residue Including       Cl4      0.102 Check
PLAT340_ALERT_3_C Low Bond Precision on  C-C Bonds ...............      0.006 Ang.
PLAT420_ALERT_2_C D-H Bond Without Acceptor  N1       --H1N      .     Please Check
```

Alert level G

```
PLAT002_ALERT_2_G Number of Distance or Angle Restraints on AtSite         24 Note
PLAT020_ALERT_3_G The Value of Rint is Greater Than 0.12 .........      0.200 Report
PLAT063_ALERT_4_G Crystal Size Possibly too Large for Beam Size ..       0.80 mm
PLAT168_ALERT_4_G The CIF-Embedded .res File Contains EXYZ Records          4 Report
PLAT171_ALERT_4_G The CIF-Embedded .res File Contains EADP Records          8 Report
PLAT176_ALERT_4_G The CIF-Embedded .res File Contains SADI Records          5 Report
PLAT191_ALERT_3_G A Non-default SADI Restraint Value has been used     0.0010 Report
PLAT191_ALERT_3_G A Non-default SADI Restraint Value has been used     0.0010 Report
PLAT191_ALERT_3_G A Non-default SADI Restraint Value has been used     0.0010 Report
PLAT299_ALERT_4_G Atom Site Occupancy Constrained at ............         0.5 Check
              Cl4     Cl5     Cl6     C12     H12
PLAT301_ALERT_3_G Main Residue  Disorder ..............(Resd    1)        33% Note
PLAT302_ALERT_4_G Anion/Solvent/Minor-Residue Disorder (Resd    2)       100% Note
PLAT302_ALERT_4_G Anion/Solvent/Minor-Residue Disorder (Resd    3)       100% Note
PLAT302_ALERT_4_G Anion/Solvent/Minor-Residue Disorder (Resd    4)       100% Note
PLAT302_ALERT_4_G Anion/Solvent/Minor-Residue Disorder (Resd    5)       100% Note
PLAT304_ALERT_4_G Non-Integer Number of Atoms in ..... (Resd    2)       2.50 Check
PLAT304_ALERT_4_G Non-Integer Number of Atoms in ..... (Resd    3)       1.65 Check
PLAT304_ALERT_4_G Non-Integer Number of Atoms in ..... (Resd    4)       0.97 Check
PLAT304_ALERT_4_G Non-Integer Number of Atoms in ..... (Resd    5)       2.38 Check
PLAT412_ALERT_2_G Short Intra XH3 .. XHn     H6A      ..H7D      .       2.13 Ang.
                                                     x,y,z  =      1_555 Check
PLAT720_ALERT_4_G Number of Unusual/Non-Standard Labels .........           1 Note
              H2NA
PLAT789_ALERT_4_G Atoms with Negative _atom_site_disorder_group  #          5 Check
PLAT811_ALERT_5_G No ADDSYM Analysis: Too Many Excluded Atoms ....          ! Info
PLAT822_ALERT_4_G CIF-embedded .res Contains Negative PART Numbers          1 Check
PLAT860_ALERT_3_G Number of Least-Squares Restraints ............          94 Note
PLAT910_ALERT_3_G Missing FCF Reflection(s) Below Theta(Min)[Deg]=       2.37 Note
                1  1  0,   0  2  0,
PLAT967_ALERT_5_G Note: Two-Theta Cutoff Value in Embedded .res ..       47.0 Degree
PLAT969_ALERT_5_G The 'Henn et al.' R-Factor-gap value ...........      2.991 Note
              Predicted wR2: Based on SigI**2  3.60 or SHELX Weight 10.57
PLAT978_ALERT_2_G Number C-C Bonds with Positive Residual Density.          0 Info
```

---

   0 **ALERT level A** = Most likely a serious problem - resolve or explain
   3 **ALERT level B** = A potentially serious problem, consider carefully
   3 **ALERT level C** = Check. Ensure it is not caused by an omission or oversight
  29 **ALERT level G** = General information/check it is not something unexpected

   0 ALERT type 1 CIF construction/syntax error, inconsistent or missing data

5 ALERT type 2 Indicator that the structure model may be wrong or deficient
11 ALERT type 3 Indicator that the structure quality may be low
16 ALERT type 4 Improvement, methodology, query or suggestion
3 ALERT type 5 Informative message, check

Datablock: asp096_01_sq_sq_sq_sq_sq_sq_sq_sq

Bond precision: C-C = 0.0034 A Wavelength=0.71073

Cell:	a=10.3734(3)	b=11.2006(4)	c=17.7795(6)
	alpha=105.412(3)	beta=93.183(3)	gamma=98.289(3)
Temperature:	230 K		

	Calculated	Reported
Volume	1961.12(12)	1961.12(12)
Space group	P -1	P -1
Hall group	-P 1	-P 1
Moiety formula	C9 H20 N2 O2 Si, C H Cl3 [+ solvent]	2(C9 H20 N2 O2 Si), 3(C H Cl3)
Sum formula	C10 H21 Cl3 N2 O2 Si [+ solvent]	C21 H43 Cl9 N4 O4 Si2
Mr	335.79	790.82
Dx,g cm-3	1.137	1.339
Z	4	2
Mu (mm-1)	0.526	0.734
F000	704.1	820.0
F000’	706.41	
h,k,lmax	12,13,21	12,13,21
Nref	7703	7701
Tmin,Tmax	0.650,0.693	
Tmin’	0.592	

Correction method= Not given

Data completeness= 1.000 Theta(max)= 26.000

R(reflections)= 0.0446(5088) wR2(reflections)= 0.1443(7701)

S = 1.016 Npar= 482

The following ALERTS were generated. Each ALERT has the format
test-name_ALERT_alert-type_alert-level.
Click on the hyperlinks for more details of the test.

Alert level C

PLAT230_ALERT_2_C Hirshfeld Test Diff for Si1 --C5 . 5.1 s.u.
PLAT260_ALERT_2_C Large Average Ueq of Residue Including Cl1 0.111 Check
PLAT260_ALERT_2_C Large Average Ueq of Residue Including Cl1A 0.137 Check
PLAT260_ALERT_2_C Large Average Ueq of Residue Including Cl1B 0.137 Check
PLAT260_ALERT_2_C Large Average Ueq of Residue Including Cl4A 0.133 Check
PLAT260_ALERT_2_C Large Average Ueq of Residue Including Cl4B 0.130 Check
PLAT352_ALERT_3_C Short N-H (X0.87,N1.01A) N1 - H1N . 0.76 Ang.
PLAT420_ALERT_2_C D-H Bond Without Acceptor Si1 --H1SI . Please Check
PLAT420_ALERT_2_C D-H Bond Without Acceptor Si2 --H2SI . Please Check

Alert level G

FORMU01_ALERT_2_G There is a discrepancy between the atom counts in the
_chemical_formula_sum and the formula from the _atom_site* data.
Atom count from _chemical_formula_sum:C21 H43 Cl9 N4 O4 Si2
Atom count from the _atom_site data: C20.00100 H42. Cl6.003 N4 O4 Si2
CELLZ01_ALERT_1_G Difference between formula and atom_site contents detected.
CELLZ01_ALERT_1_G ALERT: Large difference may be due to a
symmetry error - see SYMMG tests
From the CIF: _cell_formula_units_Z 2
From the CIF: _chemical_formula_sum C21 H43 Cl9 N4 O4 Si2
TEST: Compare cell contents of formula and atom_site data

atom	Z*formula	cif sites	diff
C	42.00	40.00	2.00
H	86.00	84.00	2.00
Cl	18.00	12.01	5.99
N	8.00	8.00	0.00
O	8.00	8.00	0.00
Si	4.00	4.00	0.00

PLAT002_ALERT_2_G Number of Distance or Angle Restraints on AtSite 34 Note
PLAT003_ALERT_2_G Number of Uiso or U(i,j) Restrained non-H-Atoms 1 Report
PLAT041_ALERT_1_G Calc. and Reported SumFormula Strings Differ Please Check
Calc: C10 H21 Cl3 N2 O2 Si
Rep.: C21 H43 Cl9 N4 O4 Si2
PLAT042_ALERT_1_G Calc. and Reported MoietyFormula Strings Differ Please Check
Calc: C9 H20 N2 O2 Si, C H Cl3
Rep.: 2(C9 H20 N2 O2 Si), 3(C H Cl3)
PLAT045_ALERT_1_G Calculated and Reported Z Differ by a Factor ... 2 Check
PLAT051_ALERT_1_G Mu(calc) and Mu(cif) Ratio Differs from 1.0 by . 28.38 %
PLAT063_ALERT_4_G Crystal Size Possibly too Large for Beam Size .. 0.70 mm
PLAT154_ALERT_1_G The s.u.'s on the Cell Angles are Equal ..(Note) 0.003 Degree
PLAT168_ALERT_4_G The CIF-Embedded .res File Contains EXYZ Records 2 Report
PLAT171_ALERT_4_G The CIF-Embedded .res File Contains EADP Records 10 Report
PLAT176_ALERT_4_G The CIF-Embedded .res File Contains SADI Records 6 Report
PLAT186_ALERT_4_G The CIF-Embedded .res File Contains ISOR Records 1 Report
PLAT191_ALERT_3_G A Non-default SADI Restraint Value has been used 0.0010 Report
PLAT191_ALERT_3_G A Non-default SADI Restraint Value has been used 0.0010 Report
PLAT191_ALERT_3_G A Non-default SADI Restraint Value has been used 0.0010 Report
PLAT191_ALERT_3_G A Non-default SADI Restraint Value has been used 0.0100 Report
PLAT191_ALERT_3_G A Non-default SADI Restraint Value has been used 0.0010 Report
PLAT191_ALERT_3_G A Non-default SADI Restraint Value has been used 0.0100 Report
PLAT230_ALERT_2_G Hirshfeld Test Diff for Si2 --N4 . 5.2 s.u.
PLAT230_ALERT_2_G Hirshfeld Test Diff for Si2 --N4A . 5.2 s.u.
PLAT301_ALERT_3_G Main Residue Disorder ....(Resd 1) 36% Note

```
PLAT302_ALERT_4_G Anion/Solvent/Minor-Residue Disorder (Resd    3)       100% Note
PLAT302_ALERT_4_G Anion/Solvent/Minor-Residue Disorder (Resd    4)       100% Note
PLAT302_ALERT_4_G Anion/Solvent/Minor-Residue Disorder (Resd    5)       100% Note
PLAT302_ALERT_4_G Anion/Solvent/Minor-Residue Disorder (Resd    6)       100% Note
PLAT302_ALERT_4_G Anion/Solvent/Minor-Residue Disorder (Resd    7)       100% Note
PLAT302_ALERT_4_G Anion/Solvent/Minor-Residue Disorder (Resd    8)       100% Note
PLAT304_ALERT_4_G Non-Integer Number of Atoms in ..... (Resd    3)       2.48 Check
PLAT304_ALERT_4_G Non-Integer Number of Atoms in ..... (Resd    4)       2.17 Check
PLAT304_ALERT_4_G Non-Integer Number of Atoms in ..... (Resd    5)       0.36 Check
PLAT304_ALERT_4_G Non-Integer Number of Atoms in ..... (Resd    6)       1.45 Check
PLAT304_ALERT_4_G Non-Integer Number of Atoms in ..... (Resd    7)       2.03 Check
PLAT304_ALERT_4_G Non-Integer Number of Atoms in ..... (Resd    8)       1.53 Check
PLAT605_ALERT_4_G Largest Solvent Accessible VOID in the Structure        310 A**3
PLAT720_ALERT_4_G Number of Unusual/Non-Standard Labels ..........          3 Note
              H1SI    H2SI    H4NA
PLAT811_ALERT_5_G No ADDSYM Analysis: Too Many Excluded Atoms ....          ! Info
PLAT860_ALERT_3_G Number of Least-Squares Restraints .............        173 Note
PLAT869_ALERT_4_G ALERTS Related to the Use of SQUEEZE  Suppressed          ! Info
PLAT912_ALERT_4_G Missing # of FCF Reflections Above STh/L=  0.600          2 Note
PLAT967_ALERT_5_G Note: Two-Theta Cutoff Value in Embedded .res ..       52.0 Degree
PLAT969_ALERT_5_G The 'Henn et al.' R-Factor-gap value ..........      4.270 Note
              Predicted wR2: Based on SigI**2  3.38 or SHELX Weight 14.27
PLAT978_ALERT_2_G Number C-C Bonds with Positive Residual Density.          1 Info
```

---

0 **ALERT level A** = Most likely a serious problem - resolve or explain
0 **ALERT level B** = A potentially serious problem, consider carefully
9 **ALERT level C** = Check. Ensure it is not caused by an omission or oversight
45 **ALERT level G** = General information/check it is not something unexpected

7 ALERT type 1 CIF construction/syntax error, inconsistent or missing data
14 ALERT type 2 Indicator that the structure model may be wrong or deficient
9 ALERT type 3 Indicator that the structure quality may be low
21 ALERT type 4 Improvement, methodology, query or suggestion
3 ALERT type 5 Informative message, check

Datablock: asp096e_01

Bond precision:	C-C = 0.0042 A		Wavelength=0.71073
Cell:	a=12.9491(8)	b=17.6134(8)	c=11.0312(5)
	alpha=90	beta=97.538(4)	gamma=90
Temperature:	160 K		

	Calculated	Reported
Volume	2494.2(2)	2494.2(2)
Space group	C 2/c	C 2/c
Hall group	-C 2yc	-C 2yc
Moiety formula	C9 H20 N2 O2 Si	C9 H20 N2 O2 Si
Sum formula	C9 H20 N2 O2 Si	C9 H20 N2 O2 Si
Mr	216.36	216.36
Dx,g cm-3	1.152	1.152
Z	8	8
Mu (mm-1)	0.170	0.170
F000	944.0	944.0
F000'	944.98	
h,k,lmax	15,20,13	15,20,13
Nref	2189	2186
Tmin,Tmax	0.954,0.988	0.911,0.986
Tmin'	0.911	

Correction method= # Reported T Limits: Tmin=0.911 Tmax=0.986
AbsCorr = INTEGRATION

Data completeness= 0.999 Theta(max)= 24.978

R(reflections)= 0.0446(1359) wR2(reflections)= 0.1163(2186)

S = 0.971 Npar= 139

The following ALERTS were generated. Each ALERT has the format
test-name_ALERT_alert-type_alert-level.
Click on the hyperlinks for more details of the test.

Alert level C

```
PLAT241_ALERT_2_C High   'MainMol' Ueq as Compared to Neighbors of         C8 Check
PLAT340_ALERT_3_C Low Bond Precision on  C-C Bonds ...............    0.00417 Ang.
PLAT352_ALERT_3_C Short  N-H (X0.87,N1.01A)  N1       - H1N      .       0.74 Ang.
PLAT352_ALERT_3_C Short  N-H (X0.87,N1.01A)  N2       - H2N      .       0.74 Ang.
PLAT420_ALERT_2_C D-H Bond Without Acceptor  Si1      --H1SI     .     Please Check
```

Alert level G

```
PLAT066_ALERT_1_G Predicted and Reported Tmin&Tmax Range Identical           ? Check
PLAT720_ALERT_4_G Number of Unusual/Non-Standard Labels .........           1 Note
              H1SI
PLAT910_ALERT_3_G Missing FCF Reflection(s) Below Theta(Min)[Deg]=       2.56 Note
                1  1  0,   0  2  0,
PLAT967_ALERT_5_G Note: Two-Theta Cutoff Value in Embedded .res ..       50.0 Degree
PLAT969_ALERT_5_G The 'Henn et al.' R-Factor-gap value ..........      2.921 Note
              Predicted wR2: Based on SigI**2  3.98 or SHELX Weight 11.98
PLAT978_ALERT_2_G Number C-C Bonds with Positive Residual Density.          1 Info
```

0 **ALERT level A** = Most likely a serious problem - resolve or explain
0 **ALERT level B** = A potentially serious problem, consider carefully
5 **ALERT level C** = Check. Ensure it is not caused by an omission or oversight
6 **ALERT level G** = General information/check it is not something unexpected

1 ALERT type 1 CIF construction/syntax error, inconsistent or missing data
3 ALERT type 2 Indicator that the structure model may be wrong or deficient
4 ALERT type 3 Indicator that the structure quality may be low
1 ALERT type 4 Improvement, methodology, query or suggestion
2 ALERT type 5 Informative message, check

Datablock: ASP097_02

Bond precision: C-C = 0.0032 A   Wavelength=0.71073

Cell:	a=12.9780(4)	b=12.1486(3)	c=17.9866(6)
	alpha=90	beta=102.055(3)	gamma=90
Temperature:	180 K		

	Calculated	Reported
Volume	2773.31(15)	2773.31(15)
Space group	P 21/c	P 21/c
Hall group	-P 2ybc	-P 2ybc
Moiety formula	C11 H22 N2 O2 Si	C11 H22 N2 O2 Si
Sum formula	C11 H22 N2 O2 Si	C11 H22 N2 O2 Si
Mr	242.40	242.39
Dx,g cm-3	1.161	1.161
Z	8	8
Mu (mm-1)	0.160	0.160
F000	1056.0	1056.0
F000’	1057.02	
h,k,lmax	16,14,22	16,14,22
Nref	5430	5428
Tmin,Tmax	0.953,0.976	
Tmin’	0.923	

Correction method= Not given

Data completeness= 1.000   Theta(max)= 25.998

R(reflections)= 0.0412(4039)   wR2(reflections)= 0.1031(5428)

S = 1.041   Npar= 393

The following ALERTS were generated. Each ALERT has the format
test-name_ALERT_alert-type_alert-level.
Click on the hyperlinks for more details of the test.

Alert level C

```
PLAT242_ALERT_2_C Low    'MainMol' Ueq as Compared to Neighbors of        C13 Check
PLAT352_ALERT_3_C Short  N-H (X0.87,N1.01A)  N1        - H1N      .       0.76 Ang.
PLAT352_ALERT_3_C Short  N-H (X0.87,N1.01A)  N3        - H3N      .       0.76 Ang.
PLAT906_ALERT_3_C Large K Value in the Analysis of Variance ......      3.340 Check
```

Alert level G

```
PLAT002_ALERT_2_G Number of Distance or Angle Restraints on AtSite         34 Note
PLAT168_ALERT_4_G The CIF-Embedded .res File Contains EXYZ Records          2 Report
PLAT171_ALERT_4_G The CIF-Embedded .res File Contains EADP Records         10 Report
PLAT176_ALERT_4_G The CIF-Embedded .res File Contains SADI Records         16 Report
PLAT191_ALERT_3_G A Non-default SADI Restraint Value has been used     0.0010 Report
PLAT191_ALERT_3_G A Non-default SADI Restraint Value has been used     0.0010 Report
PLAT191_ALERT_3_G A Non-default SADI Restraint Value has been used     0.0010 Report
PLAT191_ALERT_3_G A Non-default SADI Restraint Value has been used     0.0100 Report
PLAT191_ALERT_3_G A Non-default SADI Restraint Value has been used     0.0100 Report
PLAT191_ALERT_3_G A Non-default SADI Restraint Value has been used     0.0010 Report
PLAT191_ALERT_3_G A Non-default SADI Restraint Value has been used     0.0010 Report
PLAT191_ALERT_3_G A Non-default SADI Restraint Value has been used     0.0010 Report
PLAT191_ALERT_3_G A Non-default SADI Restraint Value has been used     0.0100 Report
PLAT191_ALERT_3_G A Non-default SADI Restraint Value has been used     0.0100 Report
PLAT191_ALERT_3_G A Non-default SADI Restraint Value has been used     0.0010 Report
PLAT191_ALERT_3_G A Non-default SADI Restraint Value has been used     0.0010 Report
PLAT191_ALERT_3_G A Non-default SADI Restraint Value has been used     0.0010 Report
PLAT191_ALERT_3_G A Non-default SADI Restraint Value has been used     0.0010 Report
PLAT191_ALERT_3_G A Non-default SADI Restraint Value has been used     0.0010 Report
PLAT191_ALERT_3_G A Non-default SADI Restraint Value has been used     0.0010 Report
PLAT301_ALERT_3_G Main Residue  Disorder .............(Resd    1)        50% Note
PLAT301_ALERT_3_G Main Residue  Disorder .............(Resd    2)        50% Note
PLAT414_ALERT_2_G Short Intra D-H..H-X       H2N      ..H8A      .       2.10 Ang.
                                                      x,y,z  =     1_555 Check
PLAT414_ALERT_2_G Short Intra D-H..H-X       H2N      ..H11B     .       2.10 Ang.
                                                      x,y,z  =     1_555 Check
PLAT414_ALERT_2_G Short Intra D-H..H-X       H2N      ..H8C      .       2.10 Ang.
                                                      x,y,z  =     1_555 Check
PLAT414_ALERT_2_G Short Intra D-H..H-X       H2N      ..H11D     .       2.11 Ang.
                                                      x,y,z  =     1_555 Check
PLAT414_ALERT_2_G Short Intra D-H..H-X       H4N      ..H22A     .       2.06 Ang.
                                                      x,y,z  =     1_555 Check
PLAT414_ALERT_2_G Short Intra D-H..H-X       H4N      ..H19D     .       2.00 Ang.
                                                      x,y,z  =     1_555 Check
PLAT811_ALERT_5_G No ADDSYM Analysis: Too Many Excluded Atoms ....          ! Info
PLAT860_ALERT_3_G Number of Least-Squares Restraints ............          31 Note
PLAT910_ALERT_3_G Missing FCF Reflection(s) Below Theta(Min)[Deg]=       2.32 Note
                1  0  0,   0  1  1,
PLAT941_ALERT_3_G Average HKL Measurement Multiplicity ..........         4.9 Low
PLAT967_ALERT_5_G Note: Two-Theta Cutoff Value in Embedded .res ..       52.0 Degree
PLAT969_ALERT_5_G The 'Henn et al.' R-Factor-gap value ..........       5.094 Note
              Predicted wR2: Based on SigI**2  2.02 or SHELX Weight  9.91
PLAT978_ALERT_2_G Number C-C Bonds with Positive Residual Density.          4 Info
```

```
  0 ALERT level A = Most likely a serious problem - resolve or explain
  0 ALERT level B = A potentially serious problem, consider carefully
  4 ALERT level C = Check. Ensure it is not caused by an omission or oversight
 35 ALERT level G = General information/check it is not something unexpected

  0 ALERT type 1 CIF construction/syntax error, inconsistent or missing data
  9 ALERT type 2 Indicator that the structure model may be wrong or deficient
 24 ALERT type 3 Indicator that the structure quality may be low
  3 ALERT type 4 Improvement, methodology, query or suggestion
  3 ALERT type 5 Informative message, check
```

Datablock: ASP098_01

Bond precision: C-C = 0.0024 A   Wavelength=0.71073

Cell:	a=10.1751(4)	b=14.8904(6)	c=10.5661(3)
	alpha=90	beta=109.834(2)	gamma=90
Temperature:	180 K		

	Calculated	Reported
Volume	1505.92(10)	1505.92(10)
Space group	P 21/n	P 21/n
Hall group	-P 2yn	-P 2yn
Moiety formula	C12 H26 N2 O2 Si	C12 H26 N2 O2 Si
Sum formula	C12 H26 N2 O2 Si	C12 H26 N2 O2 Si
Mr	258.44	258.44
Dx,g cm-3	1.140	1.140
Z	4	4
Mu (mm-1)	0.151	0.151
F000	568.0	568.0
F000’	568.52	
h,k,lmax	13,19,13	13,19,13
Nref	3626	3627
Tmin,Tmax	0.964,0.978	
Tmin’	0.913	

Correction method= Not given

Data completeness= 1.000   Theta(max)= 27.994

R(reflections)= 0.0405(2715)   wR2(reflections)= 0.1061(3627)

S = 1.021   Npar= 182

The following ALERTS were generated. Each ALERT has the format
test-name_ALERT_alert-type_alert-level.
Click on the hyperlinks for more details of the test.

Alert level C

```
PLAT906_ALERT_3_C Large K Value in the Analysis of Variance ......      2.162 Check
```

Alert level G

```
PLAT002_ALERT_2_G Number of Distance or Angle Restraints on AtSite         10 Note
PLAT168_ALERT_4_G The CIF-Embedded .res File Contains EXYZ Records          3 Report
PLAT171_ALERT_4_G The CIF-Embedded .res File Contains EADP Records          5 Report
PLAT176_ALERT_4_G The CIF-Embedded .res File Contains SADI Records          2 Report
PLAT191_ALERT_3_G A Non-default SADI Restraint Value has been used     0.0010 Report
PLAT191_ALERT_3_G A Non-default SADI Restraint Value has been used     0.0010 Report
PLAT301_ALERT_3_G Main Residue  Disorder .............(Resd    1)         29% Note
PLAT411_ALERT_2_G Short Inter H...H Contact  H5A      ..H10C     .       2.14 Ang.
                                  1/2+x,1/2-y,1/2+z  =      4_666 Check
PLAT720_ALERT_4_G Number of Unusual/Non-Standard Labels .........           1 Note
              H2NA
PLAT811_ALERT_5_G No ADDSYM Analysis: Too Many Excluded Atoms ....          ! Info
PLAT860_ALERT_3_G Number of Least-Squares Restraints ............          21 Note
PLAT941_ALERT_3_G Average HKL Measurement Multiplicity ..........         3.8 Low
PLAT967_ALERT_5_G Note: Two-Theta Cutoff Value in Embedded .res ..       56.0 Degree
PLAT969_ALERT_5_G The 'Henn et al.' R-Factor-gap value ..........       3.950 Note
              Predicted wR2: Based on SigI**2  2.69 or SHELX Weight 10.39
PLAT978_ALERT_2_G Number C-C Bonds with Positive Residual Density.          6 Info
```

0 **ALERT level A** = Most likely a serious problem - resolve or explain
0 **ALERT level B** = A potentially serious problem, consider carefully
1 **ALERT level C** = Check. Ensure it is not caused by an omission or oversight
15 **ALERT level G** = General information/check it is not something unexpected

0 ALERT type 1 CIF construction/syntax error, inconsistent or missing data
3 ALERT type 2 Indicator that the structure model may be wrong or deficient
6 ALERT type 3 Indicator that the structure quality may be low
4 ALERT type 4 Improvement, methodology, query or suggestion
3 ALERT type 5 Informative message, check

Datablock: asp167_01

Bond precision:	C-C = 0.0074 A		Wavelength=0.71073
Cell:	a=9.9795(4)	b=13.4515(8)	c=10.2116(4)
	alpha=90	beta=111.317(3)	gamma=90
Temperature:	140 K		

	Calculated	Reported
Volume	1277.01(11)	1277.01(11)
Space group	P n	P n
Hall group	P -2yac	P -2yac
Moiety formula	C9 H22 N2 O2 Si	C9 H22 N2 O2 Si
Sum formula	C9 H22 N2 O2 Si	C9 H22 N2 O2 Si
Mr	218.38	218.37
Dx,g cm-3	1.136	1.136
Z	4	4
Mu (mm-1)	0.166	0.166
F000	480.0	480.0
F000'	480.49	
h,k,lmax	12,16,12	12,16,12
Nref	5026[2521]	4861
Tmin,Tmax	0.984,0.997	
Tmin'	0.876	

Correction method= Not given

Data completeness= 1.93/0.97 Theta(max)= 25.994

R(reflections)= 0.0474(3425) wR2(reflections)= 0.1030(4861)

S = 0.993 Npar= 289

The following ALERTS were generated. Each ALERT has the format
test-name_ALERT_alert-type_alert-level.
Click on the hyperlinks for more details of the test.

Alert level C

```
STRVA01_ALERT_4_C           Flack test results are ambiguous.
          From the CIF: _refine_ls_abs_structure_Flack    0.330
          From the CIF: _refine_ls_abs_structure_Flack_su    0.150
PLAT340_ALERT_3_C Low Bond Precision on  C-C Bonds ...............    0.00738 Ang.
```

Alert level G

```
PLAT002_ALERT_2_G Number of Distance or Angle Restraints on AtSite          3 Note
PLAT063_ALERT_4_G Crystal Size Possibly too Large for Beam Size ..       0.80 mm
PLAT168_ALERT_4_G The CIF-Embedded .res File Contains EXYZ Records          1 Report
PLAT171_ALERT_4_G The CIF-Embedded .res File Contains EADP Records          3 Report
PLAT172_ALERT_4_G The CIF-Embedded .res File Contains DFIX Records          1 Report
PLAT301_ALERT_3_G Main Residue  Disorder .............(Resd    1)         21% Note
PLAT412_ALERT_2_G Short Intra XH3 .. XHn      H6A       ..H7A      .       2.09 Ang.
                                                    x,y,z  =      1_555 Check
PLAT413_ALERT_2_G Short Inter XH3 .. XHn      H18C      ..H9A1     .       1.94 Ang.
                                        -1/2+x,1-y,-3/2+z  =      2_463 Check
PLAT720_ALERT_4_G Number of Unusual/Non-Standard Labels .........          9 Note
```

```
             H2NA    H2NB    H8A1    H8A2    H9A1    H9A2    H9A3    H4NA
             H4NB
PLAT860_ALERT_3_G Number of Least-Squares Restraints ............          4 Note
PLAT910_ALERT_3_G Missing FCF Reflection(s) Below Theta(Min)[Deg]=      2.44 Note
               0  1  0,
PLAT967_ALERT_5_G Note: Two-Theta Cutoff Value in Embedded .res ..      52.0 Degree
PLAT969_ALERT_5_G The 'Henn et al.' R-Factor-gap value ..........      3.006 Note
             Predicted wR2: Based on SigI**2  3.42 or SHELX Weight 10.37
PLAT978_ALERT_2_G Number C-C Bonds with Positive Residual Density.         0 Info
```

```
   0 ALERT level A = Most likely a serious problem - resolve or explain
   0 ALERT level B = A potentially serious problem, consider carefully
   2 ALERT level C = Check. Ensure it is not caused by an omission or oversight
  14 ALERT level G = General information/check it is not something unexpected

   0 ALERT type 1 CIF construction/syntax error, inconsistent or missing data
   4 ALERT type 2 Indicator that the structure model may be wrong or deficient
   4 ALERT type 3 Indicator that the structure quality may be low
   6 ALERT type 4 Improvement, methodology, query or suggestion
   2 ALERT type 5 Informative message, check
```

Datablock: asp173b_01

Bond precision: C-C = 0.0020 A Wavelength=0.71073

Cell:	a=10.9384(4)	b=16.7355(6)	c=17.7731(9)
	alpha=90	beta=90	gamma=90
Temperature:	180 K		

	Calculated	Reported
Volume	3253.5(2)	3253.5(2)
Space group	P b c a	P b c a
Hall group	-P 2ac 2ab	-P 2ac 2ab
Moiety formula	C9 H17 N3 O2 Si, C H Cl3	C9 H17 N3 O2 Si, C H Cl3
Sum formula	C10 H18 Cl3 N3 O2 Si	C10 H18 Cl3 N3 O2 Si
Mr	346.71	346.71
Dx,g cm-3	1.416	1.416
Z	8	8
Mu (mm-1)	0.638	0.638
F000	1440.0	1440.0
F000'	1444.59	
h,k,lmax	13,21,22	13,21,22
Nref	3542	3541
Tmin,Tmax	0.800,0.853	0.835,0.886
Tmin'	0.800	

```
Correction method= # Reported T Limits: Tmin=0.835 Tmax=0.886
AbsCorr = INTEGRATION

Data completeness= 1.000           Theta(max)= 26.997

                                                wR2(reflections)=
R(reflections)= 0.0293( 2733)                   0.0729( 3541)

S = 1.049                   Npar= 184
```

The following ALERTS were generated. Each ALERT has the format
test-name_ALERT_alert-type_alert-level.
Click on the hyperlinks for more details of the test.

Alert level C

```
PLAT230_ALERT_2_C Hirshfeld Test Diff for    Si1      --C5       .        5.6 s.u.
PLAT244_ALERT_4_C Low    'Solvent' Ueq as Compared to Neighbors of        C10 Check
PLAT420_ALERT_2_C D-H Bond Without Acceptor  N1       --H1N      .     Please Check
PLAT906_ALERT_3_C Large K Value in the Analysis of Variance ......      2.201 Check
```

Alert level G

```
PLAT432_ALERT_2_G Short Inter X...Y Contact  O2       ..C10      .       2.94 Ang.
                                                  x,y,z  =      1_555 Check
PLAT910_ALERT_3_G Missing FCF Reflection(s) Below Theta(Min)[Deg]=       2.43 Note
                0  0  2,
PLAT967_ALERT_5_G Note: Two-Theta Cutoff Value in Embedded .res ..       54.0 Degree
PLAT969_ALERT_5_G The 'Henn et al.' R-Factor-gap value ..........      5.311 Note
              Predicted wR2: Based on SigI**2  1.37 or SHELX Weight  6.95
PLAT978_ALERT_2_G Number C-C Bonds with Positive Residual Density.          4 Info
```

---

0 **ALERT level A** = Most likely a serious problem - resolve or explain
0 **ALERT level B** = A potentially serious problem, consider carefully
4 **ALERT level C** = Check. Ensure it is not caused by an omission or oversight
5 **ALERT level G** = General information/check it is not something unexpected

0 ALERT type 1 CIF construction/syntax error, inconsistent or missing data
4 ALERT type 2 Indicator that the structure model may be wrong or deficient
2 ALERT type 3 Indicator that the structure quality may be low
1 ALERT type 4 Improvement, methodology, query or suggestion
2 ALERT type 5 Informative message, check

Datablock: asp182_01

```
Bond precision:   C-C = 0.0030 A           Wavelength=0.71073
```

Cell:	a=21.6042(8)	b=7.8214(2)	c=12.0118(3)
	alpha=90	beta=90	gamma=90
Temperature:	180 K		

	Calculated	Reported
Volume	2029.70(10)	2029.70(10)
Space group	P c c n	P c c n
Hall group	-P 2ab 2ac	-P 2ab 2ac
Moiety formula	C8 H12 N4 Si	C8 H12 N4 Si
Sum formula	C8 H12 N4 Si	C8 H12 N4 Si
Mr	192.31	192.31
Dx,g cm-3	1.259	1.259
Z	8	8
Mu (mm-1)	0.192	0.192
F000	816.0	816.0
F000'	816.81	
h,k,lmax	26,9,14	26,9,14
Nref	2001	1999
Tmin,Tmax	0.902,0.981	0.940,0.989
Tmin'	0.900	

Correction method= # Reported T Limits: Tmin=0.940 Tmax=0.989
AbsCorr = INTEGRATION

Data completeness= 0.999 Theta(max)= 26.000

R(reflections)= 0.0424(1520) wR2(reflections)= 0.1215(1999)

S = 0.995 Npar= 120

The following ALERTS were generated. Each ALERT has the format
test-name_ALERT_alert-type_alert-level.
Click on the hyperlinks for more details of the test.

Alert level C

PLAT906_ALERT_3_C Large K Value in the Analysis of Variance ...... 2.346 Check

---

Alert level G

PLAT910_ALERT_3_G Missing FCF Reflection(s) Below Theta(Min)[Deg]= 2.77 Note
2 0 0,
PLAT967_ALERT_5_G Note: Two-Theta Cutoff Value in Embedded .res .. 52.0 Degree
PLAT969_ALERT_5_G The 'Henn et al.' R-Factor-gap value .... 4.960 Note
Predicted wR2: Based on SigI**2 2.45 or SHELX Weight 12.21
PLAT978_ALERT_2_G Number C-C Bonds with Positive Residual Density. 0 Info

---

0 **ALERT level A** = Most likely a serious problem - resolve or explain
0 **ALERT level B** = A potentially serious problem, consider carefully
1 **ALERT level C** = Check. Ensure it is not caused by an omission or oversight
4 **ALERT level G** = General information/check it is not something unexpected

0 ALERT type 1 CIF construction/syntax error, inconsistent or missing data
1 ALERT type 2 Indicator that the structure model may be wrong or deficient
2 ALERT type 3 Indicator that the structure quality may be low
0 ALERT type 4 Improvement, methodology, query or suggestion
2 ALERT type 5 Informative message, check

Datablock: asp182-st1_01

Bond precision: C-C = 0.0025 A Wavelength=0.71073

Cell:	a=12.7551(4)	b=10.6258(2)	c=13.0791(3)
	alpha=90	beta=106.961(2)	gamma=90
Temperature:	200 K		

	Calculated	Reported
Volume	1695.55(8)	1695.55(8)
Space group	P 21/c	P 21/c
Hall group	-P 2ybc	-P 2ybc
Moiety formula	C6 H12 N2 Si	C6 H12 N2 Si
Sum formula	C6 H12 N2 Si	C6 H12 N2 Si
Mr	140.27	140.27
Dx,g cm-3	1.099	1.099
Z	8	8
Mu (mm-1)	0.201	0.201
F000	608.0	608.0
F000’	608.76	
h,k,lmax	15,13,16	15,13,16
Nref	3325	3323
Tmin,Tmax	0.786,0.818	0.300,0.696
Tmin’	0.018	

Correction method= # Reported T Limits: Tmin=0.300 Tmax=0.696
AbsCorr = INTEGRATION

Data completeness= 0.999 Theta(max)= 25.999

R(reflections)= 0.0401(3050) wR2(reflections)= 0.1117(3323)

S = 1.040 Npar= 170

The following ALERTS were generated. Each ALERT has the format
test-name_ALERT_alert-type_alert-level.
Click on the hyperlinks for more details of the test.

Alert level A

PLAT063_ALERT_4_A Crystal Size Possibly too Large for Beam Size .. 20. mm

Author Response: For this and the three related level B crystal size alerts CRYSS02: The compound was crystallized inside a glass capillary. The dimensions of the crystal itself were smaller, but the dimensions of the relevant capillary section are reported, because this volume of the capillary was filled with solid and liquid of the composition of the crystal and thus contributed to the absorption.

Alert level B

```
CRYSS02_ALERT_3_B  The value of _exptl_crystal_size_min is > 0.6
   Minimum crystal size given =   1.000
CRYSS02_ALERT_3_B  The value of _exptl_crystal_size_mid is > 0.8
   Mid crystal size given     =   1.000
CRYSS02_ALERT_3_B  The value of _exptl_crystal_size_max is > 1.0
   Maximum crystal size given =  20.000
```

Alert level C

```
PLAT230_ALERT_2_C Hirshfeld Test Diff for    Si1      --C5       .         5.4 s.u.
PLAT911_ALERT_3_C Missing FCF Refl Between Thmin & STh/L=    0.600           2 Report
                1  1   0, -12  0  2,
```

Alert level G

```
PLAT933_ALERT_2_G Number of HKL-OMIT Records in Embedded .res File           2 Note
              -12  0  2,   1  1  0,
PLAT967_ALERT_5_G Note: Two-Theta Cutoff Value in Embedded .res ..       52.0 Degree
PLAT969_ALERT_5_G The 'Henn et al.' R-Factor-gap value ..........      3.096 Note
              Predicted wR2: Based on SigI**2  3.61 or SHELX Weight 10.74
PLAT978_ALERT_2_G Number C-C Bonds with Positive Residual Density.           0 Info
```

1 **ALERT level A** = Most likely a serious problem - resolve or explain
3 **ALERT level B** = A potentially serious problem, consider carefully
2 **ALERT level C** = Check. Ensure it is not caused by an omission or oversight
4 **ALERT level G** = General information/check it is not something unexpected

0 ALERT type 1 CIF construction/syntax error, inconsistent or missing data
3 ALERT type 2 Indicator that the structure model may be wrong or deficient
4 ALERT type 3 Indicator that the structure quality may be low
1 ALERT type 4 Improvement, methodology, query or suggestion
2 ALERT type 5 Informative message, check

Datablock: exp_54_at

Bond precision:	C-C = 0.0025 A		Wavelength=1.54184
Cell:	a=10.4171(1)	b=11.9432(1)	c=12.8295(1)
	alpha=96.129(1)	beta=106.033(1)	gamma=105.231(1)
Temperature:	100 K		

	Calculated	Reported
Volume	1452.25(3)	1452.25(2)
Space group	P -1	P -1
Hall group	-P 1	-P 1
Moiety formula	C9 H17 N2 O4 Si, C4 H12 N, C4 H11 N, C H Cl3	C9 H17 N2 O4 Si, C4 H12 N, C4 H11 N, C H Cl3
Sum formula	C18 H41 Cl3 N4 O4 Si	C18 H41 Cl3 N4 O4 Si
Mr	511.99	511.99
Dx,g cm-3	1.171	1.171
Z	2	2
Mu (mm-1)	3.476	3.476
F000	548.0	548.0
F000'	551.93	
h,k,lmax	13,14,16	13,14,16
Nref	5909	5891
Tmin,Tmax	0.424,0.502	0.411,1.000
Tmin'	0.170	

Correction method= # Reported T Limits: Tmin=0.411 Tmax=1.000
AbsCorr = GAUSSIAN

Data completeness= 0.997 Theta(max)= 74.495

R(reflections)= 0.0379(5648) wR2(reflections)= 0.1002(5891)

S = 1.040 Npar= 310

The following ALERTS were generated. Each ALERT has the format
test-name_ALERT_alert-type_alert-level.
Click on the hyperlinks for more details of the test.

Alert level C

```
PLAT244_ALERT_4_C Low    'Solvent' Ueq as Compared to Neighbors of        C18 Check
PLAT420_ALERT_2_C D-H Bond Without Acceptor  N1       --H1       .     Please Check
PLAT420_ALERT_2_C D-H Bond Without Acceptor  N2       --H2       .     Please Check
PLAT420_ALERT_2_C D-H Bond Without Acceptor  N4       --H4D      .     Please Check
```

```
PLAT420_ALERT_2_C D-H Bond Without Acceptor  N4       --H4E      .     Please Check
PLAT911_ALERT_3_C Missing FCF Refl Between Thmin & STh/L=    0.600          5 Report
               -5   0   4,   10 -7   5,    9 -8   6,    9 -7   6,    9 -6   7,
```

Alert level G

```
PLAT154_ALERT_1_G The s.u.'s on the Cell Angles are Equal ..(Note)      0.001 Degree
PLAT432_ALERT_2_G Short Inter X...Y Contact  O4       ..C18      .       3.00 Ang.
                                                2-x,1-y,1-z  =      2_766 Check
PLAT484_ALERT_4_G Round D-H..A Angle Rep for C18      ..O4       .        152 Degree
PLAT912_ALERT_4_G Missing # of FCF Reflections Above STh/L=  0.600         13 Note
PLAT933_ALERT_2_G Number of HKL-OMIT Records in Embedded .res File          1 Note
               -5   0   4,
PLAT969_ALERT_5_G The 'Henn et al.' R-Factor-gap value ..........      5.926 Note
              Predicted wR2: Based on SigI**2  1.69 or SHELX Weight  9.64
PLAT978_ALERT_2_G Number C-C Bonds with Positive Residual Density.         10 Info
```

---

   0 **ALERT level A** = Most likely a serious problem - resolve or explain
   0 **ALERT level B** = A potentially serious problem, consider carefully
   6 **ALERT level C** = Check. Ensure it is not caused by an omission or oversight
   7 **ALERT level G** = General information/check it is not something unexpected

   1 ALERT type 1 CIF construction/syntax error, inconsistent or missing data
   7 ALERT type 2 Indicator that the structure model may be wrong or deficient
   1 ALERT type 3 Indicator that the structure quality may be low
   3 ALERT type 4 Improvement, methodology, query or suggestion
   1 ALERT type 5 Informative message, check

---

checkCIF publication errors

Alert level A

```
PUBL004_ALERT_1_A  The contact author's name and address are missing,
            _publ_contact_author_name and _publ_contact_author_address.
PUBL005_ALERT_1_A  _publ_contact_author_email, _publ_contact_author_fax and
            _publ_contact_author_phone are all missing.
            At least one of these should be present.
PUBL006_ALERT_1_A  _publ_requested_journal is missing
            e.g. 'Acta Crystallographica Section C'
PUBL008_ALERT_1_A  _publ_section_title is missing. Title of paper.
PUBL009_ALERT_1_A  _publ_author_name is missing. List of author(s) name(s).
PUBL010_ALERT_1_A  _publ_author_address is missing. Author(s) address(es).
PUBL012_ALERT_1_A  _publ_section_abstract is missing.
            Abstract of paper in English.
```

---

   7 **ALERT level A** = Data missing that is essential or data in wrong format
   0 **ALERT level G** = General alerts. Data that may be required is missing

---

Publication of your CIF

You should attempt to resolve as many as possible of the alerts in all categories. Often the minor alerts point to easily fixed oversights, errors and omissions in your CIF or refinement strategy, so attention to these fine details can be worthwhile. In order to resolve some of the more serious problems it may be necessary to carry out additional measurements or structure refinements. However, the nature of your study may justify the reported deviations from journal submission requirements and the more serious of these should be commented upon in the discussion or experimental section of a paper or in the "special_details" fields of the CIF. *checkCIF* was carefully designed to identify outliers and unusual parameters, but every test has its limitations and alerts that are not important in a particular case may appear. Conversely, the absence of alerts does not guarantee there are no aspects of the results needing attention. It is up to the individual to critically assess their own results and, if necessary, seek expert advice.

If level A alerts remain, which you believe to be justified deviations, and you intend to submit this CIF for publication in a journal, you should additionally insert an explanation in your CIF using the Validation Reply Form (VRF) below. This will allow your explanation to be considered as part of the review process.

Validation response form

Please find below a validation response form (VRF) that can be filled in and pasted into your CIF.

```
# start Validation Reply Form
_vrf_PUBL004_GLOBAL
;
PROBLEM: The contact author's name and address are missing,
RESPONSE: ...
;
_vrf_PUBL005_GLOBAL
;
PROBLEM: _publ_contact_author_email, _publ_contact_author_fax and
RESPONSE: ...
;
_vrf_PUBL006_GLOBAL
;
PROBLEM: _publ_requested_journal is missing
RESPONSE: ...
;
_vrf_PUBL008_GLOBAL
;
PROBLEM: _publ_section_title is missing. Title of paper.
RESPONSE: ...
;
_vrf_PUBL009_GLOBAL
;
PROBLEM: _publ_author_name is missing. List of author(s) name(s).
RESPONSE: ...
;
_vrf_PUBL010_GLOBAL
;
PROBLEM: _publ_author_address is missing. Author(s) address(es).
```

```
RESPONSE: ...
;
_vrf_PUBL012_GLOBAL
;
PROBLEM: _publ_section_abstract is missing.
RESPONSE: ...
;
# end Validation Reply Form
```

If you wish to submit your CIF for publication in Acta Crystallographica Section C or E, you should upload your CIF via the web. If you wish to submit your CIF for publication in IUCrData you should upload your CIF via the web. If your CIF is to form part of a submission to another IUCr journal, you will be asked, either during electronic submission or by the Co-editor handling your paper, to upload your CIF via our web site.

PLATON version of 04/06/2025; check.def file version of 30/05/2025

Datablock ASP027d_05 - ellipsoid plot

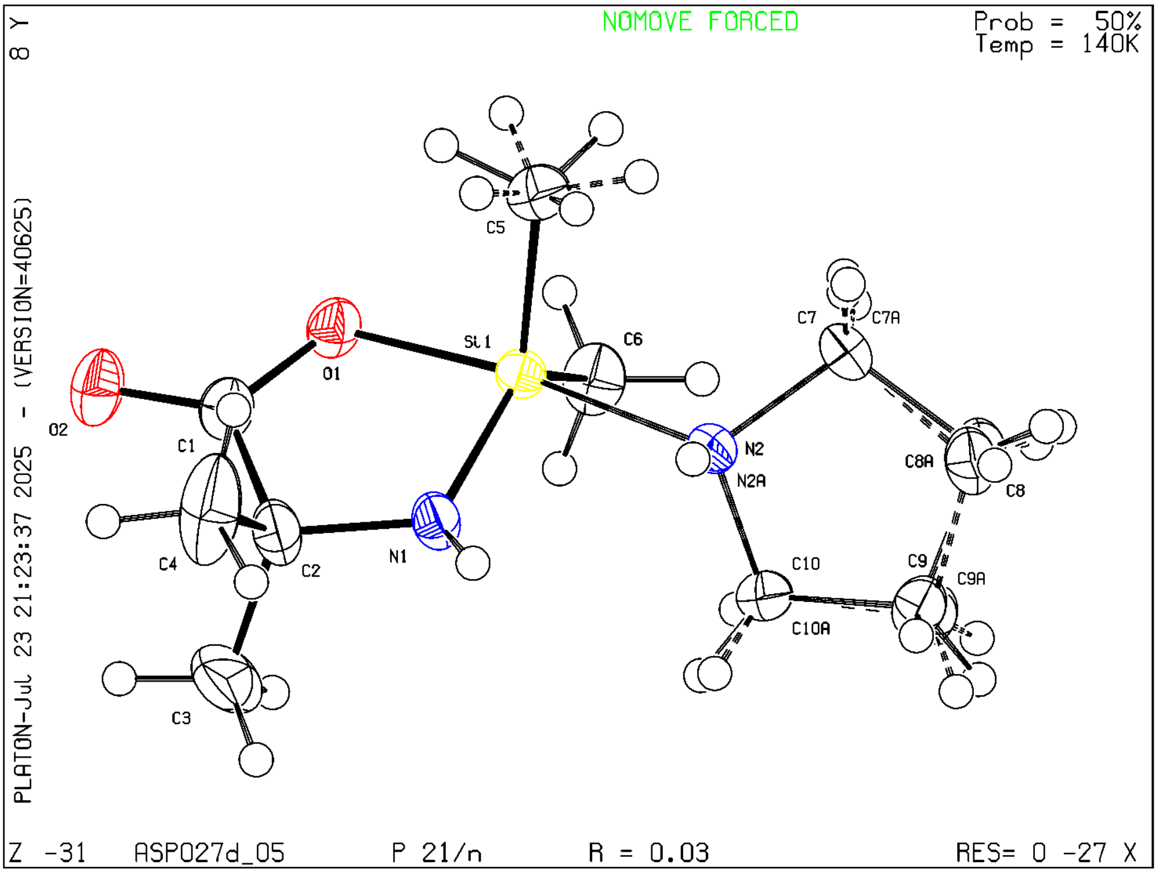

Datablock ASP027dF_01 - ellipsoid plot

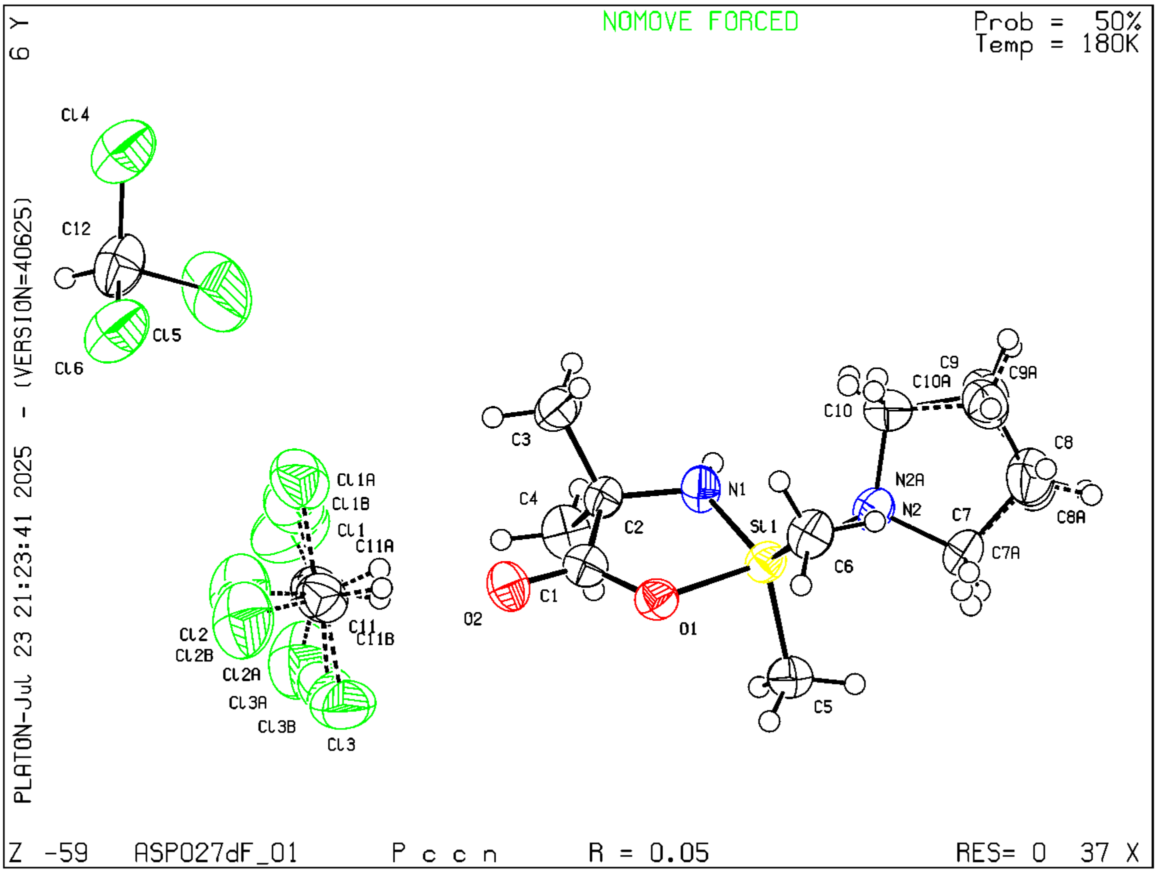

Datablock asp096_01_sq_sq_sq_sq_sq_sq_sq_sq - ellipsoid plot

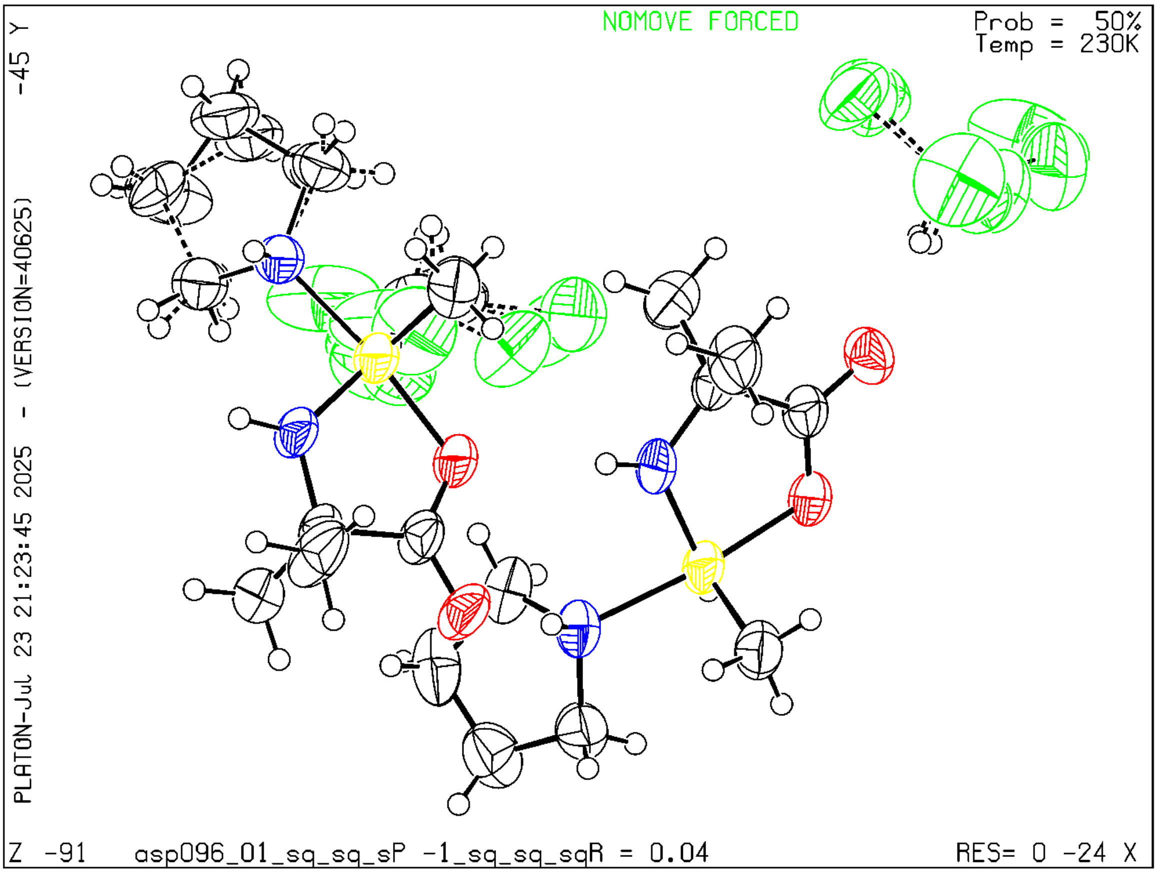

Datablock asp096e_01 - ellipsoid plot

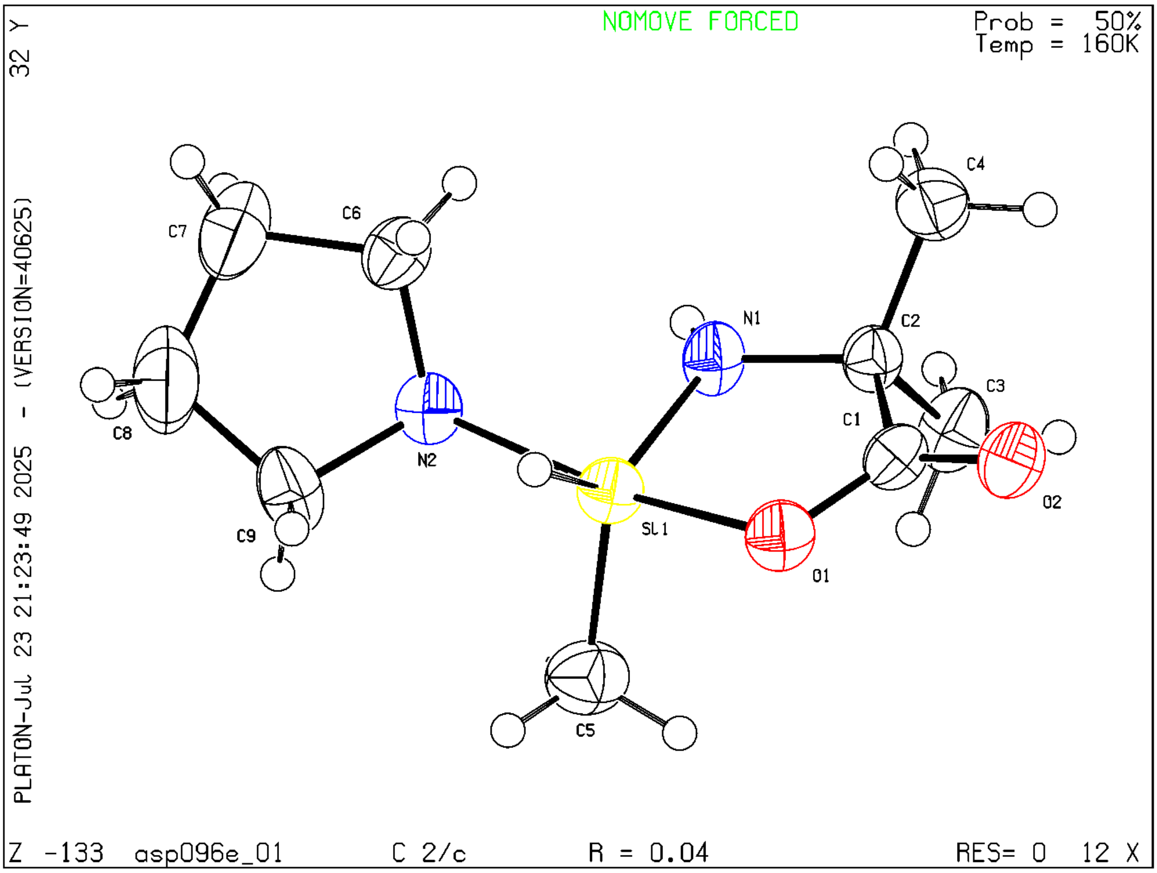

Datablock ASP097_02 - ellipsoid plot

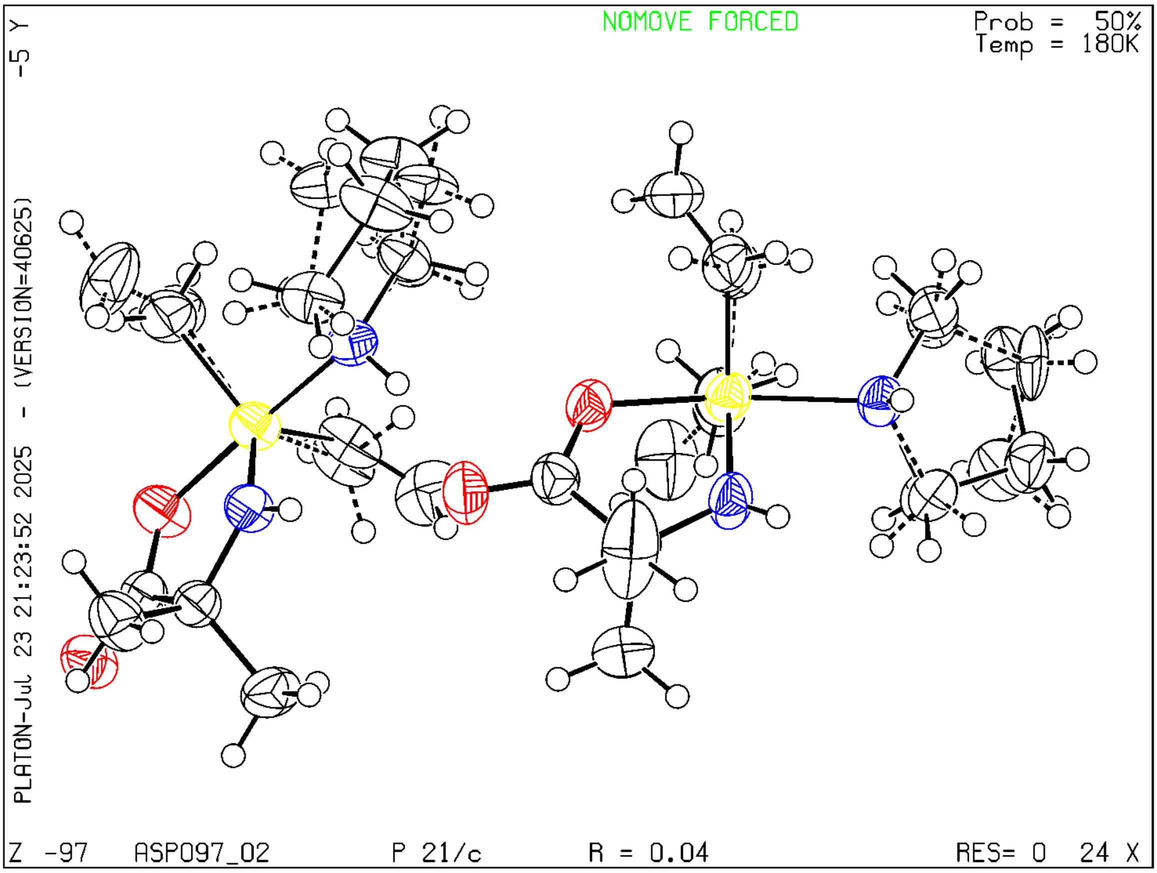

Datablock ASP098_01 - ellipsoid plot

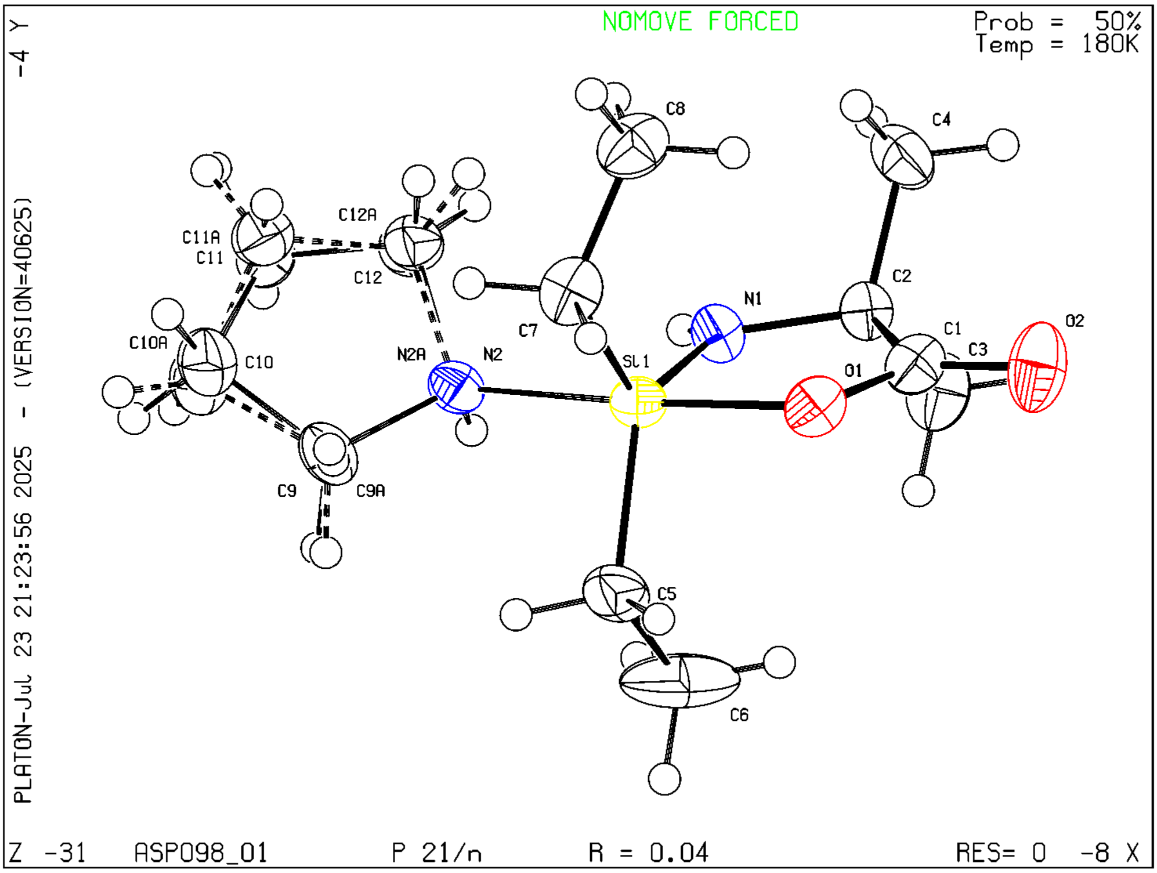

Datablock asp167_01 - ellipsoid plot

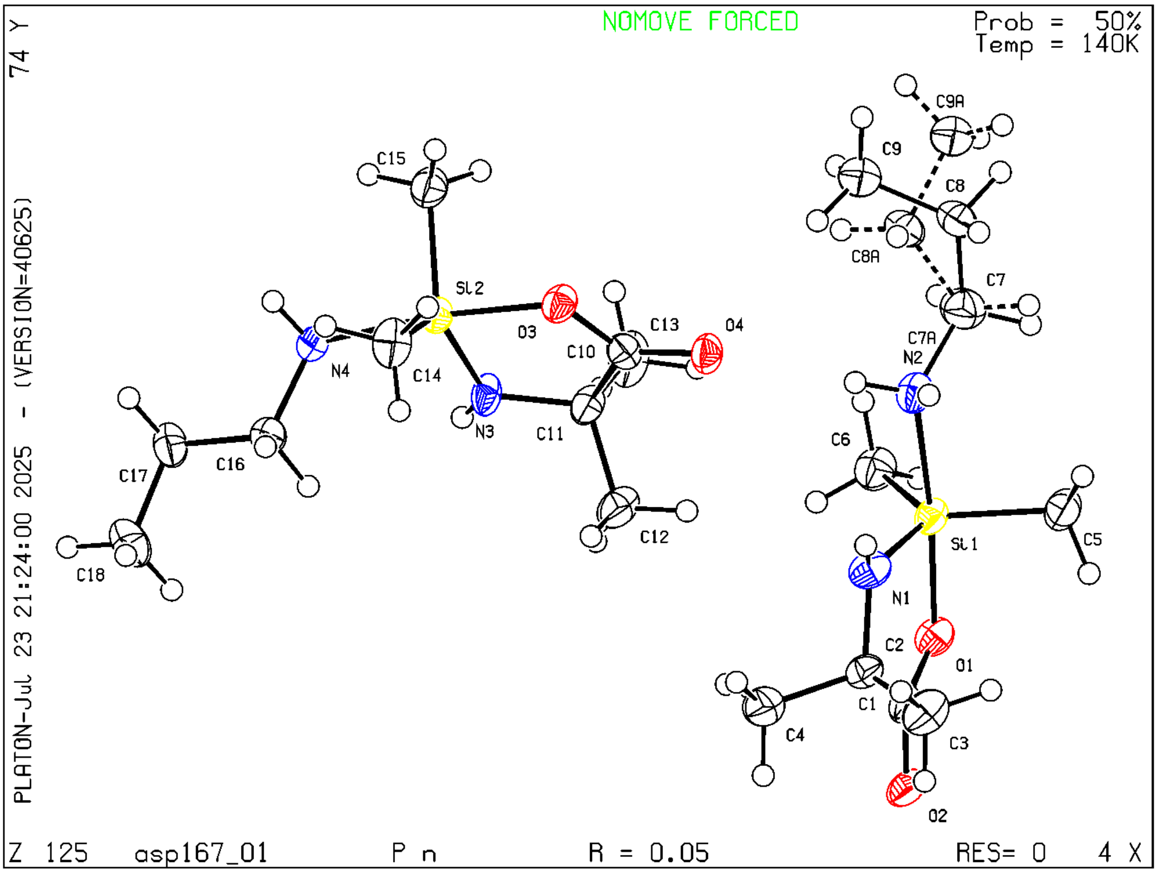

Datablock asp173b_01 - ellipsoid plot

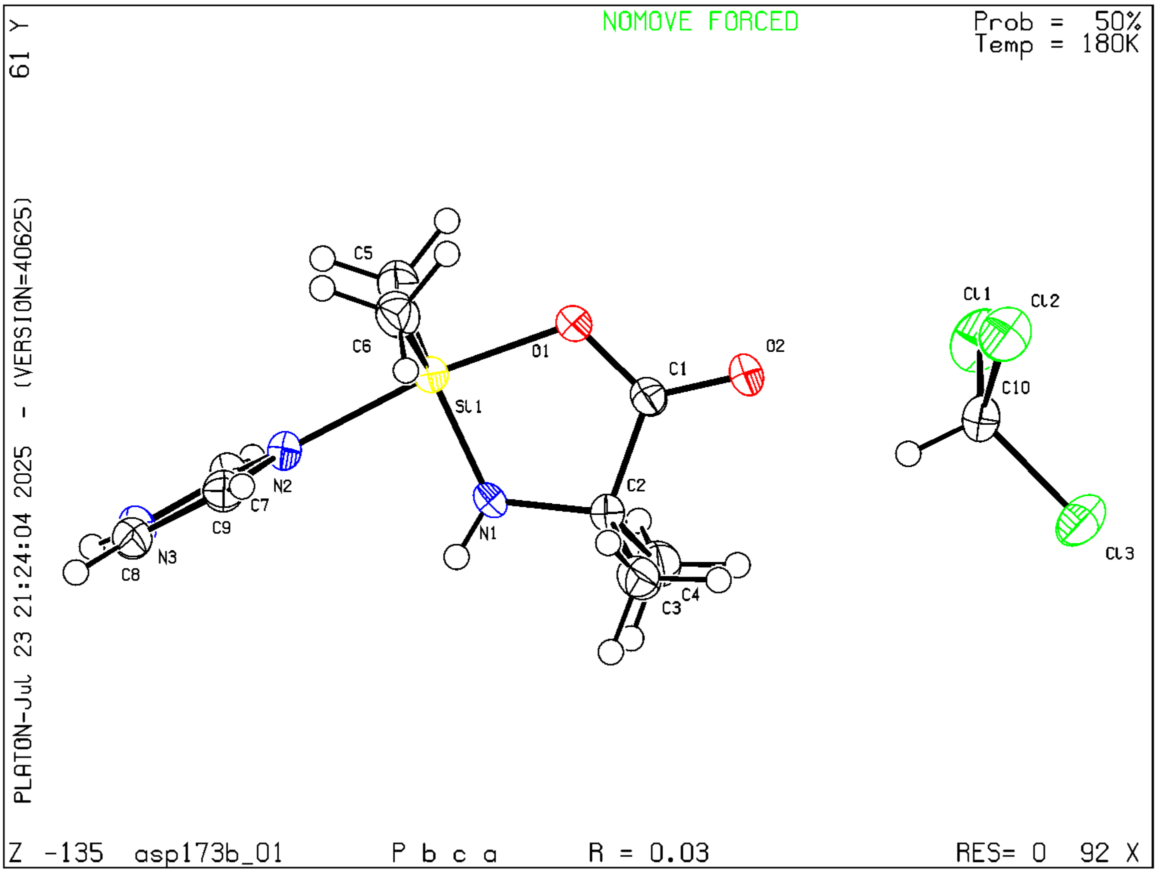

Datablock asp182_01 - ellipsoid plot

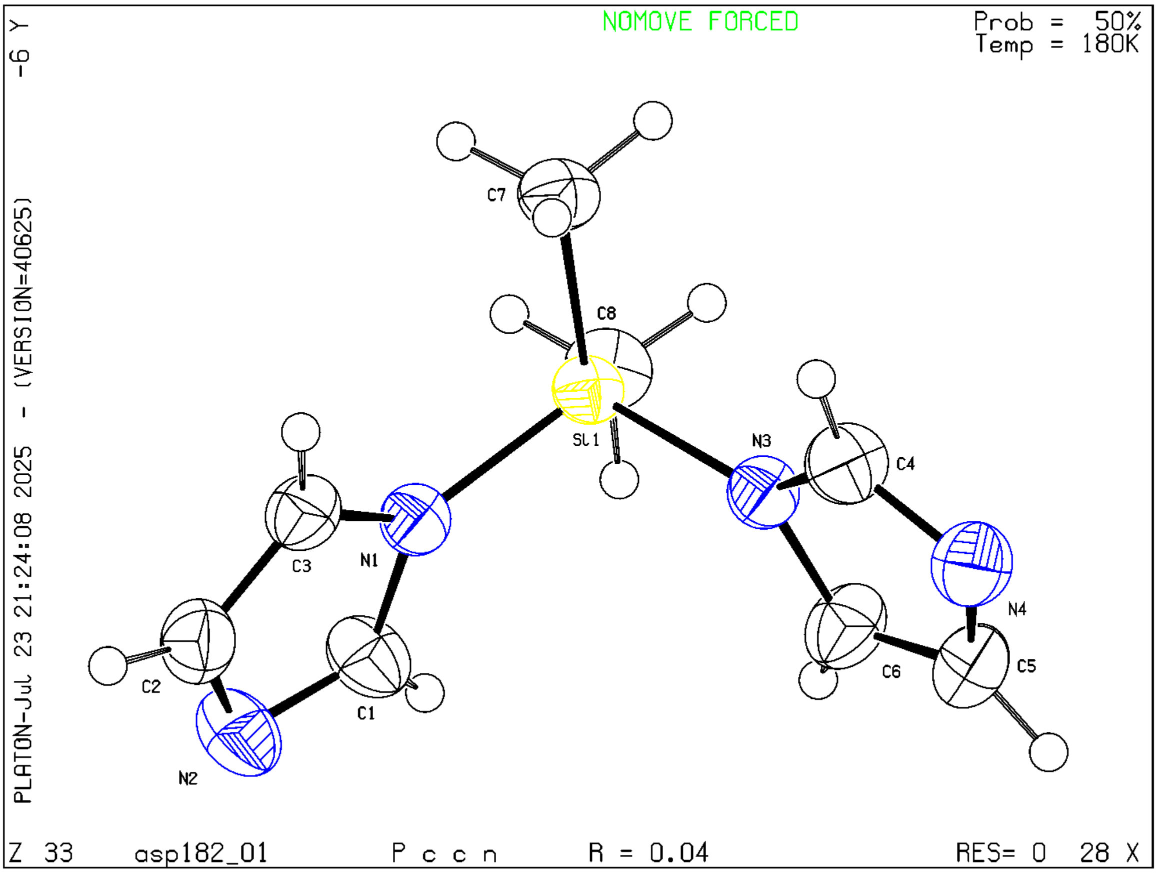

Datablock asp182-st1_01 - ellipsoid plot

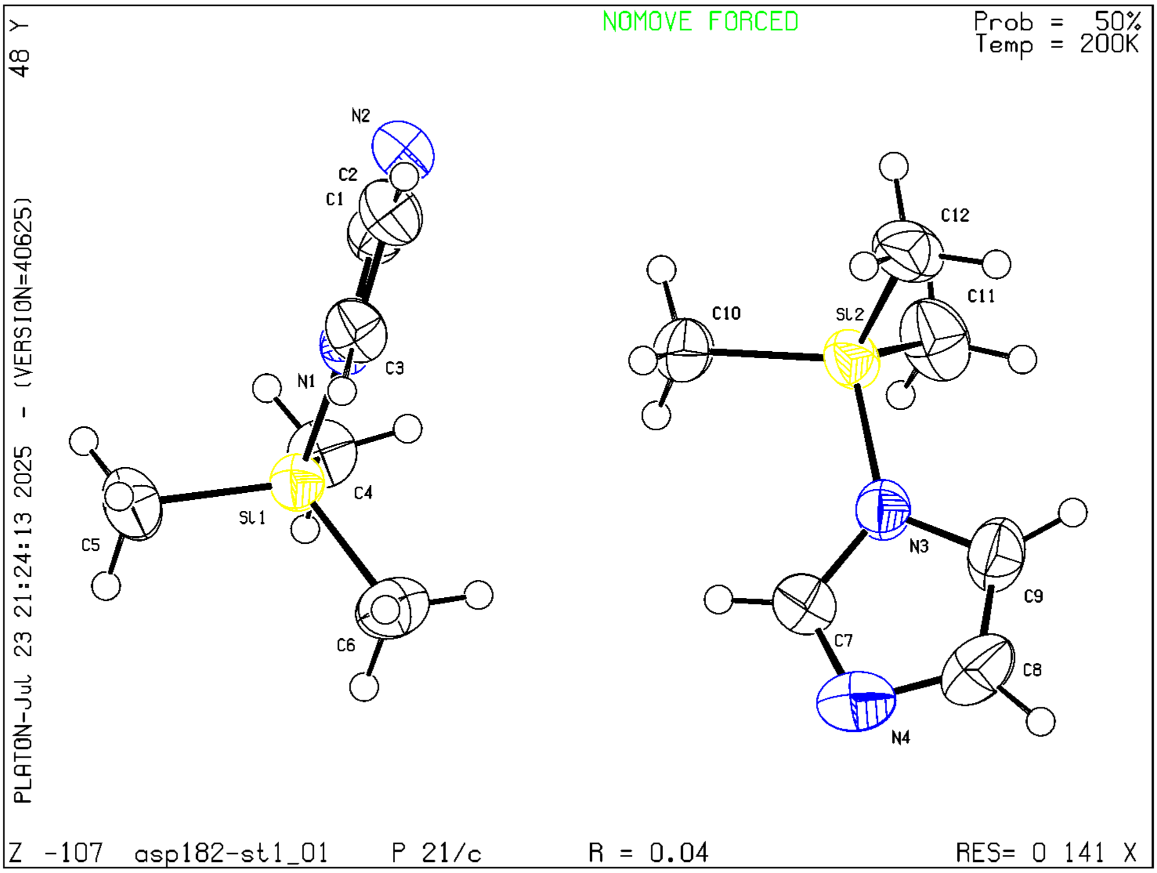

Datablock exp_54_at - ellipsoid plot

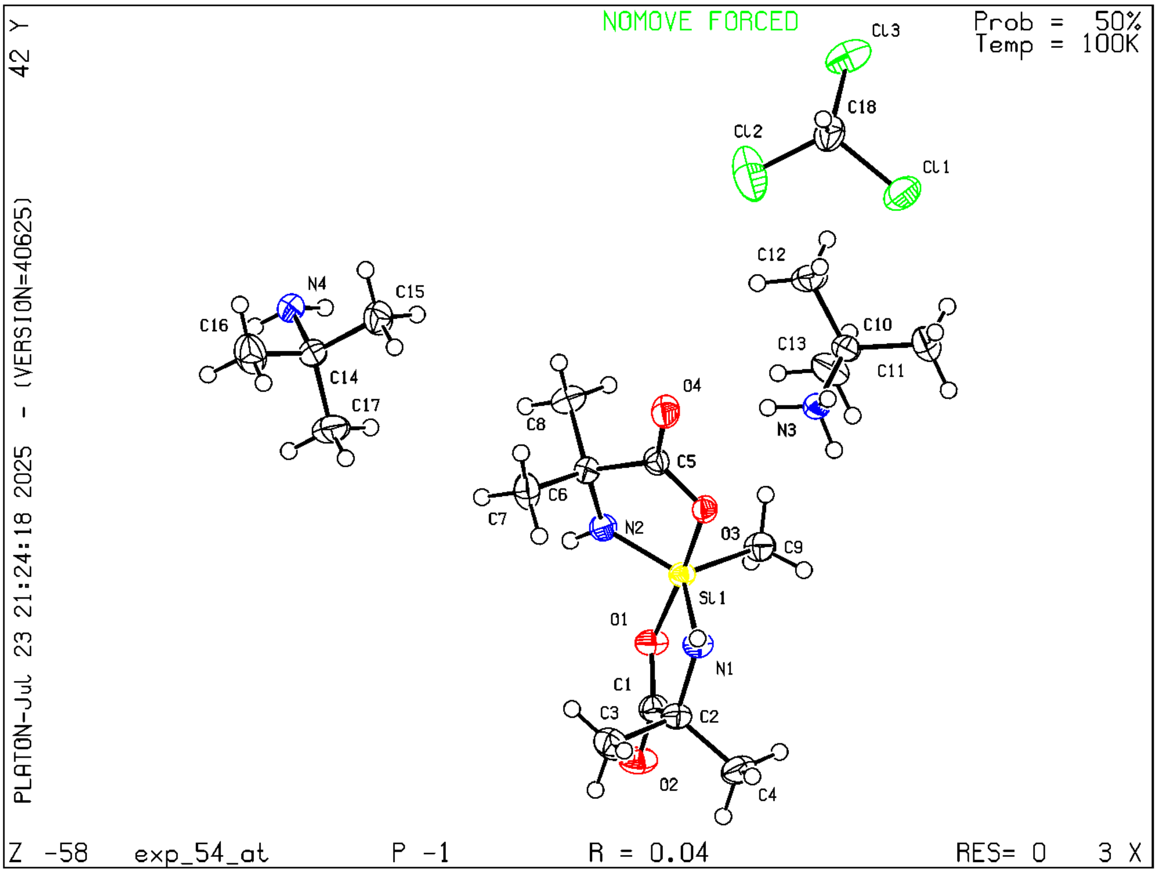

Supplement: Supplementary file 1 [file molecules-30-03501-s001.zip › checkcif_AmineAdducts_all-in-one.pdf]
