# Supplementary material for: Lewis Acid–Base Adducts of α-Amino Isobutyric Acid-Derived Silaheterocycles and Amines
Source: Molecules. 2025 Aug 26;30(17):3501. doi: 10.3390/molecules30173501 (PMC12430707; doi:10.3390/molecules30173501)
Supplement: Supplementary file 1 [file molecules-30-03501-s001.zip › Supporting_information_AmineAdducts_R1_JW.pdf]

Lewis Acid–Base Adducts of α-Amino Isobutyric Acid-Derived Silaheterocycles and Amines

Anne Seidel [1], Erica Brendler [2], Ana Torvisco [3], Roland Fischer [3] and Jörg Wagler [1,]*

[1] Technische Universität Bergakademie Freiberg, Institut für Anorganische Chemie, Leipziger Straße 29, 09596 Freiberg, Germany

[2] Technische Universität Bergakademie Freiberg, Institut für Analytische Chemie, Leipziger Straße 29, 09596 Freiberg, Germany

[3] Technische Universität Graz, Institut für Anorganische Chemie, Stremayrgasse 9/IV, 8010 Graz, Austria

Supporting Information:

Content:

- $^{13}C\{^1H\}$ and $^{29}Si\{^1H\}$ CP/MAS NMR spectra of (Aib)SiMe$_2$(HIm)·CHCl$_3$, (Aib)SiMe$_2$(H$_2$N*n*Pr), (Aib)SiMe$_2$(HPyr), (Aib)SiMeH(HPyr), (Aib)SiMeVi(HPyr) and (Aib)SiEt$_2$(HPyr).
- ^1H, $^{13}C\{^1H\}$ and $^{29}Si\{^1H\}$ NMR spectra of [*t*BuNH$_3$][(Aib)$_2$SiMe].
- IR spectra of (Aib)SiMe$_2$(HIm)·CHCl$_3$, (Aib)SiMe$_2$(H$_2$N*n*Pr), (Aib)SiMe$_2$(HPyr), (Aib)SiMeH(HPyr), (Aib)SiMeVi(HPyr), (Aib)SiEt$_2$(HPyr) and [*t*BuNH$_3$][(Aib)$_2$SiMe].
- Syntheses of SiMe$_3$(Im) and SiMe$_2$(Im)$_2$.
- Comparison of selected parameters of the crystal structure refinement of compound (Aib)SiMe$_2$(HPyr) without and with refinement of the disorder of the pyrrolidine backbone.
- Space Fill view of a molecule of (Aib)SiMe$_2$(H$_2$N*n*Pr).
- Evaluation of the tensors of ^{29}Si Chemical Shift Anisotropy (CSA).

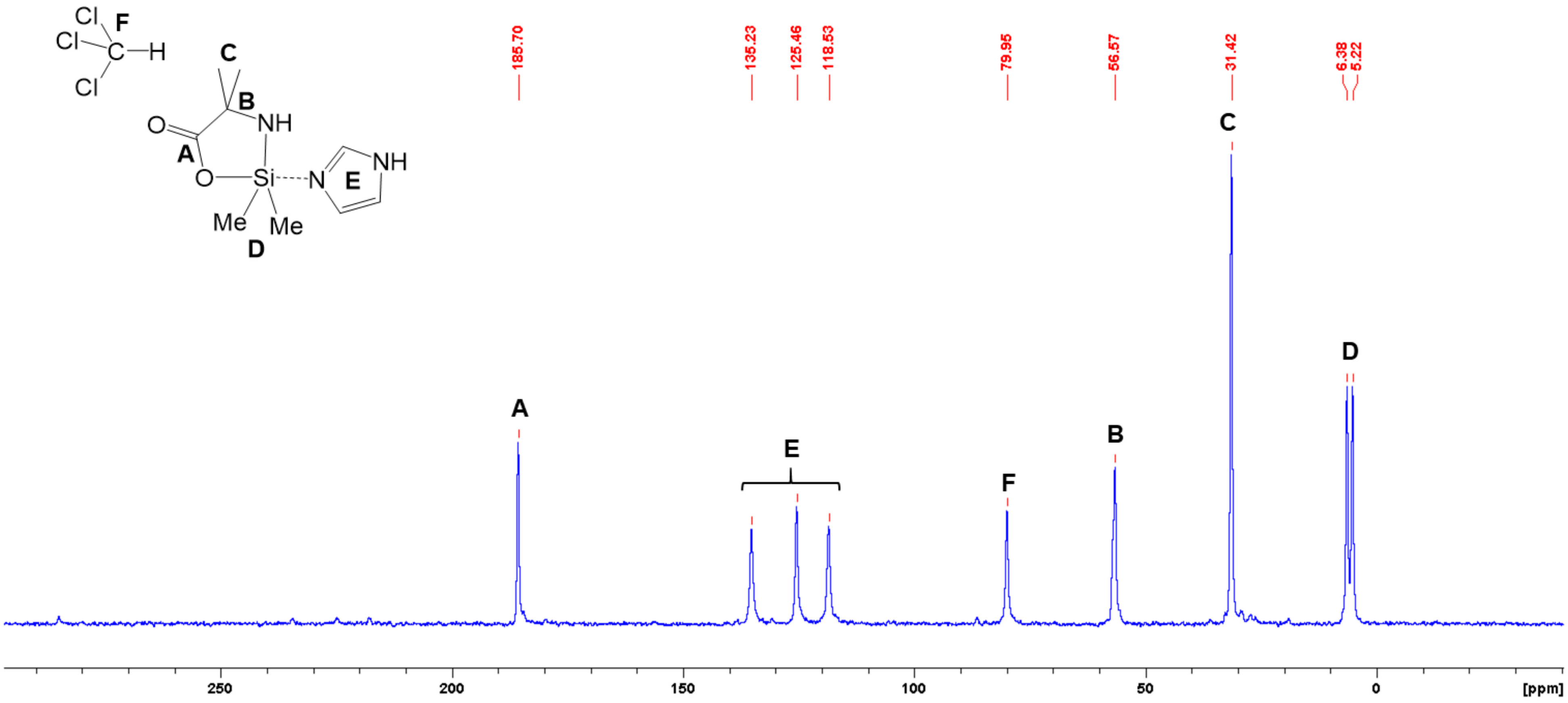

Figure S1. $^{13}C\{^1H\}$ CP/MAS NMR spectrum of (Aib)SiMe$_2$(HIm)·CHCl$_3$ (ν_{rot} = 10 kHz) with assignment of (groups of) signals.

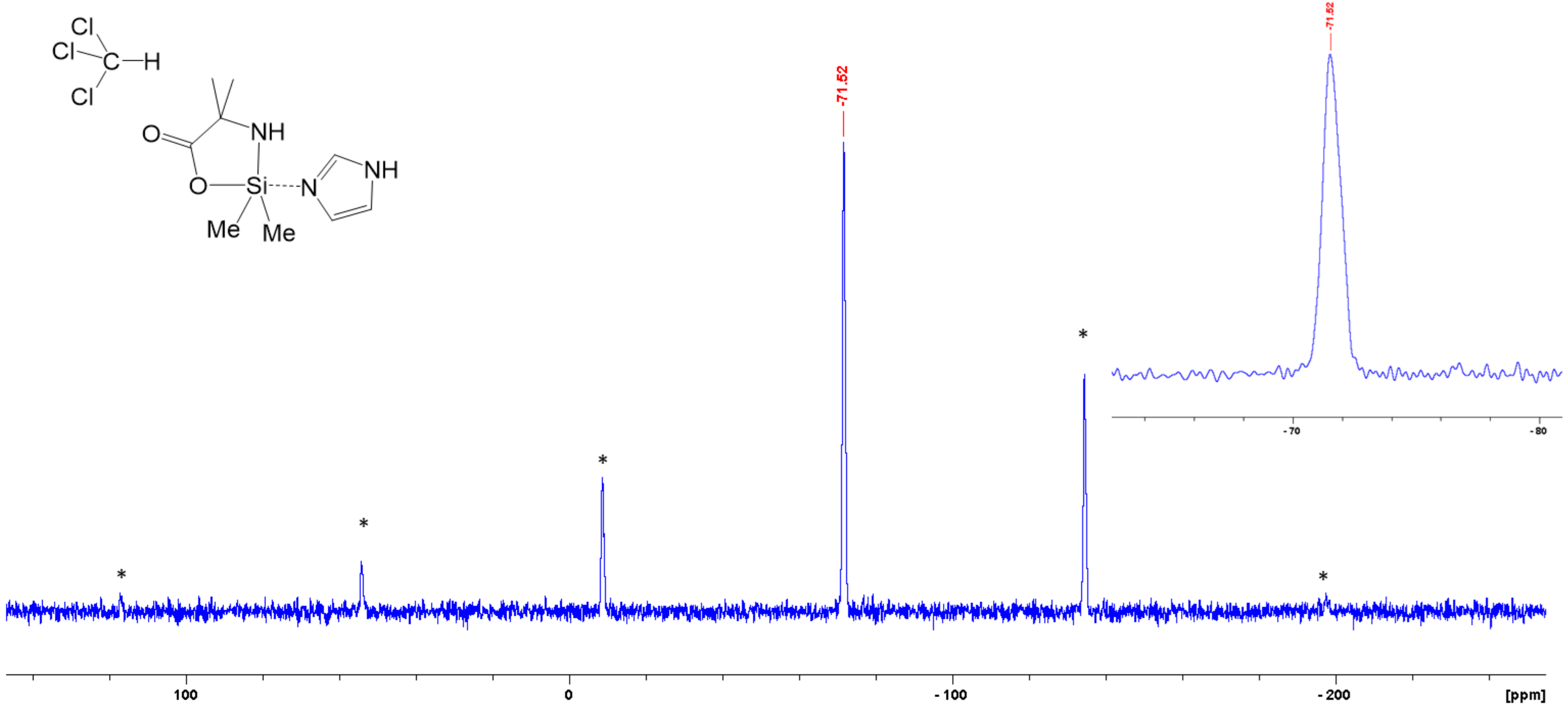

Figure S2. ^{29}Si{^{1}H} CP/MAS NMR spectrum of (Aib)SiMe$_2$(HIm)·CHCl$_3$ (ν_{rot} = 5 kHz, spinning side bands are asterisked*) with a magnification of the isotropic shift signal as an inset.

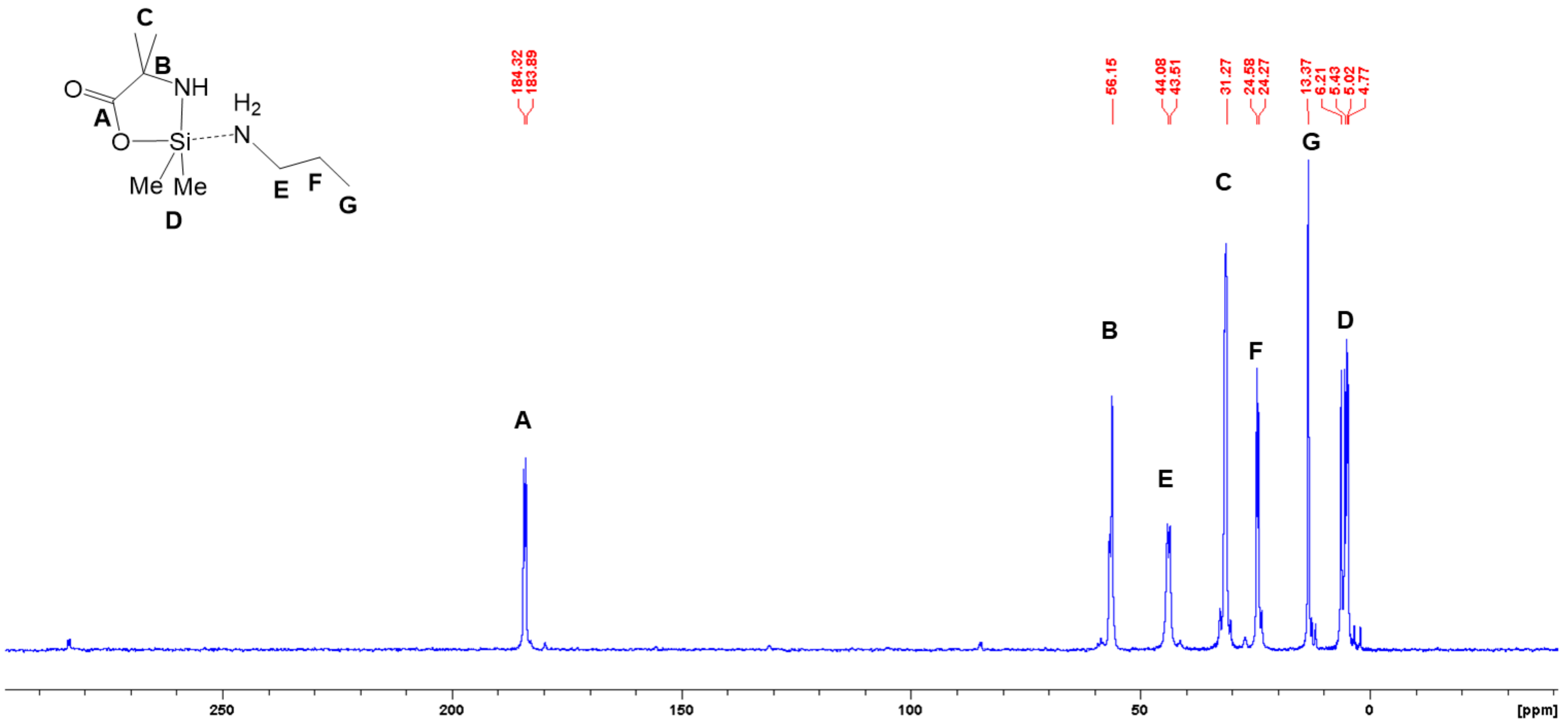

Figure S3. $^{13}C\{^{1}H\}$ CP/MAS NMR spectrum of (Aib)SiMe$_2$(NH$_2$*n*Pr) (ν_{rot} = 10 kHz) with assignment of (groups of) signals.

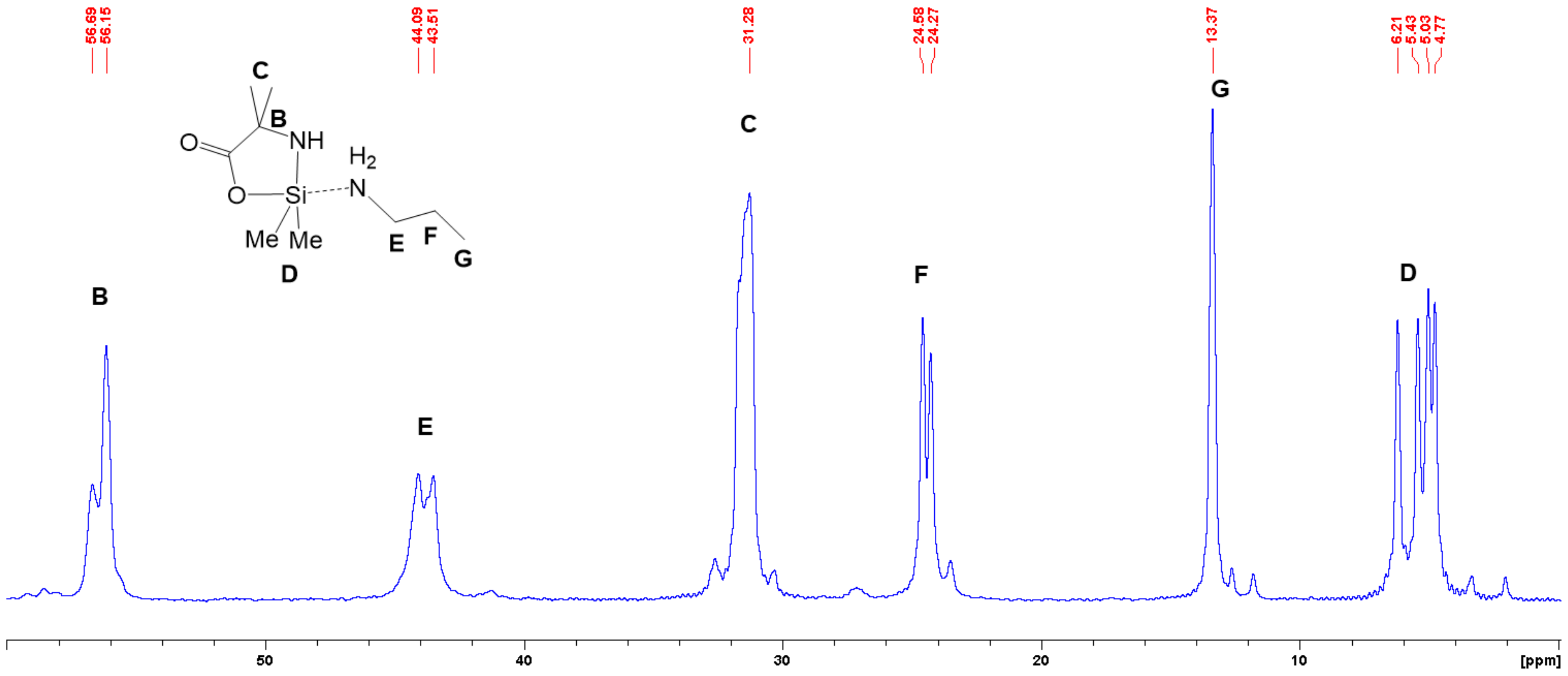

Figure S4. Magnification of the upfield section of the $^{13}C\{^{1}H\}$ CP/MAS NMR spectrum of (Aib)SiMe$_2$(NH$_2$*n*Pr) (ν_{rot} = 10 kHz) (cf. Figure S3) with assignment of (groups of) signals.

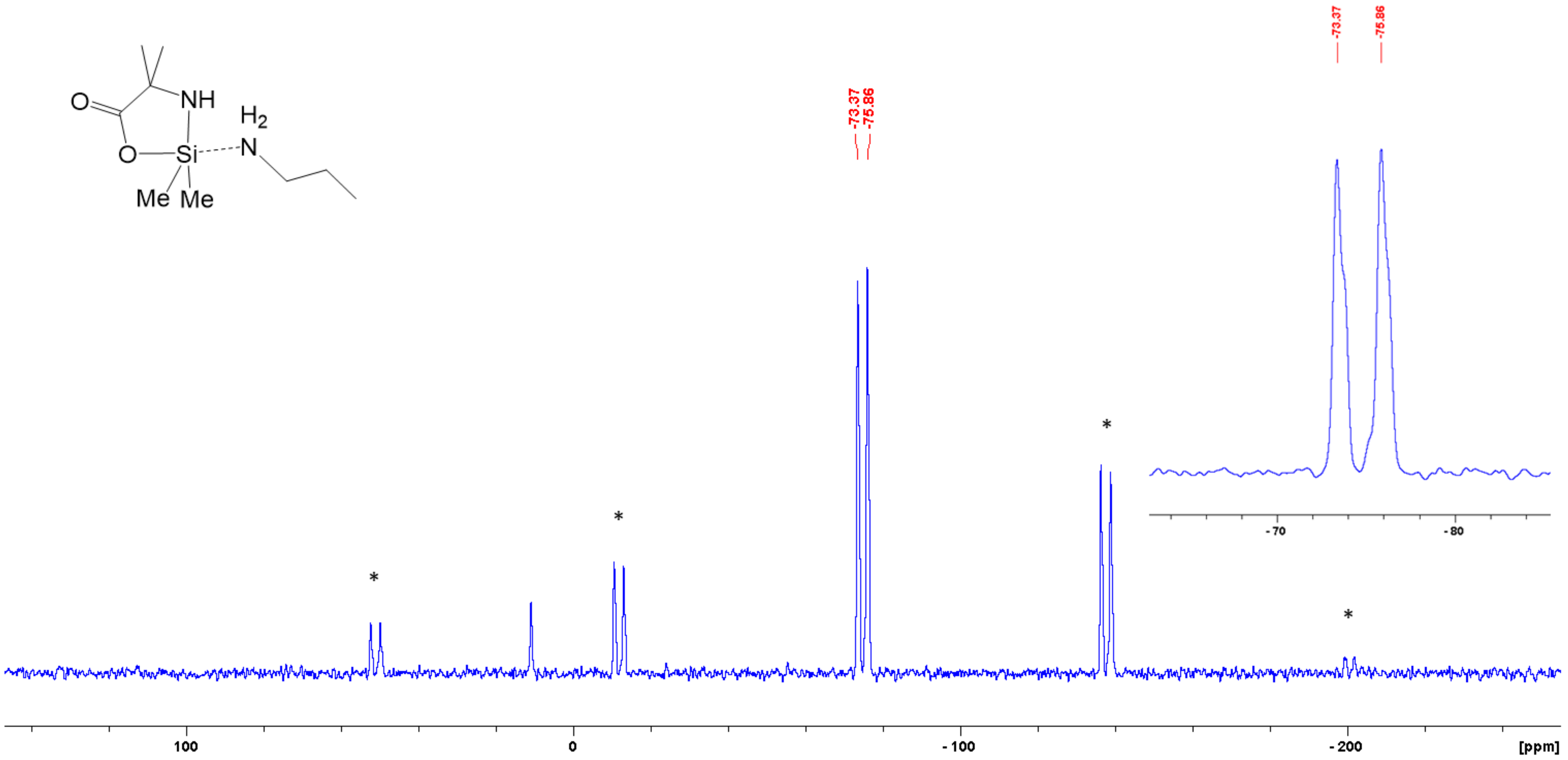

Figure S5. $^{29}Si\{^1H\}$ CP/MAS NMR spectrum of (Aib)SiMe$_2$(NH$_2$*n*Pr) (ν_{rot} = 5 kHz, spinning side bands are asterisked*) with a magnification of the isotropic shift signals as an inset.

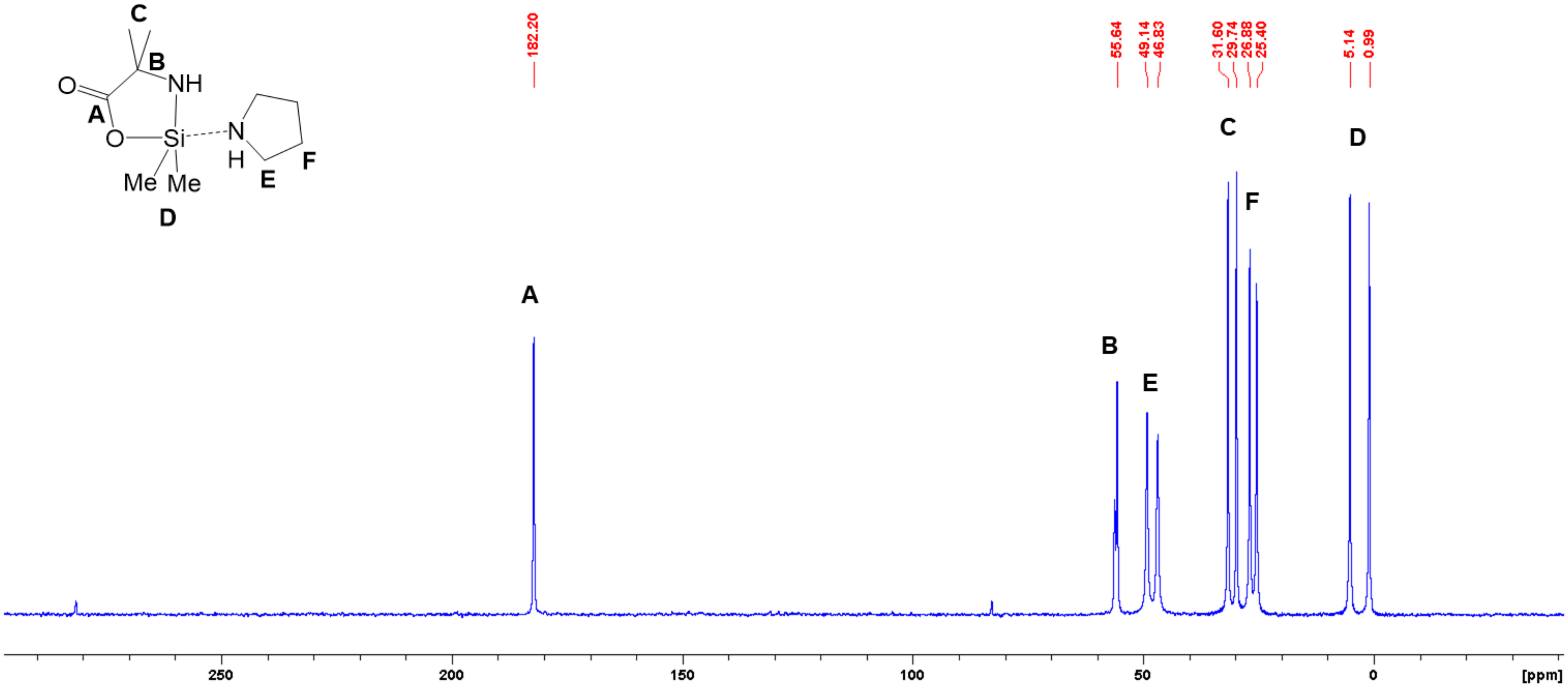

Figure S6. $^{13}C\{^1H\}$ CP/MAS NMR spectrum of (Aib)SiMe$_2$(HPyr) (ν_{rot} = 10 kHz) with assignment of (groups of) signals.

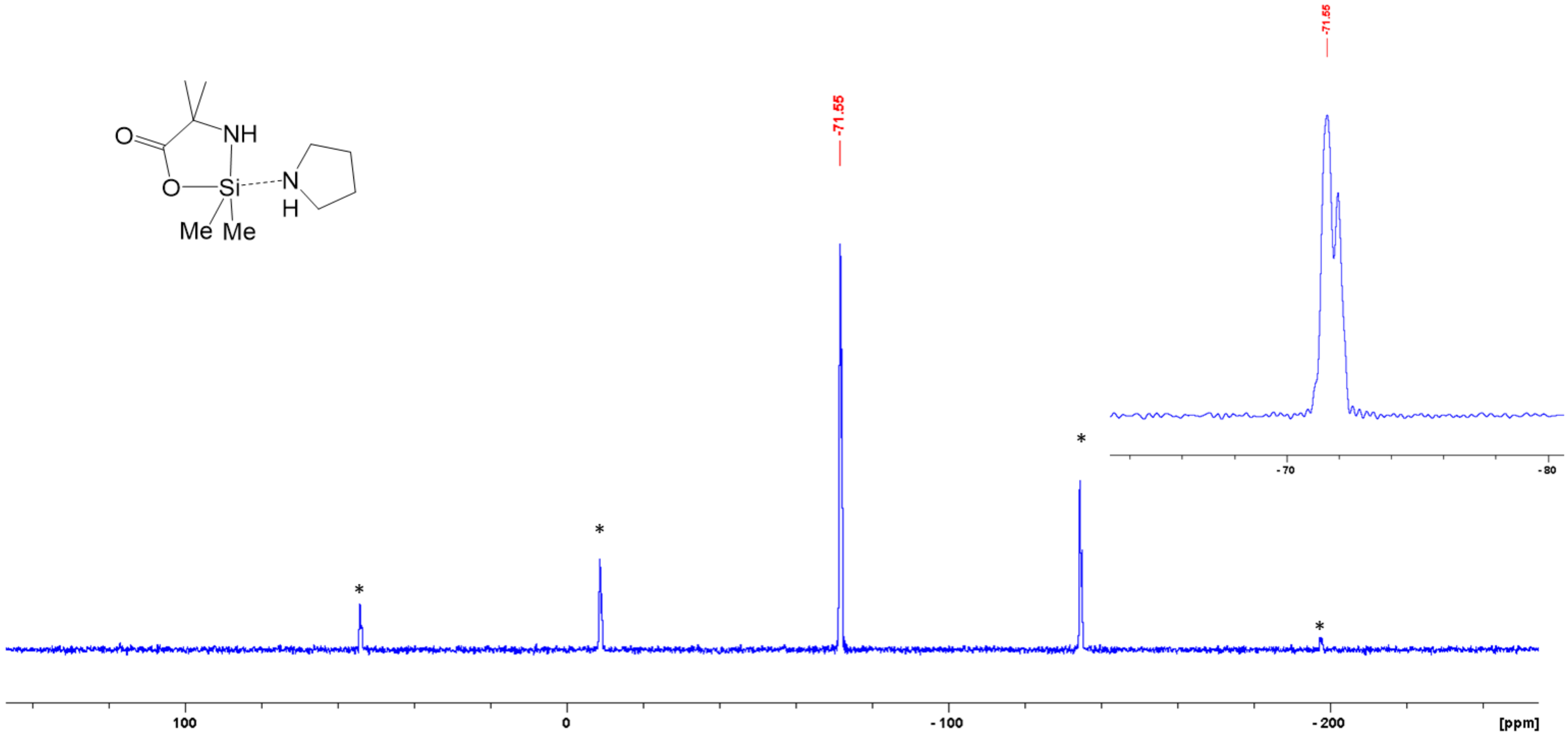

Figure S7. $^{29}Si\{^1H\}$ CP/MAS NMR spectrum of (Aib)SiMe$_2$(HPyr) (ν_{rot} = 5 kHz, spinning side bands are asterisked*) with a magnification of the isotropic shift signal as an inset.

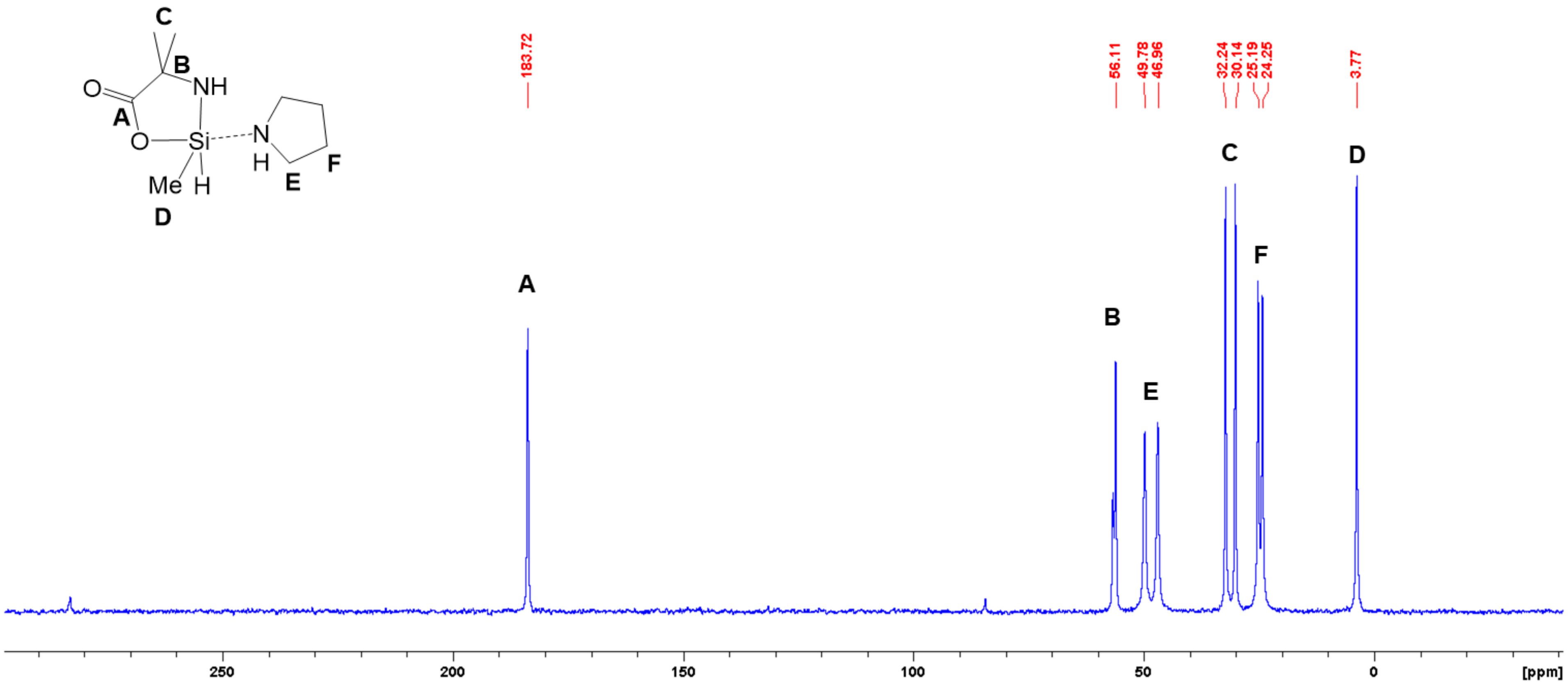

Figure S8. $^{13}C\{^1H\}$ CP/MAS NMR spectrum of (Aib)SiMeH(HPyr) (ν_{rot} = 10 kHz) with assignment of (groups of) signals.

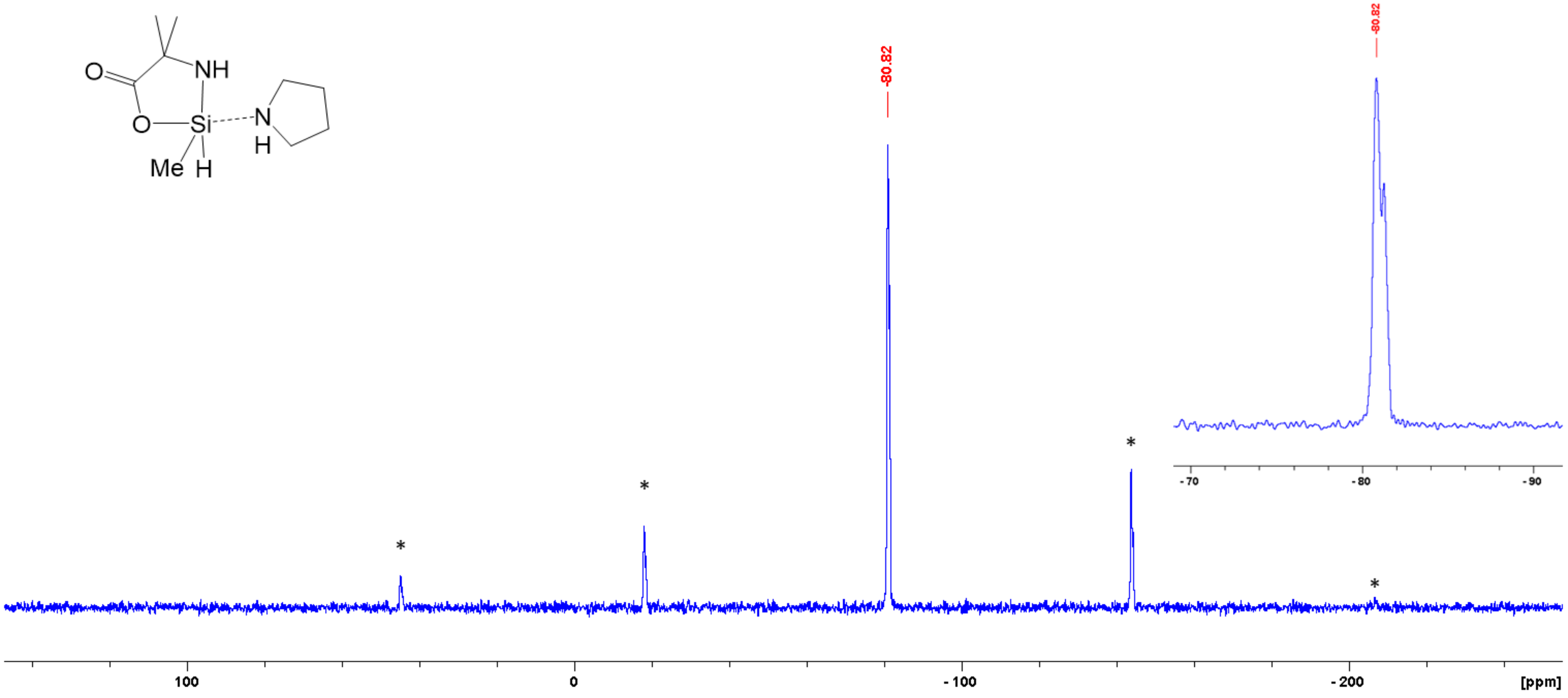

Figure S9. $^{29}Si\{^1H\}$ CP/MAS NMR spectrum of (Aib)SiMeH(HPyr) (ν_{rot} = 5 kHz, spinning side bands are asterisked*) with a magnification of the isotropic shift signal as an inset.

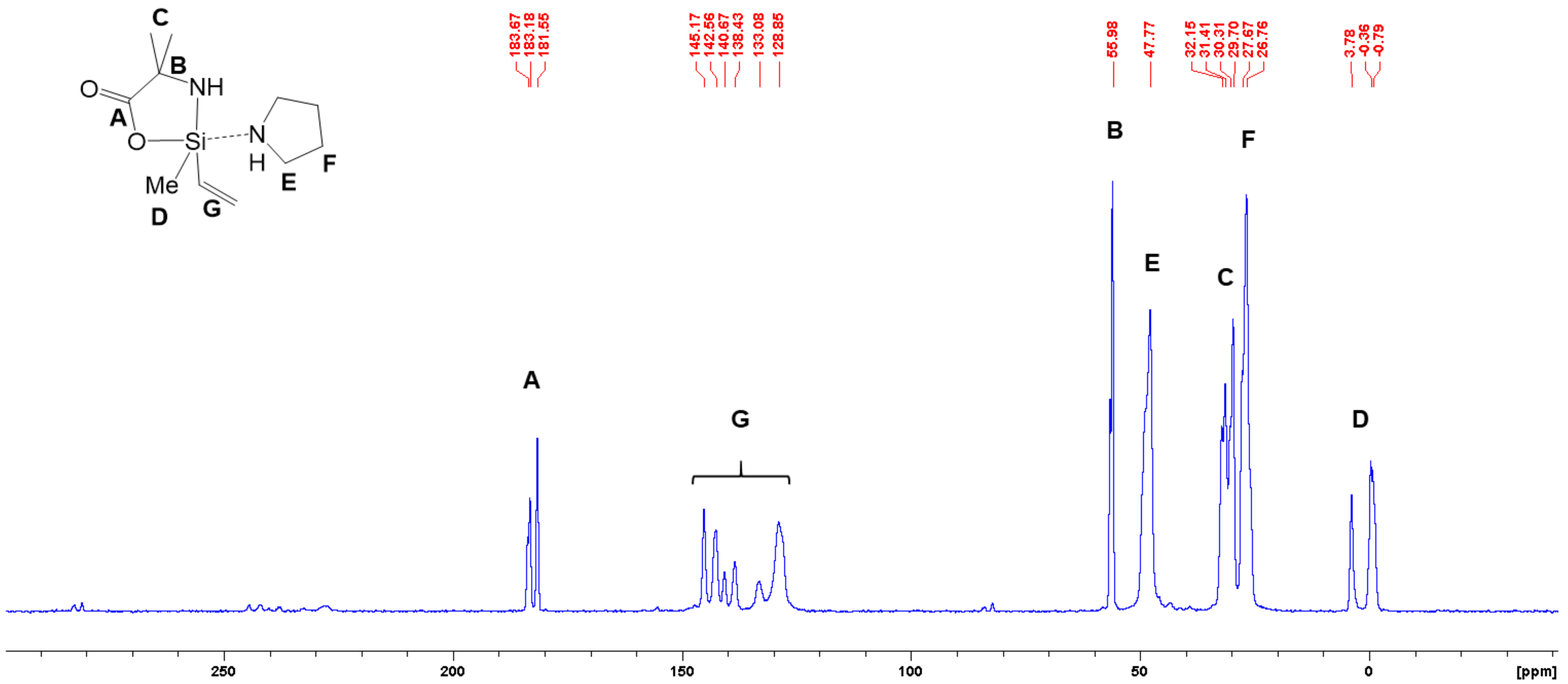

Figure S10. $^{13}C\{^1H\}$ CP/MAS NMR spectrum of (Aib)SiMeVi(HPyr) (ν_{rot} = 10 kHz) with assignment of (groups of) signals.

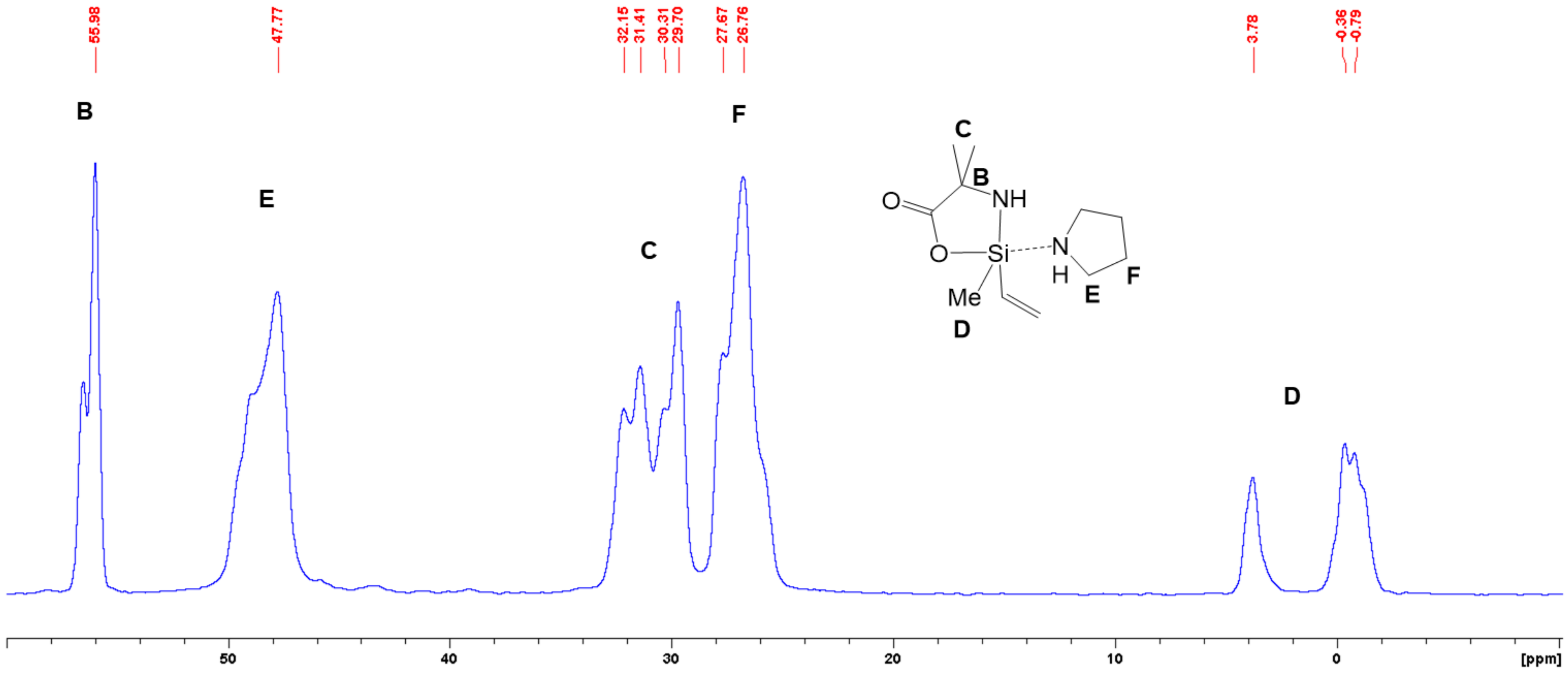

Figure S11. Magnification of the upfield section of the $^{13}C\{^{1}H\}$ CP/MAS NMR spectrum of (Aib)SiMeVi(HPyr) (ν_{rot} = 10 kHz) (cf. Figure S10) with assignment of (groups of) signals.

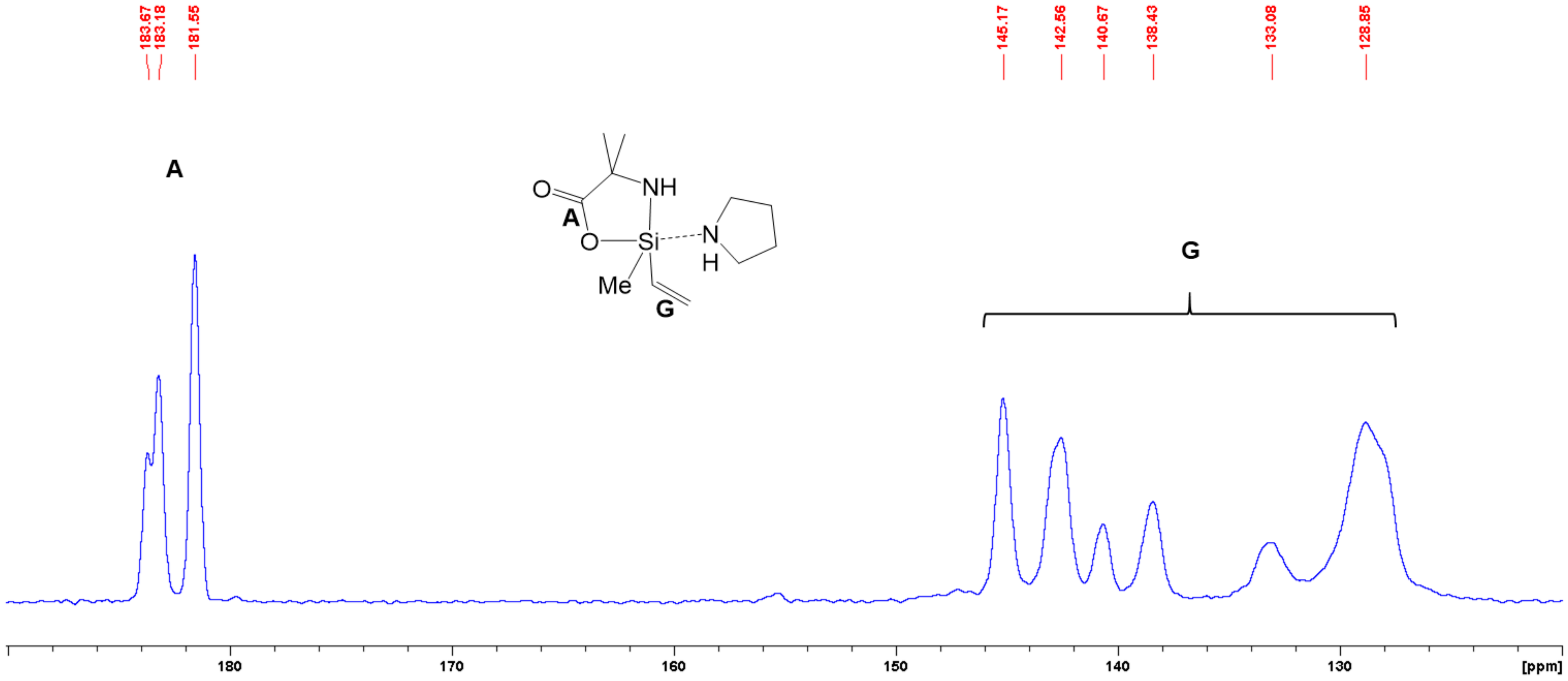

Figure S12. Magnification of the downfield section of the $^{13}C\{^{1}H\}$ CP/MAS NMR spectrum of (Aib)SiMeVi(HPyr) (ν_{rot} = 10 kHz) (cf. Figure S10) with assignment of (groups of) signals.

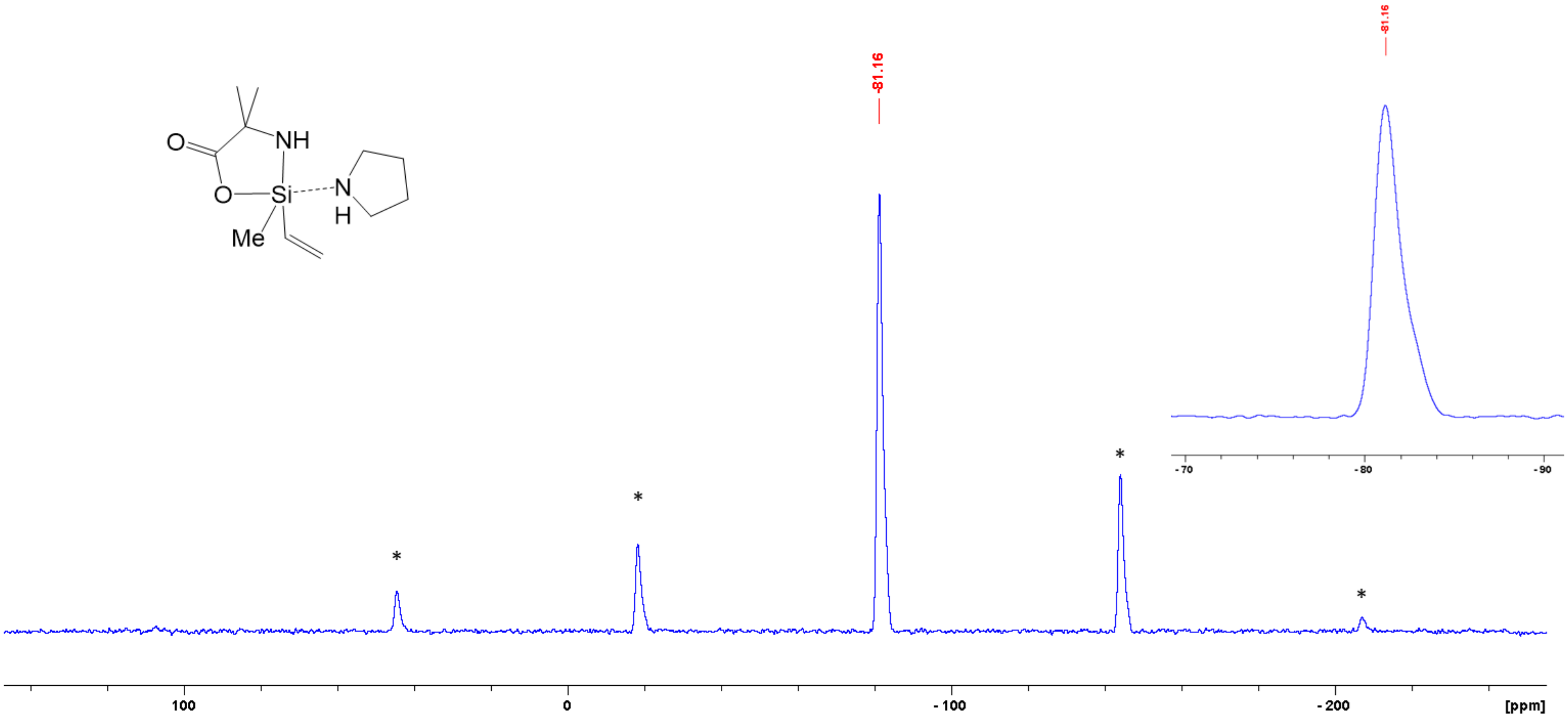

Figure S13. $^{29}Si\{^1H\}$ CP/MAS NMR spectrum of (Aib)SiMeVi(HPyr) (ν_{rot} = 5 kHz, spinning side bands are asterisked*) with a magnification of the isotropic shift signal as an inset.

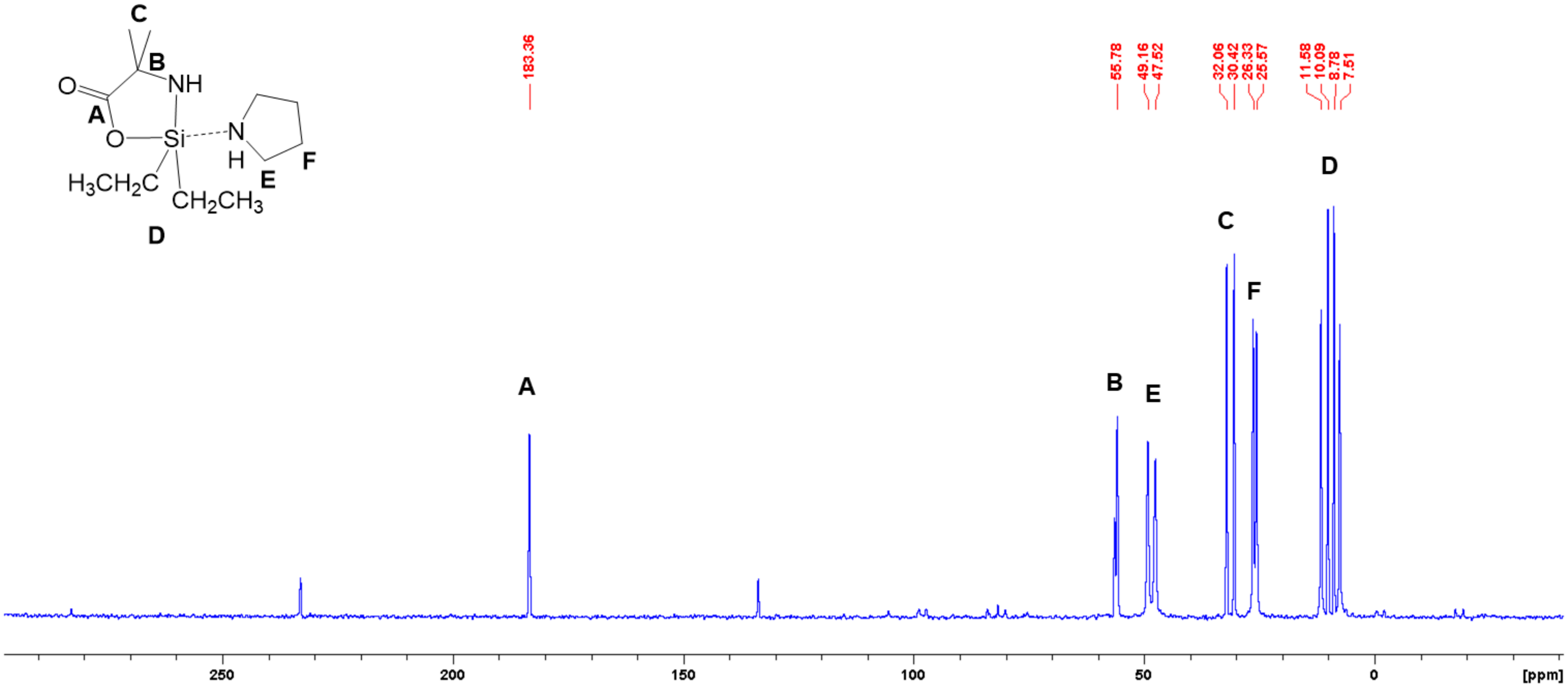

Figure S14. $^{13}C\{^1H\}$ CP/MAS NMR spectrum of (Aib)SiEt$_2$(HPyr) (ν_{rot} = 5 kHz) with assignment of (groups of) signals.

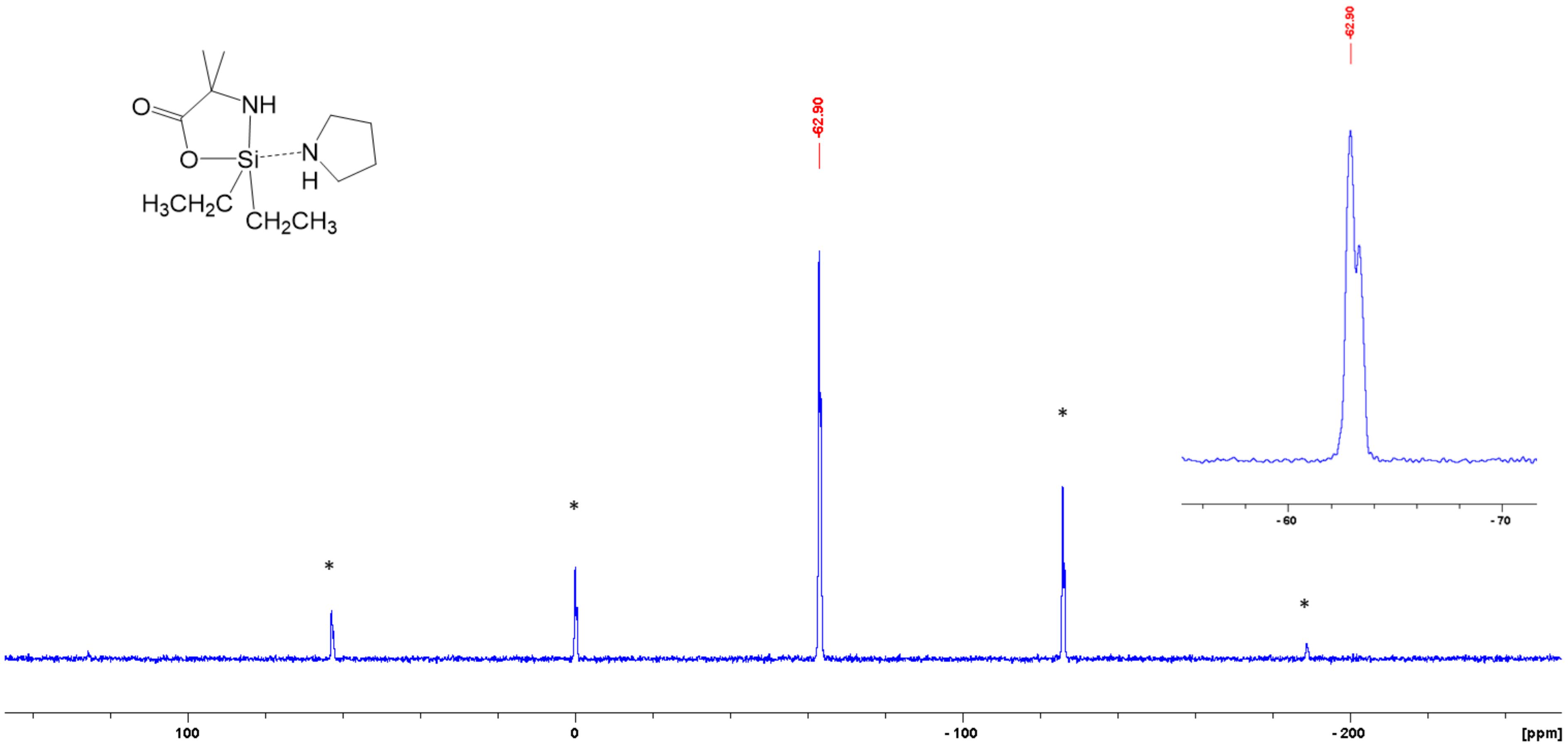

Figure S15. $^{29}Si\{^{1}H\}$ CP/MAS NMR spectrum of (Aib)SiEt$_2$(HPyr) (ν_{rot} = 5 kHz, spinning side bands are asterisked*) with a magnification of the isotropic shift signal as an inset.

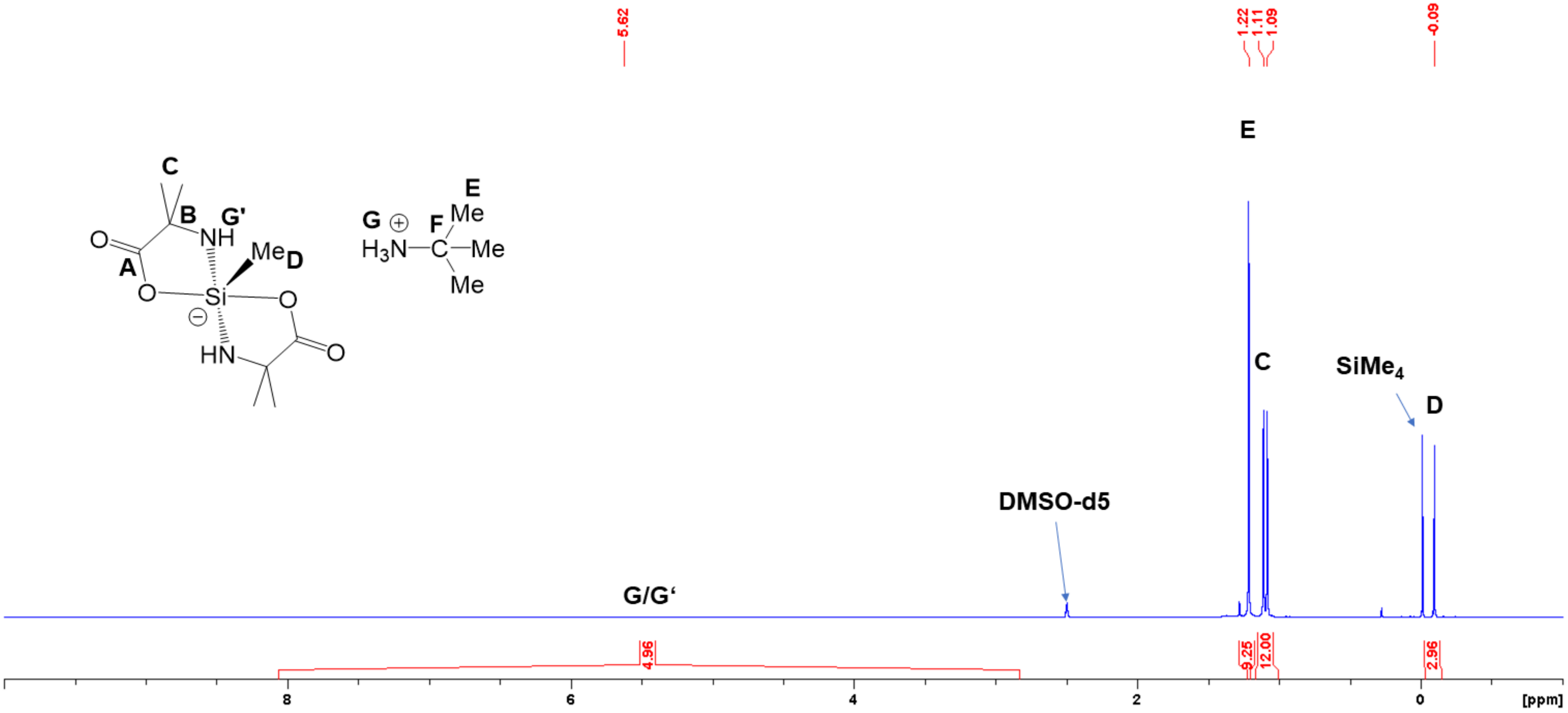

Figure S16. ^{1}H NMR spectrum (DMSO-d6) of [*t*BuNH$_3$][(Aib)$_2$SiMe].

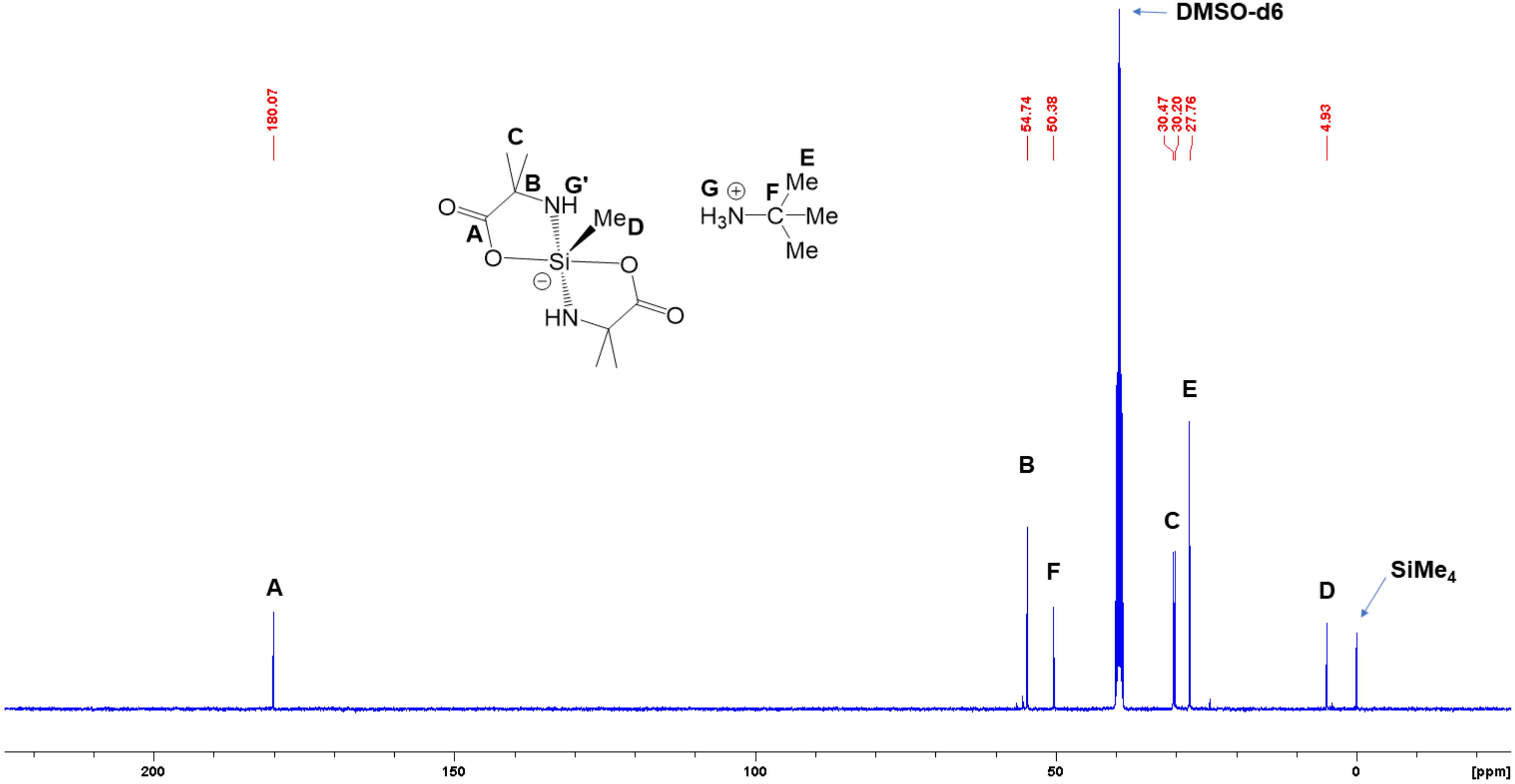

Figure S17. $^{13}C\{^{1}H\}$ NMR spectrum (DMSO-d6) of [*t*BuNH$_3$][(Aib)$_2$SiMe].

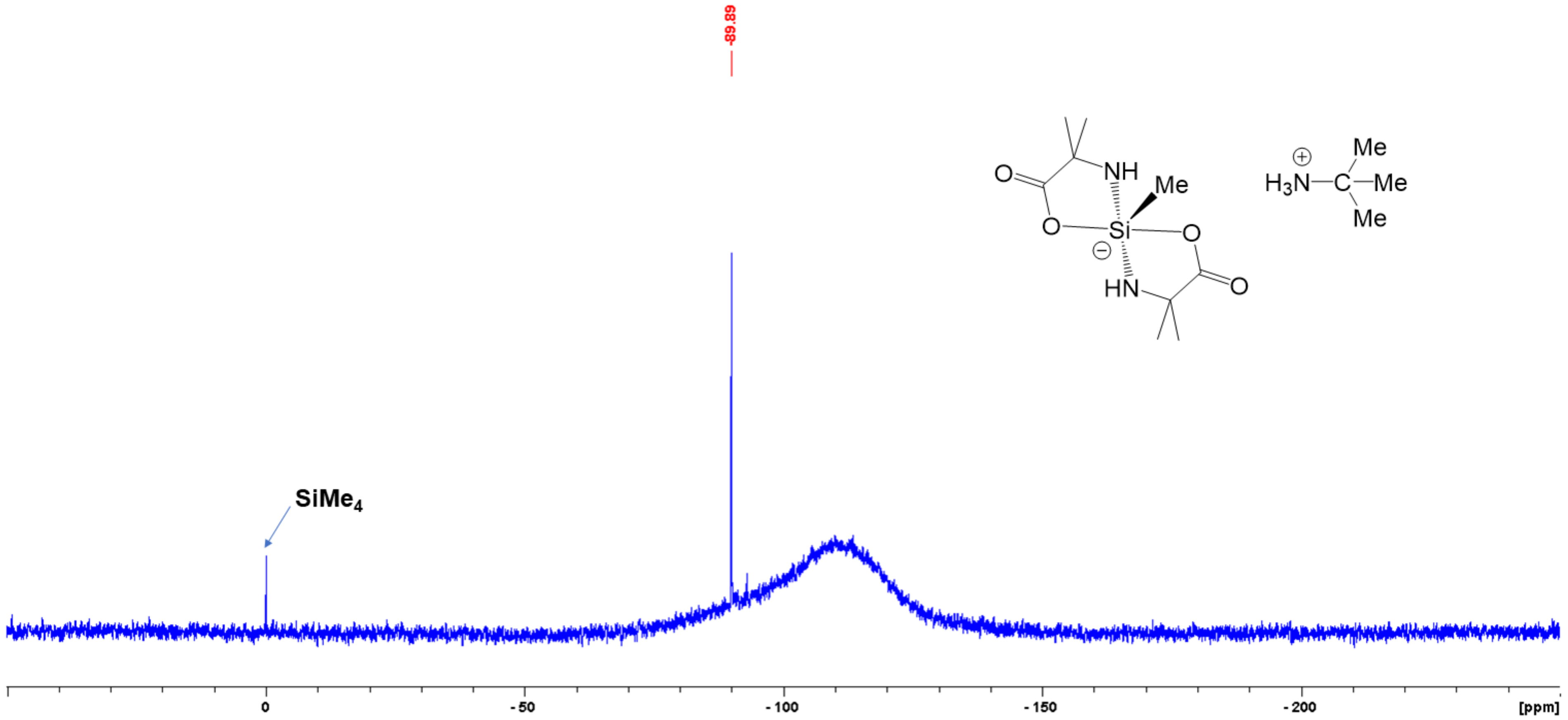

Figure S18. $^{29}Si\{^{1}H\}$ NMR spectrum (DMSO-d6) of [*t*BuNH$_3$][(Aib)$_2$SiMe].

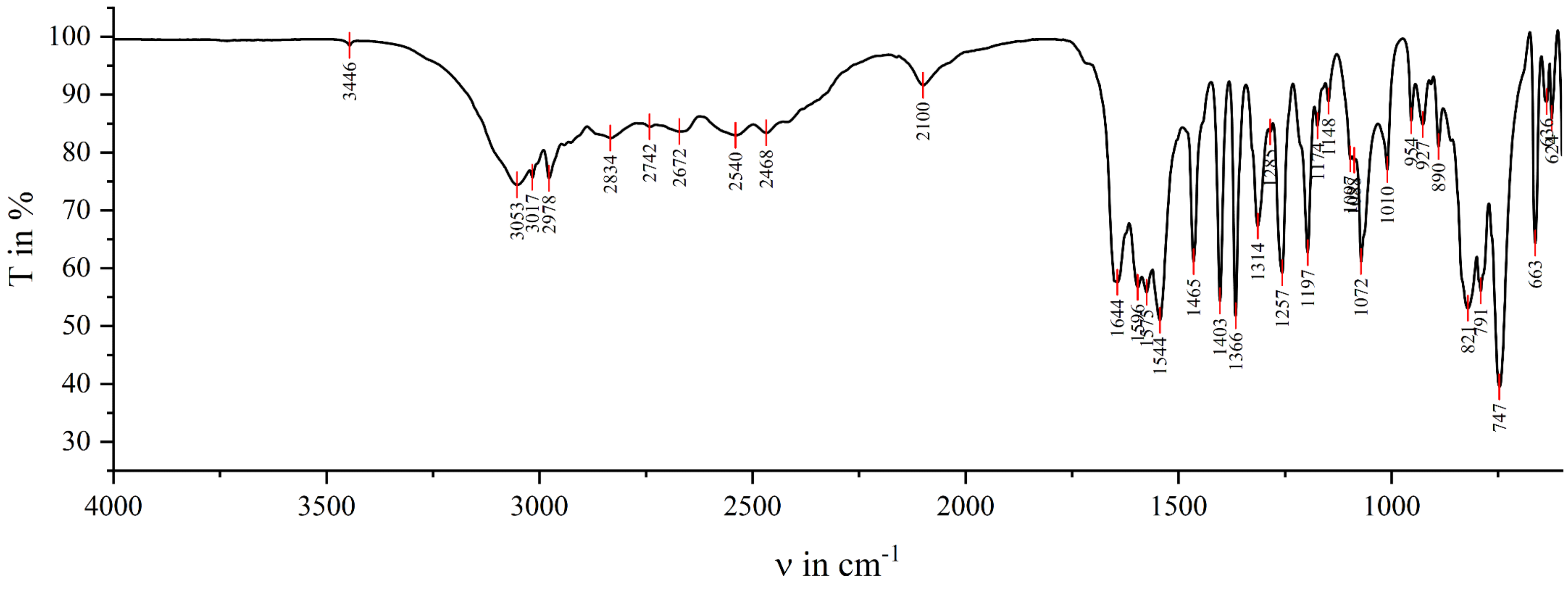

Figure S19. IR (ATR) spectrum of (Aib)SiMe$_2$(HIm)·(CHCl$_3$).

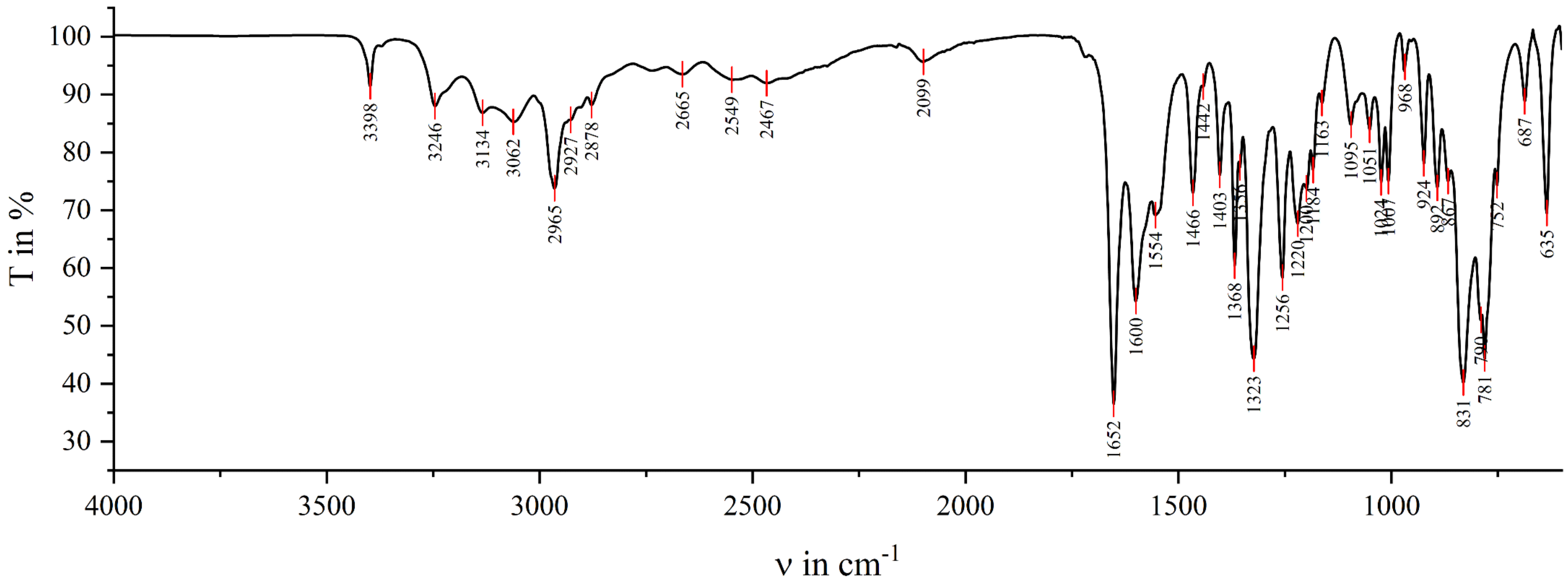

Figure S20. IR (ATR) spectrum of (Aib)SiMe$_2$(NH$_2$*n*Pr).

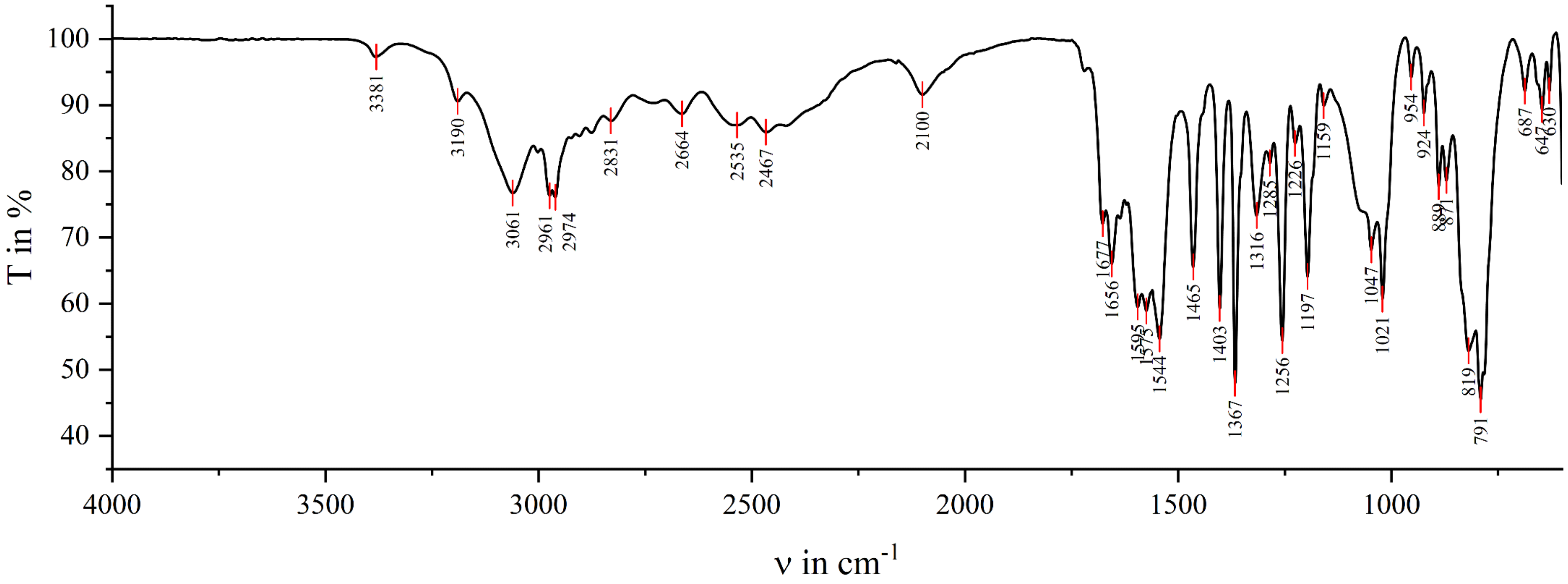

Figure S21. IR (ATR) spectrum of (Aib)SiMe$_2$(HPyr).

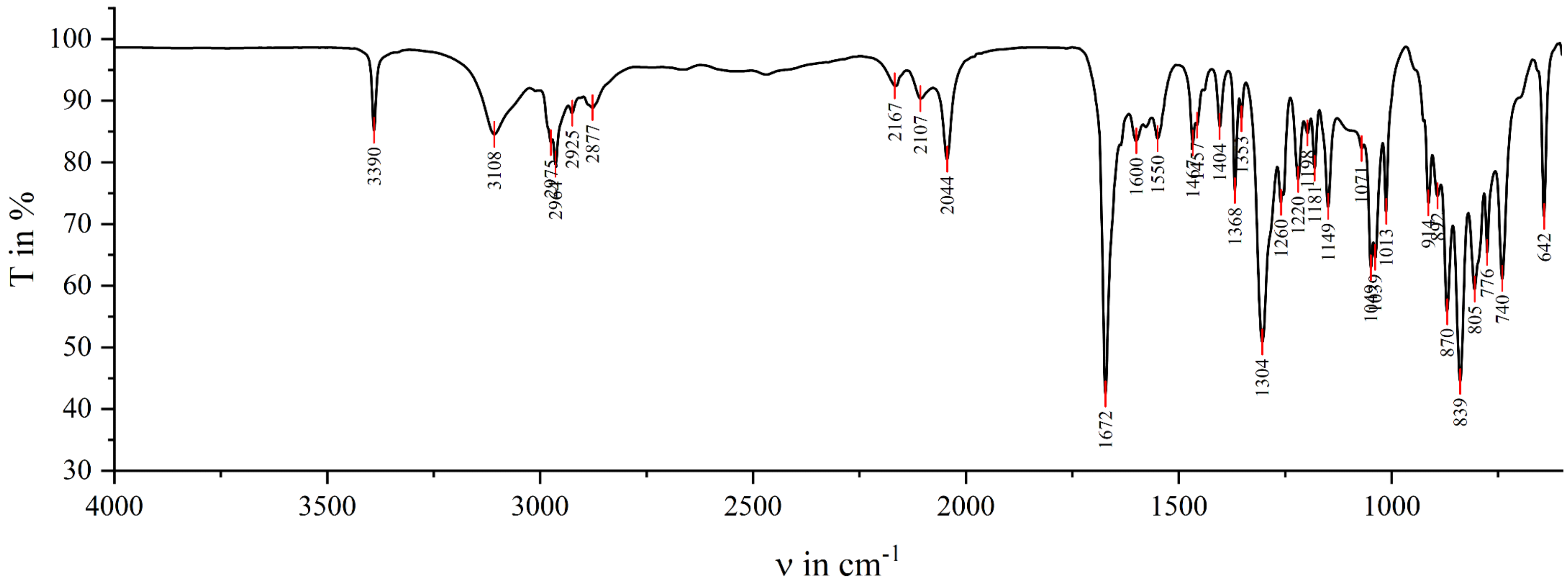

Figure S22. IR (ATR) spectrum of (Aib)SiMeH(HPyr).

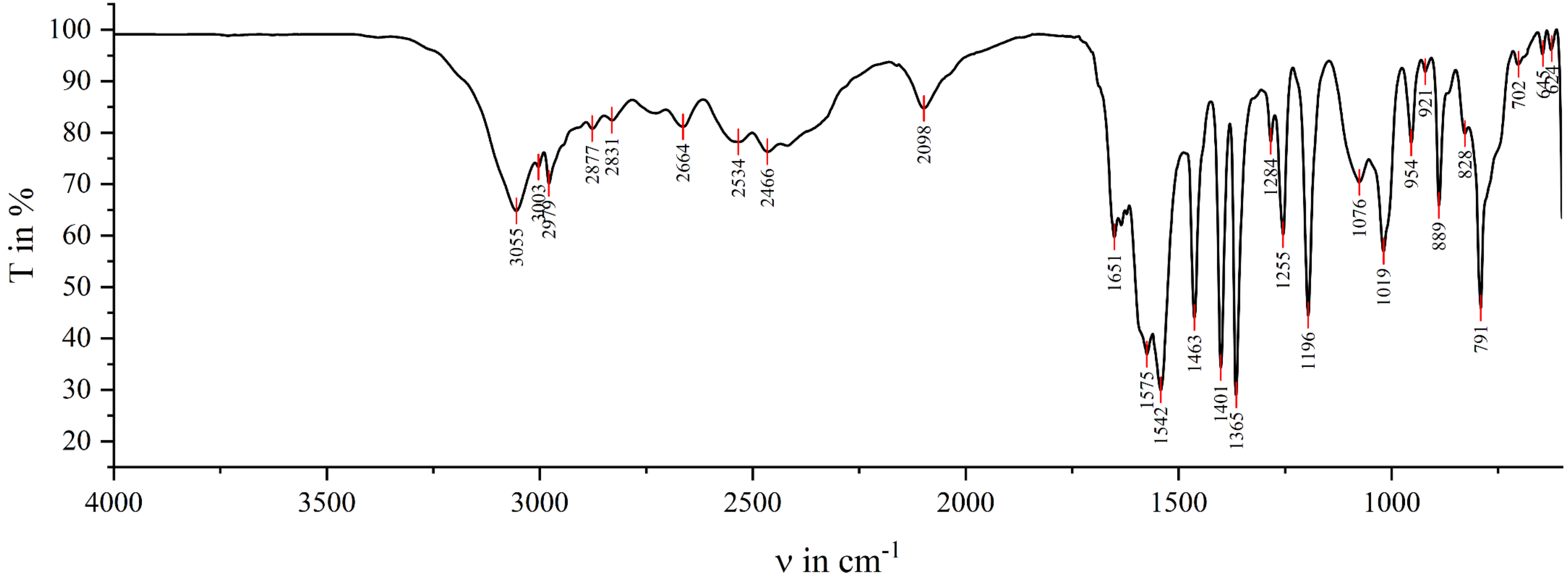

Figure S23. IR (ATR) spectrum of (Aib)SiMeVi(HPyr).

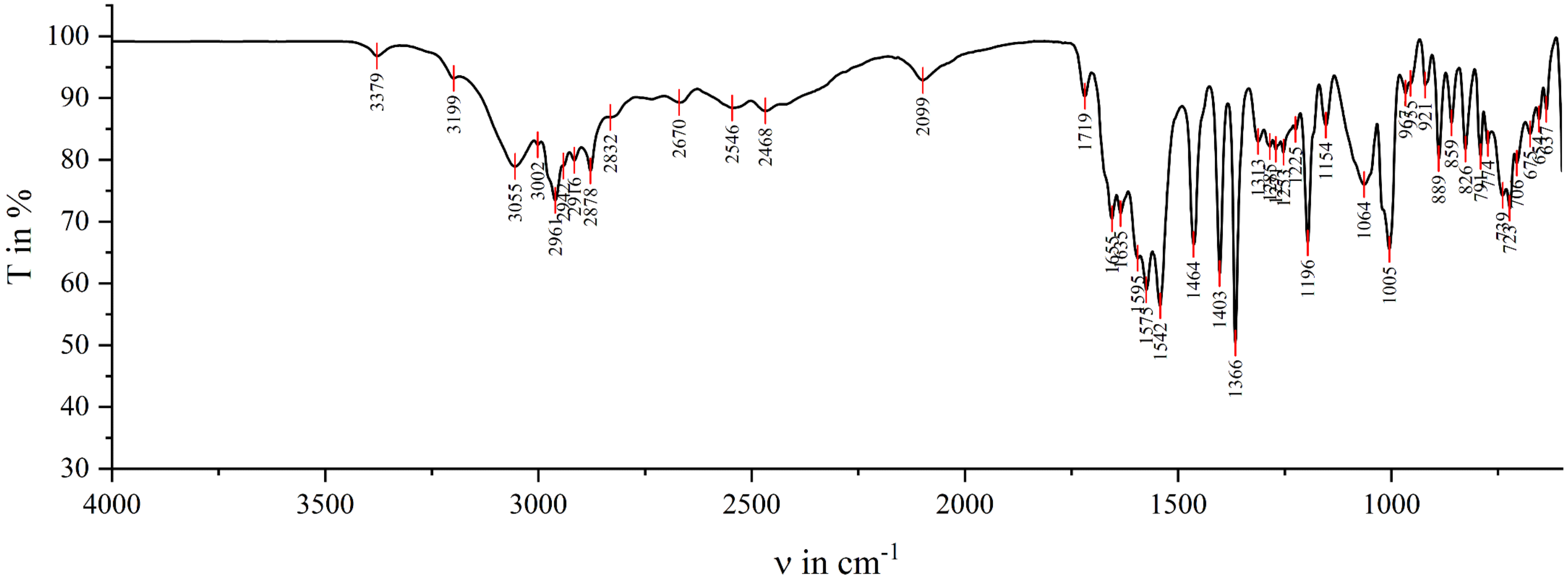

Figure S24. IR (ATR) spectrum of (Aib)$SiEt_2$(HPyr).

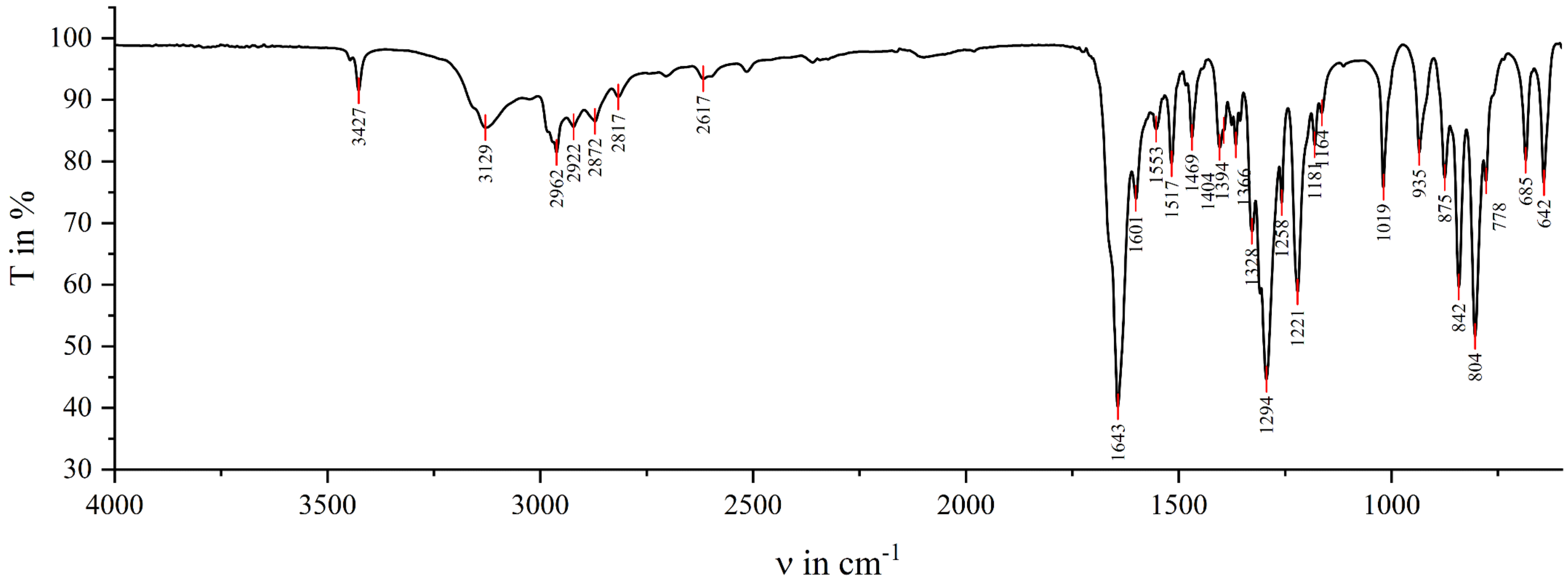

Figure S25. IR (ATR) spectrum of [tBuNH$_3$][(Aib)$_2$SiMe].

Syntheses of $SiMe_3(Im)$ and $SiMe_2(Im)_2$

Synthesis of $SiMe_3(Im)$: Imidazole (HIm, 13.02 g, 191.2 mmol) was placed in a three-neck flask with reflux condenser and dropping funnel, was evacuated and then set under an argon atmosphere. Then, *n*-hexane (250 mL) and the auxiliary base triethylamine (NEt_3, 22.92 g, 226.5 mmol) were added. With continuous stirring and cooling of that mixture in an ice bath, a solution of the chlorosilane (Me_3SiCl, 20.77 g, 191.2 mmol) in *n*-hexane (50 mL) was then slowly added using the dropping funnel. Upon completed addition of the chlorosilane, the reaction mixture was heated under refluxed for 5 h to afford a thick white suspension. Upon cooling to room temperature, the precipitate was filtered and washed with *n*-hexane (ca. 4 x 10 mL). From the combined filtrate and washings, the volatiles were removed by condensation into a cold trap. The product remained in the flask as a slightly oily light-yellow liquid. Yield: 24.98 g, 178.1 mmol, 93%. bp: 212.7 °C.

Synthesis of $SiMe_2(Im)_2$: The previously synthesized imidazolyltrimethylsilane ($SiMe_3(Im)$, 8.67 g, 61.8 mmol) was placed in a Schlenk tube, equipped with a magnetic stirring bar under argon atmosphere. Dichlorodimethylsilane (Me_2SiCl_2, 3.98 g, 30.8 mmol) was then added using a syringe, and the mixture was stirred briefly at room temperature to afford a clear solution. Upon storage at room temperature overnight, a crystalline solid had formed. The supernatant was transferred into a second Schlenk tube using a syringe, and the remaining volatiles (essentially Me_3SiCl) were removed in a vacuum. From the supernatant, the volatiles were also removed in a vacuum, and crystallization of the resultant oil was initiated using thermal shock (the Schlenk tube was briefly immersed in liquid nitrogen). The resulting batches of beige solid were washed with a small amount of *n*-hexane (ca. 2 x 2 mL) and dried in vacuum. Yield: 5.23 g, 27.2 mmol, 88%. mp: 47–50 °C.

Comparison of selected parameters of the crystal structure refinement of compound (Aib)$SiMe_2$(HPyr) without and with refinement of the disorder of the pyrrolidine backbone

Upon initial refinement of the crystal structure of compound (Aib)$SiMe_2$(HPyr), the highest residual electron density peak (+0.25 eÅ^{-3}) was located in close proximity to pyrrolidine carbon atom C9 (Figure S26, left). In spite of the low intensity of this peak, a disorder model was refined, which includes the adjacent atom C8 (disorder group C9a–C8a, Figure S26, right). The atoms N2a, C7a and C10a of the disorder group as well as their corresponding parent sites N2, C7 and C10, respectively, were fixed to identical coordinates, just the H atoms of C7a and C10a (included in the refinement in idealized positions, riding model, code AFIX 23) were allowed to adhere to the requirements of the disorder group. Chemically identical bonds were restrained to the same bond length (i.e., C7–C8 = C7a–C8a = C9–C10 = C9a–C10a, and C8–C9 = C8a–C9a). Because of the low site occupancy of the disorder group, thermal displacement parameters of an atom in the disorder group and its parent atom were kept equal. In spite of the low intensity of the initial residual electron density peak of 0.25 eÅ^{-3}, refinement revealed a site occupancy of 12(1)% of the disorder part. Table S1 lists a comparison of selected parameters before and after refinement of this disorder.

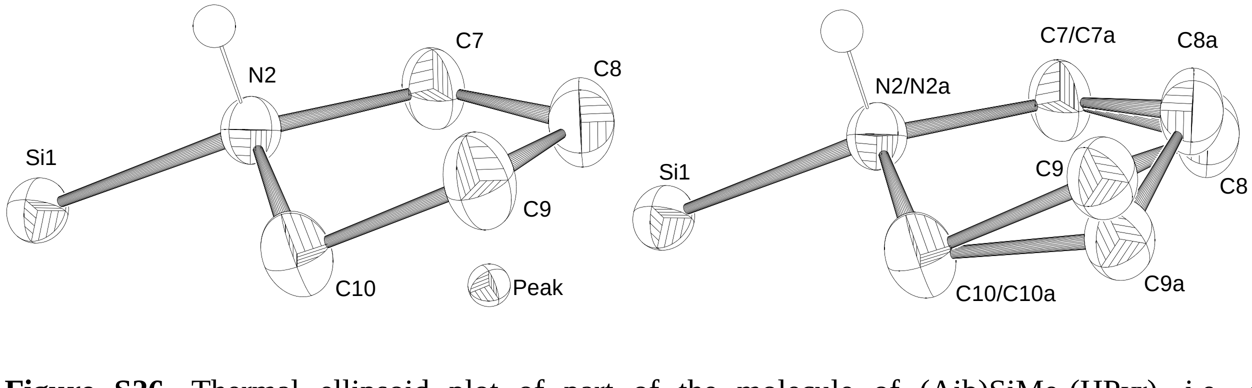

Figure S26. Thermal ellipsoid plot of part of the molecule of (Aib)$SiMe_2$(HPyr), i.e., the pyrrolidine ligand at the Si atom, before refinement of the pyrrolidine disorder and showing the location of the highest residual electron density peak (left) and upon refinement of the disorder (right). C-bound H atoms were omitted for clarity.

Table S1. Comparison of selected parameters before and after refinement of the disorder in the pyrrolidine ring of (Aib)$SiMe_2$(HPyr).

**Parameter**	**before**	**after**
$R1(I>2\sigma(I))$	0.0353	0.0344
$R1$(all data)	0.0477	0.0467
$wR2(I>2\sigma(I))$	0.0907	0.0877
$wR2$(all data)	0.0956	0.0924
Highest residual peak / eÅ^{-3}	+0.25	+0.21
Deepest residual hole / eÅ^{-3}	–0.21	–0.21
U_{iso} (C8)	0.0373(3)	0.0354(6)
U_{iso} (C9)	0.0396(4)	0.0334(6)
XYZ (C8)	0.7807(2) 0.5347(1) 0.8259(2)	0.7808(2) 0.5343(2) 0.8235(4)
XYZ (C9)	0.6897(2) 0.4851(1) 0.8940(2)	0.6910(2) 0.4867(3) 0.8969(3)
C7–C8 / Å	1.526(2)	1.5235(15)
C8–C9 / Å	1.508(2)	1.524(2)
C9–C10 / Å	1.5141(19)	1.5225(15)
C7-C8-C9 / °	105.97(11)	104.96(18)
C8-C9-C10 / °	104.13(12)	103.60(15)
N2-C7-C8 / °	106.92(11)	107.55(13)
N2-C10-C9 / °	104.46(11)	103.62(15)

<u>Space Fill view of a molecule of (Aib)SiMe$_2$(H$_2$N*n*Pr)</u>

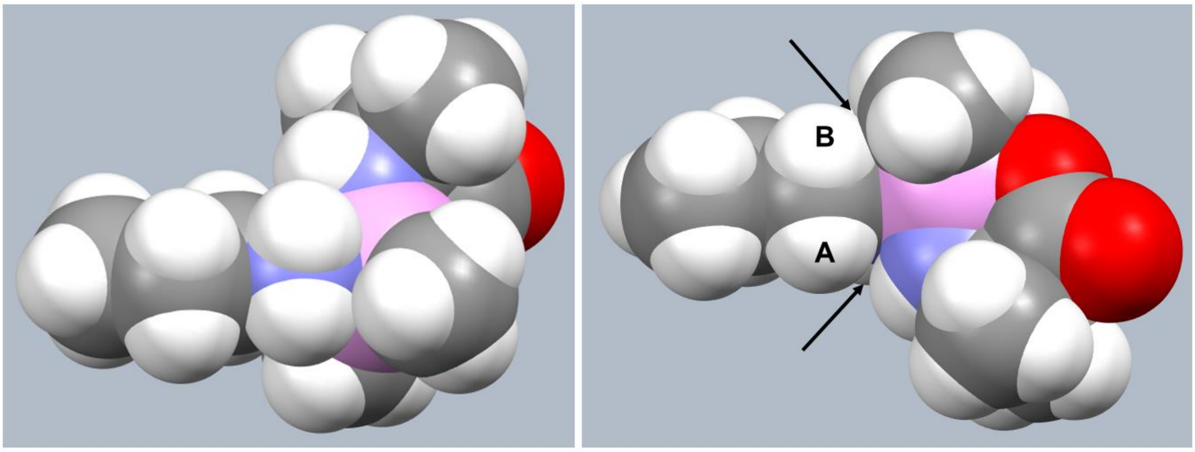

Figure S27. Space-Fill representation of one of the two crystallographically independent molecules of the crystal structure of (Aib)SiMe$_2$(H$_2$N*n*Pr) from two different perspectives, related to one another by a 180° rotation of the molecule about the in-plane horizonal axis. Left: View of the NH$_2$ group attached to the Si atom. Right: View of the α-H atoms of the propylamine moiety.

The propylamine NH$_2$ group (Figure S27, left) is flanked by the two Si-bound CH$_3$ groups around one of the NH sites, whereas the other NH is less crowded. The room available in the closer proximity of the amine N atom gives an idea as to why secondary amines of limited steric demand (i.e., pyrrolidine) may form that kind of adducts as well. The α-H atom **A** (Figure S27, right) is not at close contact with any adjacent group (no contact to the neighboring NH moiety of the (Aib) ligand), which indicates room for accommodating slightly bulkier groups (such as CH$_3$) at site **A**. The α-H atom **B** is at close contact with one of the Si-bound methyl groups. There is no room for bulkier groups (such as CH$_3$) at site **B**.

Evaluation of the tensors of ^{29}Si Chemical Shift Anisotropy (CSA)

^{29}Si CP/MAS spinning side band spectra of compounds (Aib)SiMe$_2$(HPyr), (Aib)SiMeH(HPyr), (Aib)SiMe$_2$(H$_2$N*n*Pr), (Aib)SiEt$_2$(HPyr) and (Aib)SiMe$_2$(HIm)·CHCl$_3$ were recorded at spinning frequencies of 3 kHz, and in case of (Aib)SiMeVi(HPyr) at 2 kHz, to obtain a suitable number of spinning side bands for these analyses. Because of the signal shapes of both the isotropic signals and spinning side bands, which are patterned by ^{29}Si-^{14}N residual dipolar coupling, strong smoothing (LB = 50) was applied to obtain signal shapes which allow for automatic fitting with a Lorentz-Gauss-model to obtain the individual intensities of isotropic signal and the associated spinning side bands for analysis with DMFIT (Massiot, D.; Fayon, F.; Capron, M.; King, I.; Le Calvlé, S.; Alonso, B.; Durand, J.-O.; Bujoli, B.; Gan, Z.; Hoatson, G. Modeling one and two-dimensional Solid State NMR spectra. *Magn. Reson. Chem.* **2002**, *40*, 70–76. https://doi.org/10.1002/mrc.984). Alternatively, the intensities could be derived by integration of the spectra and used for CSA tensor analysis with HBA (Eichele, K. *Herzfeld-Berger Analysis Program*; HBA (Version 1.7.3 23.8.2012), University of Tübingen: Tübingen, Germany, 1995 and 2012). Comparison of both approaches was performed for (Aib)SiMe$_2$(HPyr) (Table S2) and delivered similar results within the margin of error (standard deviation of 0.5 to 0.7 ppm for principal components and span, 0.01 for skew). Therefore, the approach of smoothing, automatic fitting and analysis with DMFIT was applied to all spectra under investigation. (Note: The strong smoothing of the spectra alters the position of the maximum peak, it is slightly shifted upfield with respect to the $\delta_{iso(max)}$ reported in the paper.) The spectra of the six compounds (bottom, blue: experimental spectrum upon smoothing; top, red: modeled spectrum using the CSA tensor principal values obtained by DMFIT analysis) are shown in Figures S28–S33.

Table S2. Results of ^{29}Si CSA tensor analyses of compounds (Aib)SiMe$_2$(HPyr), (Aib)SiMeH(HPyr), (Aib)SiMe$_2$(H$_2$N*n*Pr), (Aib)SiEt$_2$(HPyr), (Aib)SiMeVi(HPyr) and (Aib)SiMe$_2$(HIm)·(CHCl$_3$). (Note: In case of (Aib)SiMe$_2$(H$_2$N*n*Pr), the two lines of data entries correspond to the two signals observed for the two crystallographically independent Si sites.)

**Sample**	**Method**	δ_{iso}	δ_{11}	δ_{22}	δ_{33}	**Ω**	**κ**
(Aib)SiMe$_2$(HPyr)	HBA (integration of original spectrum)	–71.64	45.6	–128.9	–131.6	177.1	–0.97
	HBA (integration upon smoothing applied)	–71.7	45.4	–128.4	–132.1	177.6	–0.96
	DMFIT	–71.7	45.6	–128.6	–132.1	177.7	–0.96
(Aib)SiMeH(HPyr)	DMFIT	–81.0	22.4	–121.9	–143.4	165.8	–0.74
(Aib)SiMe$_2$(H$_2$N*n*Pr)	DMFIT	–73.5	59.4	–123.1	–156.8	216.2	–0.69
		–76.0	52.4	–129.2	–151.1	203.5	–0.79
(Aib)SiEt$_2$(HPyr)	DMFIT	–63.0	56.9	–119.4	–126.6	183.5	–0.92
(Aib)SiMeVi(HPyr)	DMFIT	–81.4	34.4	–125.4	–153.1	187.5	–0.70
(Aib)SiMe$_2$(HIm)·(CHCl$_3$)	DMFIT	–71.6	55.6	–115.0	–155.4	211.0	–0.62

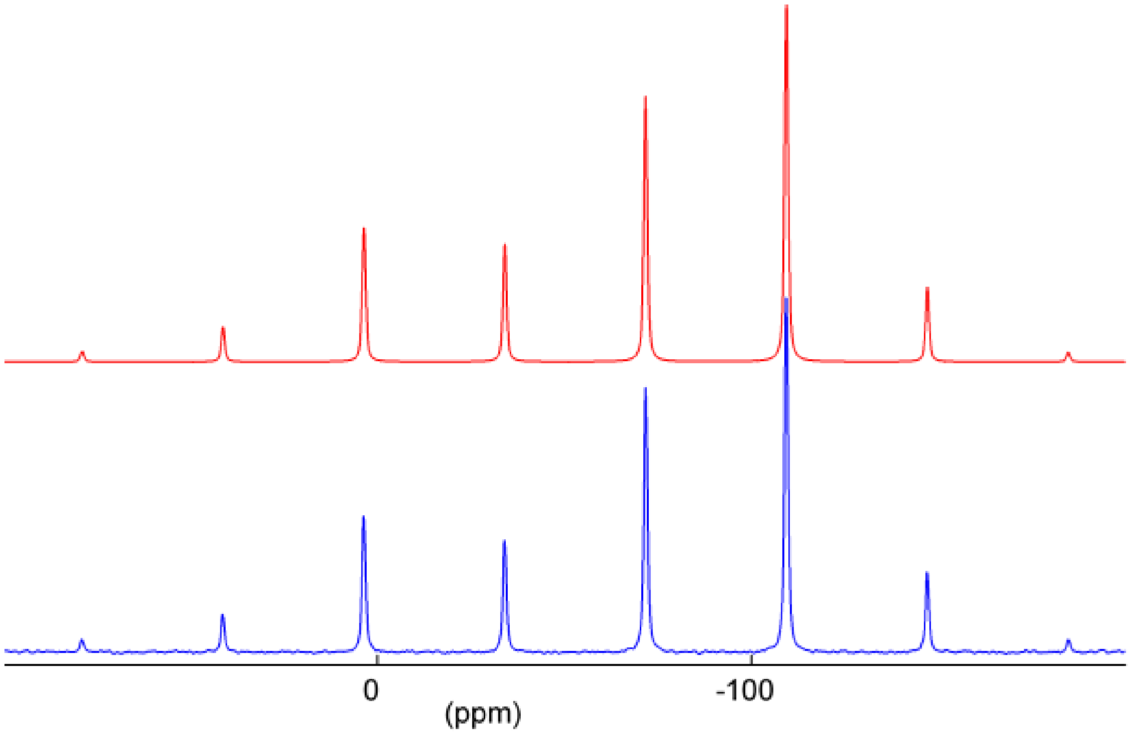

Figure S28. ^{29}Si CP/MAS NMR spectrum of (Aib)$SiMe_2$(HPyr) (ν_{rot} = 3 kHz). Bottom, blue: experimental spectrum upon smoothing; top, red: modeled spectrum using the CSA tensor principal values obtained by DMFIT analysis (cf. Table S2).

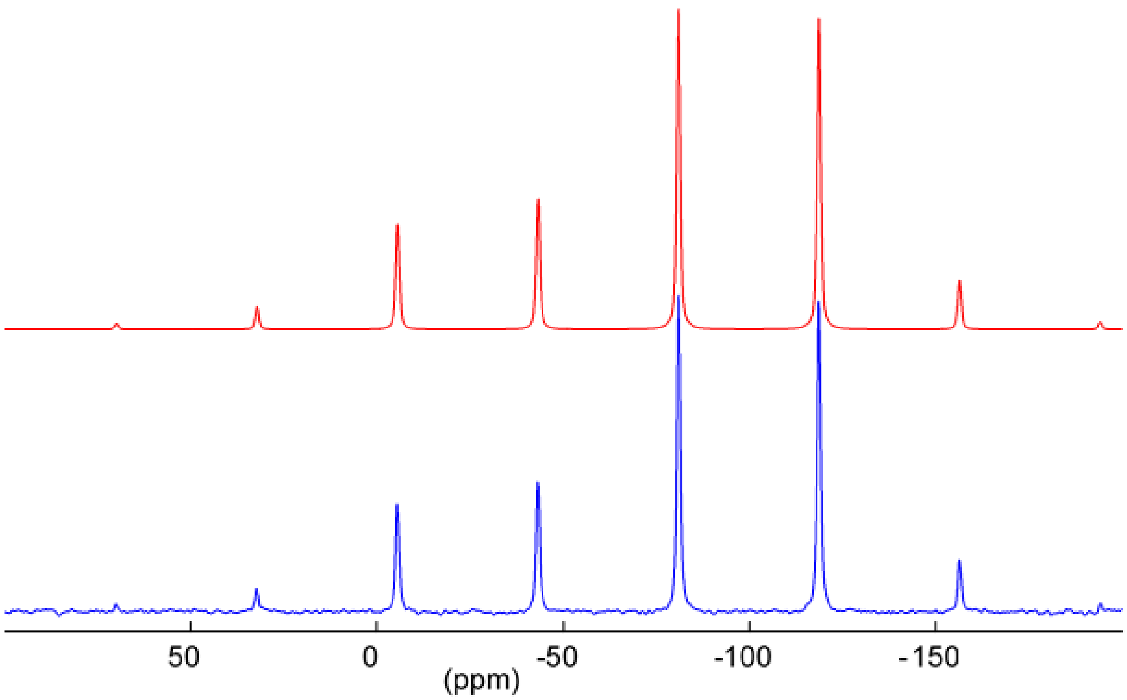

Figure S29. ^{29}Si CP/MAS NMR spectrum of (Aib)SiMeH(HPyr) (ν_{rot} = 3 kHz). Bottom, blue: experimental spectrum upon smoothing; top, red: modeled spectrum using the CSA tensor principal values obtained by DMFIT analysis (cf. Table S2).

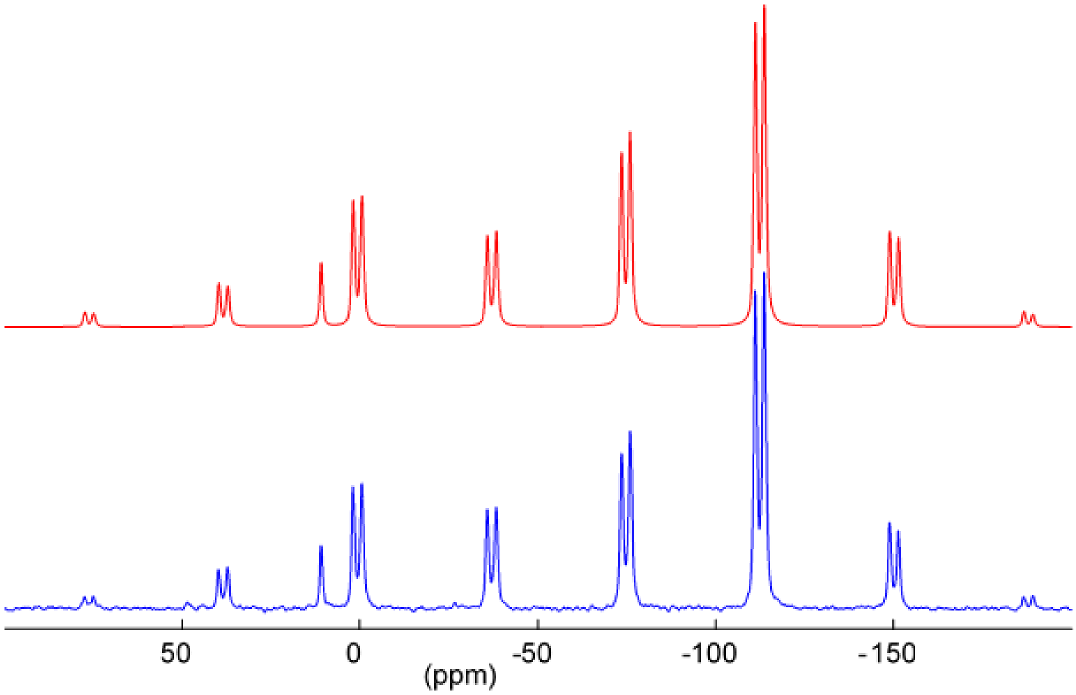

Figure S30. ^{29}Si CP/MAS NMR spectrum of (Aib)SiMe$_2$(H$_2$N*n*Pr) (ν_{rot} = 3 kHz). Bottom, blue: experimental spectrum upon smoothing; top, red: modeled spectrum using the CSA tensor principal values obtained by DMFIT analysis (cf. Table S2). The signal of the unknown contaminant (at 10.9 ppm) was treated individually (its isotropic signal was fitted) and was included in the modeled spectrum.

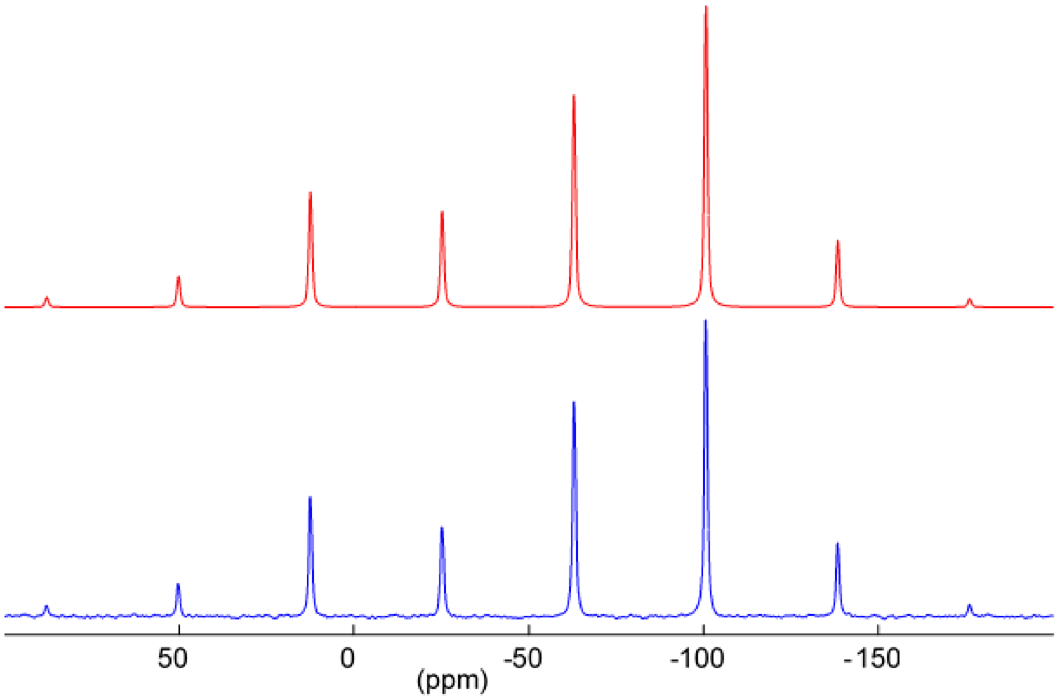

Figure S31. ^{29}Si CP/MAS NMR spectrum of (Aib)SiEt$_2$(HPyr) (ν_{rot} = 3 kHz). Bottom, blue: experimental spectrum upon smoothing; top, red: modeled spectrum using the CSA tensor principal values obtained by DMFIT analysis (cf. Table S2).

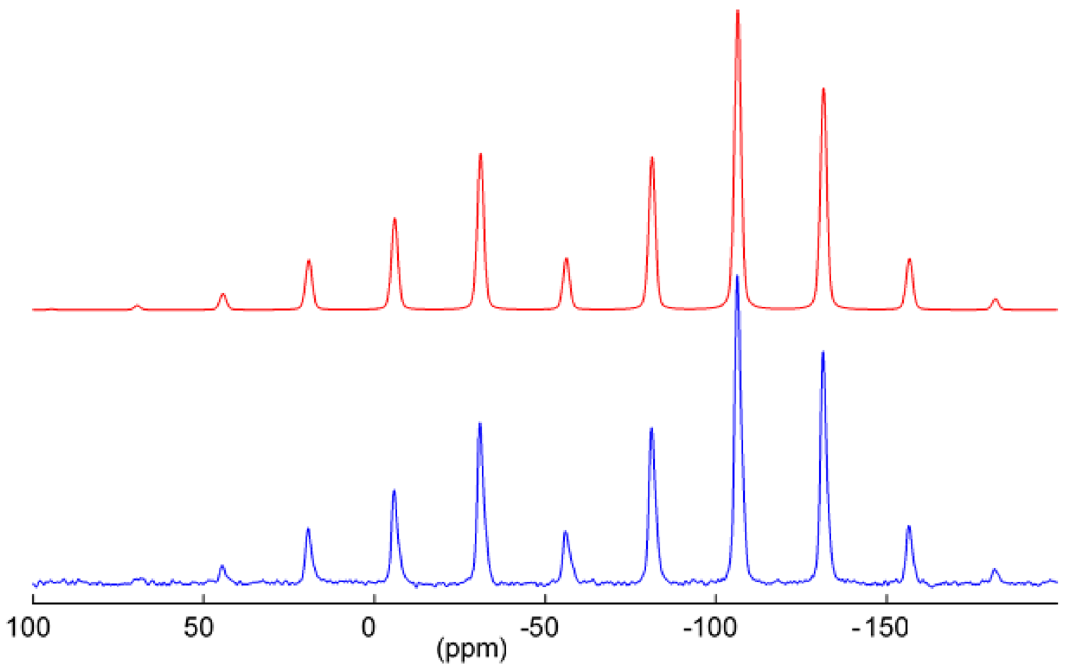

Figure S32. ^{29}Si CP/MAS NMR spectrum of (Aib)SiMeVi(HPyr) (ν_{rot} = 2 kHz). Bottom, blue: experimental spectrum upon smoothing; top, red: modeled spectrum using the CSA tensor principal values obtained by DMFIT analysis (cf. Table S2).

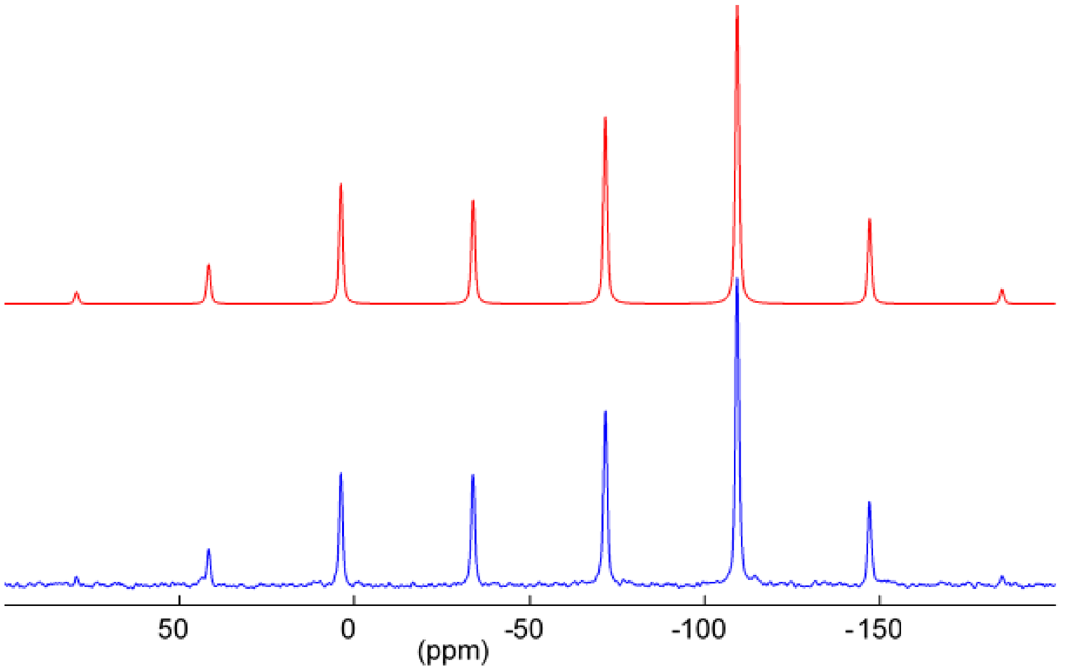

Figure S33. ^{29}Si CP/MAS NMR spectrum of (Aib)$SiMe_2$(HIm)·($CHCl_3$) (ν_{rot} = 3 kHz). Bottom, blue: experimental spectrum upon smoothing; top, red: modeled spectrum using the CSA tensor principal values obtained by DMFIT analysis (cf. Table S2).
